# Risk of Guideline Overselling and Certainty Inflation in GPT-5 Responses: A Statement-Based Analysis of Four ESGE Colorectal Guidelines

**DOI:** 10.1055/a-2889-0169

**Published:** 2026-06-16

**Authors:** Marcello Maida, Antonio Facciorusso, Lorenzo Fuccio, Tony Tham, Alessandro Vitello, Giulio Calabrese

**Affiliations:** 1 Department of Medicine and Surgery 217140 Kore University of EnnaEnnaSicilyItaly; 2 Gastroenterology Unit 73129 Umberto I HospitalEnnaSicilyItaly; 3 Gastroenterology Unit, Department of Experimental Medicine 18976 Università del SalentoLecceItaly; 4 Department of Gastroenterology 9296 University of BolognaBolognaEmilia-RomagnaItaly; 5 Division Of Gastroenterology 155303 Ulster HospitalDundonaldNorthern IrelandUnited Kingdom of Great Britain and Northern Ireland; 6 Clinical Medicine and Surgery 9307 University of Naples Federico IINaplesCampaniaItaly

**Keywords:** artificial intelligence, clinical practice guidelines, endoscopy, decision-making, overselling

## Abstract

**Introduction** Large language models (LLMs) are increasingly used for clinical decision support and show high adherence to clinical practice guidelines. However, LLM-generated responses may translate guideline recommendations into definitive clinical advice without adequately reflecting the underlying strength of recommendations or the quality of supporting evidence. This study aimed to evaluate the risk of guideline overselling in GPT-generated responses based on guidelines.

**Methods** Four ESGE colorectal guidelines were selected. All guideline statements were extracted together with their strength of recommendation and the quality of evidence. For each statement, a clinical vignette and related question were submitted to GPT-5 using both generic and guideline-oriented prompts. Three independent experts assessed each response for adherence to guideline content and calibration of wording.

**Results** Overall, GPT-generated responses showed high adherence to guideline content with generic prompts (54/59, 91.5%), whereas calibration to recommendation strength and evidence quality was lower (44/59, 74.6%). Calibration was significantly reduced for weak versus strong recommendations (33.3% vs. 86.4%, *P* < 0.001) and for statements supported by low-quality versus high- or moderate-quality evidence (44.4% vs. 90.2%, *P* < 0.001). After prompt calibration experiment, adherence increased to 96.6% (57/59), while calibration markedly improved to 94.9% (56/59), mainly through reduced overly assertive wording for low-certainty recommendations.

**Conclusions** GPT-5 demonstrated high guideline adherence but limited calibration to the quality of evidence underlying individual recommendations, with the potential to amplify the perceived certainty of low-quality evidence. Explicit incorporation of recommendation strength and evidence quality within prompts substantially improves calibration and may mitigate this risk in clinical use.

## Introduction

Large language models (LLMs) are emerging as a potentially transformative tool for clinical decision support, with rapidly expanding applications across medical specialties, including gastroenterology. 1 2 3 In this regard, several lines of evidence have shown that LLM-generated responses demonstrate high concordance with clinical practice guidelines, including in gastrointestinal endoscopy, where high adherence has been reported for recommendations such as post-colonoscopy surveillance and follow-up strategies. 4 5

Despite this apparent effectiveness, an important concern relates not to whether LLMs adhere to guideline statements, but to how recommendations are conveyed. LLM-generated responses may automatically translate guideline statements into definitive clinical advice without adequately reflecting the underlying strength of recommendations or the quality of supporting evidence. This issue is particularly relevant in scenarios governed by weak recommendations or supported by low-quality evidence, where guideline panels intentionally adopt cautious language and emphasize conditional applicability.

Over recent years, the widespread adoption of the GRADE methodology has reinforced this distinction by explicitly separating the strength of recommendations from the quality of evidence. 6 7 Weak or conditional recommendations acknowledge uncertainty, variability in patient values, or limitations in the evidence base, and are intended to support individualized decision-making rather than prescriptive clinical actions. Preserving this nuance is central to evidence-based medicine and to the methodological rigor of guideline development.

However, even when guideline content is accurately reproduced, LLMs may present weak or low-certainty recommendations using assertive wording, thereby amplifying their perceived certainty.

Unlike previous studies primarily focused on guideline adherence, 8 the present analysis specifically evaluates the alignment between response wording and the underlying strength of recommendation and the quality of evidence. This phenomenon, here referred to as guideline overselling (i.e., certainty inflation), may contribute to uncritical adherence to recommendations that were not intended to be universally applied, potentially undermining clinicians’ critical appraisal of guideline statements. This phenomenon should be distinguished from related ones such as hallucinations, overconfidence, or misinterpretation of recommendations.

In this study, we aimed to evaluate the risk of guideline overselling in GPT-generated responses to clinical questions derived from four ESGE colorectal guidelines, with a specific focus on the alignment between response wording, strength of recommendation, and quality of evidence.

## Methods

### Guideline Selection and Vignette Development

Four ESGE clinical practice guidelines addressing colorectal cancer surveillance, 9 10 familial colorectal cancer risk, 11 and the endoscopic management of colorectal lesions 12 were considered for analysis. All guideline statements were systematically extracted together with their reported strength of recommendation and quality of evidence, as defined by the guideline panels.

For each guideline statement, a clinical vignette reflecting a simplified clinical scenario was developed de novo by the authors (MM, AV, GC) based on the corresponding recommendation, to ensure consistency and comparability across items. Vignettes were not derived from real clinical cases and were not formally piloted, as the aim was to create standardized test conditions rather than reproduce real-world variability. Reviewers were aware of the underlying recommendation strength and evidence quality, as calibration required direct comparison with the reference standard.

### AI Assessment and Prompting Strategy

All clinical vignettes were initially submitted to GPT-5 (version 5.2; OpenAI) using a generic prompt designed to approximate a typical clinician query, formulated as follows: “Based on current clinical practice guidelines, what is the most appropriate management approach in this situation?” ( **Supplementary Material A** ).

All queries were performed using GPT-5 (version 5.2; OpenAI) via the standard web interface between December 15 and December 30, 2025. Model parameters such as temperature were not user-configurable in this setting. A single response was generated per prompt for each vignette-question pair, without repeated runs.

In addition, a proof-of-concept prompt calibration experiment was conducted by resubmitting all vignette-question pairs using a modified, guideline-oriented prompt explicitly formulated as follows: “Based on current clinical practice guidelines, provide the most appropriate management approach. When formulating your answer, explicitly consider the strength of the recommendation and the quality of the supporting evidence, and calibrate the wording of your response accordingly” ( **Supplementary Material B** ).

### Outcomes assessment and definition

Three independent expert reviewers with experience in colorectal endoscopy and guideline development (MM, LF, AF) evaluated each GPT-generated response.

Responses were assessed using a predefined dichotomous scoring system (0/1) across two domains: adherence and calibration. This approach was chosen to ensure a standardized and reproducible evaluation framework across reviewers, allowing a clear distinction between calibrated and noncalibrated responses in relation to recommendation strength and evidence quality. Adherence was defined as concordance between the response content and the corresponding guideline statement. Calibration was defined as appropriate alignment of the wording and decisional tone of the response with the strength of recommendation and the quality of evidence supporting the statement. In particular, responses were considered not calibrated when weak or low-certainty recommendations were presented using overly assertive or prescriptive language (e.g., use of definitive terms such as “should” or “must” for weak recommendations). A detailed coding guide with operational definitions and illustrative examples used for calibration assessment is provided in **Supplementary Material C** .

For each item, the final score was assigned according to a majority rule (agreement of at least two of three reviewers). Inter-rater reliability among raters was assessed using Fleiss’ κ to quantify categorical agreement beyond chance, interpreted according to Landis and Koch’s criteria (<0 poor, 0.00–0.20 slight, 0.21–0.40 fair, 0.41–0.60 moderate, 0.61–0.80 substantial, 0.81–1.00 almost perfect). 13

### Statistical Analysis

Descriptive statistics were used to summarize adherence and calibration rates. Categorical variables were summarized as frequency and percentage. As appropriate, comparisons between independent groups were performed using the Chi-square test. For paired comparisons between responses generated with generic and guideline-oriented prompts, McNemar’s exact test was used. A two-sided *P* value < 0.05 was considered statistically significant. All statistical analyses were performed using SPSS v. 30.0 for Macintosh (SPSS Inc., Chicago, USA).

## Results

A total of 59 guideline statements derived from four ESGE colorectal guidelines were evaluated ( **Supplementary Table 1** ). Inter-rater agreement among reviewers, assessed using Fleiss’ κ, showed almost perfect agreement for both adherence (κ = 0.845, 95% CI 0.70–1.00) and calibration (κ = 0.912, 95% CI 0.76–1.00). Overall, most responses were classified as adherent (54/59, 91.5%), whereas a lower proportion were classified as calibrated (44/59, 74.6%) ( Table 1 ). In addition, the distribution of ratings was similar across raters (Rater 1: 55/59; Rater 2: 54/59; Rater 3: 54/59 for adherence; Rater 1: 44/59; Rater 2: 45/59; Rater 3: 42/59 for calibration), supporting the interpretation of the kappa values and suggesting no relevant imbalance in rating distributions.

**Table 1:** Adherence and calibration of AI-generated responses to ESGE colorectal guideline statements.

	Number of statements	Adherence %	*P* value	Calibration %	*P* value
**Overall**	59	91.5% (54/59)	–	74.6% (44/59)	–
**Guidelines**					
1.Endoscopic surveillance after surgical or endoscopic resection for colorectal cancer	7	85.7% (6/7)	0.66	57.1% (4/7)	0.40
2.Post-polypectomy colonoscopy surveillance	8	100% (8/8)		62.5% (5/8)	
3.Endoscopic management of Lynch syndrome and of familial risk of colorectal cancer	15	86.7% (13/15)		86.7% (13/15)	
4.Colorectal polypectomy and endoscopic mucosal resection	29	93.1% (27/29)		75.9% (22/29)	
**Strength of recommendation**					
Strong	44	90.9% (40/44)	1.00	86.4% (38/44)	<0.001
Weak	15	93.3% (14/15)		33.3% (5/15)	
**Quality of evidence**				
High–Moderate	41	92.7% (38/41)	1.00	90.2% (37/41)	<0.001
Low	18	88.9% (16/18)		44.4% (8/18)	
**High vs. low certainty recommendations**					
Strength of recommendation strong + High/Moderate quality evidence	34	94.1% (32/34)	0.72	91.2% (31/34)	0.001
Strength of recommendation weak or low quality evidence	25	88.0% (22/25)		52.0% (13/25)	

Adherence and calibration did not significantly differ across the four guidelines analyzed, with adherence rates ranging from 85.7% to 100% ( *P* = 0.66) and calibration rates ranging from 57.1% to 86.7% ( *P* = 0.40).

When stratified by strength of recommendation, adherence remained similarly high for strong and weak recommendations (90.9% vs. 93.3%, *P* = 1.00). In contrast, calibration was significantly lower for weak compared with strong recommendations (33.3% vs. 86.4%, *P* < 0.001) ( Table 1 ). A comparable pattern was observed when statements were stratified by quality of evidence. Adherence did not differ between statements supported by high- or moderate- versus low-quality evidence (92.7% vs. 88.9%, *P* = 1.00), whereas calibration was significantly reduced for statements supported by low-quality evidence (44.4% vs. 90.2%, *P* < 0.001) ( Table 1 ).

**Fig. 1:**
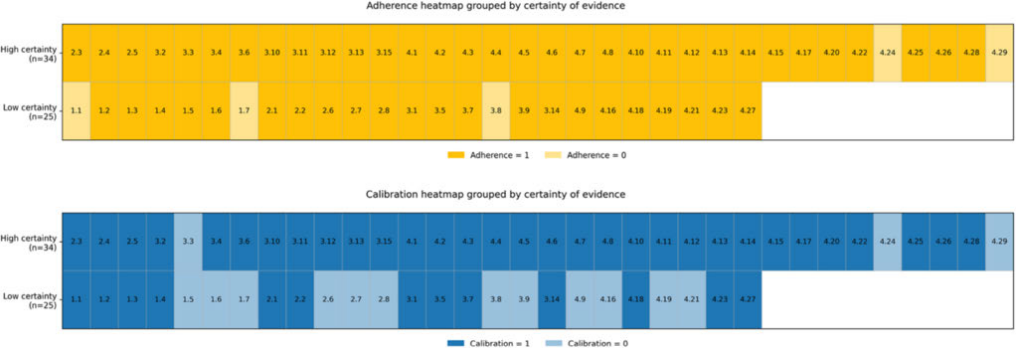
Heatmaps showing adherence (top panel) and calibration (bottom panel) of AI-generated responses to guideline statements, grouped by certainty of evidence. Each cell corresponds to a single guideline statement, with color intensity indicating high (dark) or low (light) adherence or calibration.

Finally, when statements were grouped according to overall certainty, calibration was markedly higher for strong recommendations supported by high- or moderate-quality evidence compared with weak recommendations and/or those based on low-quality evidence (91.2% vs. 52.0%, *P* = 0.001), while adherence remained consistently high in both groups (94.1% vs. 88.0%, *P* = 0.72) ( Fig. 1 ).

**Fig. 2:**
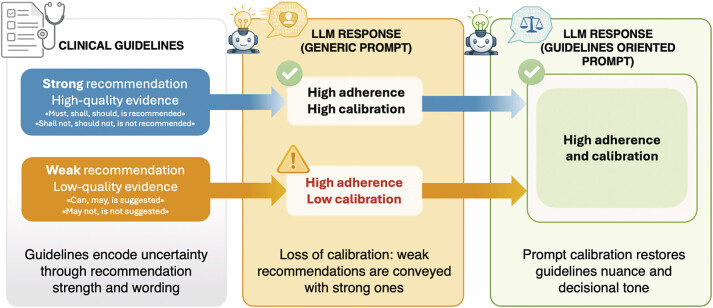
Effect of prompt design on calibration of LLM responses to clinical guidelines.

When guideline-oriented prompt calibration was applied, overall adherence to guideline content increased from 91.5% (54/59) to 96.6% (57/59), while calibration of the wording to the strength of recommendation and quality of evidence improved from 74.6% (44/59) to 94.9% (56/59) ( Table 2 ). The improvement in adherence and calibration with the guideline-oriented prompt was further assessed using McNemar’s exact test. While the increase in adherence was not statistically significant ( *P* = 0.25), the improvement in calibration was statistically significant ( *P* < 0.001). The improvement in calibration was primarily driven by a reduction in overly assertive wording for weak recommendations and statements supported by low-certainty evidence ( Fig. 2 ).

**Table 2:** Comparison of guideline adherence and calibration between original (generic) prompts and modified (guideline-oriented) prompts.

	Original prompts	Modified prompts	Δ statement	Δ %	*P* value*
**Overall**					
Adherence %	91.5% (54/59)	96.6% (57/59)	+3	+5.1%	0.25
Calibration %	74.6% (44/59)	94.9% (56/59)	+12	+20.3%	<0.001
**Guideline 1.Endoscopic surveillance after surgical or endoscopic resection for colorectal cancer**					
Adherence %	85.7% (6/7)	100% (7/7)	+1	+14.3%	1.00
Calibration %	57.1% (4/7)	100% (7/7)	+3	+42.9%	0.25
**Guideline 2.Post-polypectomy colonoscopy surveillance**					
Adherence %	100% (8/8)	100% (8/8)	0	0%	1.00
Calibration %	62.5% (5/8)	100% (8/8)	+3	+37.5%	0.25
**Guideline 3.Endoscopic management of Lynch syndrome and of familial risk of colorectal cancer**					
Adherence %	86.7% (13/15)	100% (15/15)	+2	+13.3%	0.50
Calibration %	86.7% (13/15)	93.3% (14/15)	+1	+6.6%	1.00
**Guideline 4.Colorectal polypectomy and endoscopic mucosal resection**					
Adherence %	93.1% (27/29)	93.1% (27/29)	0	+0%	1.00
Calibration %	75.9% (22/29)	93.1% (27/29)	+5	+17.2%	0.063

* *P* values were calculated using McNemar’s exact test.

The observed improvement in calibration with the guideline-oriented prompt should be interpreted as a proof-of-concept of prompt calibration, as the model was explicitly instructed to align its wording with recommendation strength and evidence quality. While this effect is inherently dependent on prompt design, it highlights the critical role of well-structured prompts in guiding LLM outputs and supports the need for greater awareness and training among clinicians in prompt formulation. In addition, complementary system-level strategies, such as embedded prompts within clinical AI tools, retrieval-augmented generation approaches that incorporate guideline content and associated metadata directly into the model context, or structured output formats separating recommendations from evidence quality, may further enhance calibration and help mitigate certainty inflation in real-world applications.

## Discussion

In this study, GPT-generated responses demonstrated high adherence to clinical practice guidelines across all evaluated statements, regardless of the underlying strength of recommendation or quality of evidence. This finding is consistent with previous reports showing that LLMs can accurately retrieve and reproduce guideline-based content in structured clinical domains, including gastrointestinal endoscopy and post-colonoscopy surveillance. 4 5

Taken together, these data confirm that LLMs are capable of providing guideline-concordant information across a broad spectrum of clinical scenarios.

However, beyond content adherence, our analysis highlights a critical and clinically relevant limitation related to calibration, defined as the appropriate alignment of the wording and decisional tone of a response with the strength of recommendation and the quality of supporting evidence. In detail, calibration was significantly lower for statements characterized by weak recommendations, low-quality evidence, or both. While adherence remained uniformly high, calibration declined markedly under conditions of greater uncertainty, indicating that LLM-generated responses tend to convey weak or low-certainty recommendations using overly assertive or prescriptive language.

These findings suggest that LLMs may exhibit a form of certainty inflation, whereby weak recommendations are presented using overly assertive language. As discussed above, this phenomenon is particularly concerning within the framework of evidence-based medicine and the GRADE methodology, where weak or conditional recommendations are intentionally formulated to preserve clinical judgment, acknowledge uncertainty, and promote individualized decision-making. By flattening the distinction between strong and weak recommendations, LLM-generated responses risk amplifying the perceived certainty of statements that guideline developers explicitly intended to be applied with caution, thereby contributing to certainty inflation.

At scale, this effect may have broader implications. The global accessibility of LLMs raises the possibility that weak recommendations and/or those supported by low-quality evidence could be disseminated worldwide as seemingly firm clinical directives, potentially undermining the methodological rigor of guideline development and reducing clinicians’ engagement in critical appraisal. Over time, this may contribute to uncritical adherence to recommendations supported by low-certainty evidence, with potential consequences for clinical practice, resource utilization, and patient outcomes.

This study has several limitations. First, the analysis was restricted to a single LLM (GPT-5), and four clinical guidelines. Subgroup analyses were based on relatively small sample sizes and may therefore be underpowered. As a result, the findings may not be generalizable to other models, guideline topics, or clinical domains. Second, the assessment of adherence and calibration was performed by three expert reviewers and therefore involved an element of subjectivity, despite the use of predefined dichotomous criteria and consensus adjudication. The use of a dichotomous scoring system may oversimplify the gradations of certainty expressed in LLM responses. More granular or linguistically informed approaches could provide a more nuanced assessment of calibration. In addition, calibration was assessed as a composite measure combining alignment with both recommendation strength and evidence quality, and therefore does not allow identification of which specific component contributed to miscalibration at the individual-response level.

Third, the study relied on structured clinical vignettes derived from guideline statements, which do not fully reflect the complexity, ambiguity, and variability of real-world clinical queries. This may further limit the generalizability of the findings to routine clinical practice. In addition, only a single response was generated for each vignette using the standard web interface. Given the nondeterministic nature of LLM outputs and the inability to control parameters such as temperature in this setting, the results may be subject to sampling variability, which may limit reproducibility.

Despite these limitations, the study provides novel insights into an underexplored dimension of LLM performance in clinical settings. Our findings indicate that LLMs can reliably adhere to guideline content but may struggle to appropriately communicate uncertainty when recommendations are weak or evidence is limited. This distinction is particularly relevant as LLMs are increasingly used to support clinical decision-making worldwide.

Given the rapid evolution of AI tools in clinical practice and the associated risks, guideline developers should also consider, when feasible, limiting the excessive number of recommendations supported by low-certainty evidence.

On the other side, clinicians should explicitly incorporate references to the strength of recommendations and the quality of evidence within their prompts, thereby encouraging responses that better reflect uncertainty and supporting more nuanced, evidence-informed clinical decision-making.

More broadly, these findings point to an unmet and often overlooked need for dedicated training of clinicians in the effective use of LLMs. Such training should focus on helping clinicians formulate appropriate, well-structured clinical questions, as the quality and relevance of AI-generated responses critically depend on how queries are framed. 14

## Supplementary Material

Ergänzendes Material Supplementary Material
