# Supplementary material for: Risk of Guideline Overselling and Certainty Inflation in GPT-5 Responses: A Statement-Based Analysis of Four ESGE Colorectal Guidelines
Source: Endosc Int Open. 2026 Jun 16;14:a28890169. doi: 10.1055/a-2889-0169 (PMC13289777; doi:10.1055/a-2889-0169)
Supplement: Supplementary file 1 — Ergänzendes Material [file 10-1055-a-2889-0169_28943875.pdf]

Supplementary Material A. Clinical vignettes and questions using the generic prompts.

Guideline 1. Endoscopic surveillance after surgical or endoscopic resection for colorectal cancer

Case 1.1

Vignette
A 64-year-old patient has undergone curative surgery for colorectal cancer, followed by appropriate oncological treatment. There are no current symptoms, and no evidence of local or distant disease has been reported since surgery.

Question
What would be the most appropriate management approach in this situation?

Most appropriate management: structured post-treatment surveillance

In an asymptomatic 64-year-old patient who has undergone **curative resection for colorectal cancer** and completed **appropriate oncological treatment**, with **no evidence of local or distant recurrence**, the correct approach is **active surveillance**, not further treatment.

A standard, guideline-concordant follow-up strategy includes:

1. **Clinical follow-up**

   * Regular outpatient visits with history and physical examination, typically every 3 to 6 months for the first 2 to 3 years, then less frequently up to 5 years.

2. **CEA monitoring**

  * Serum CEA every 3 to 6 months for the first 2 to 3 years, then every 6 months up to 5 years, if the tumor was CEA-producing.

3. **Imaging**

  * CT scan of chest, abdomen, and pelvis annually for at least 3 years in patients at moderate to high risk of recurrence.

4. **Endoscopic surveillance**

  * Colonoscopy at 1 year after surgery, then at 3 years, and subsequently every 5 years if no advanced lesions are detected.

5. **Lifestyle and survivorship care**

  * Counseling on physical activity, diet, weight control, smoking cessation, and management of treatment-related long-term effects.

Key principle

In the absence of symptoms or evidence of disease, **no additional oncologic therapy or invasive investigations are indicated**. Surveillance aims to detect potentially curable recurrence or metachronous neoplasia while avoiding unnecessary interventions.

If you want, I can adapt the answer to a **multiple-choice format**, **ESGE/ESMO wording**, or a **short exam-style response**.

Case 1.2

Vignette
A 70-year-old patient has been diagnosed with colorectal cancer and is scheduled for surgical treatment. A colonoscopy was performed several months ago, but its quality was suboptimal and the examination was incomplete.

Question
What would be the most appropriate management approach in this situation?

Most appropriate management: complete colonic evaluation before or around surgery

In a 70-year-old patient with **colorectal cancer scheduled for surgical treatment**, a prior **incomplete and suboptimal colonoscopy** is **not adequate for staging or surgical planning**. The correct approach is to ensure a **complete evaluation of the entire colon** to exclude synchronous neoplasia.

Recommended management

* **Repeat a high-quality colonoscopy** before surgery, if feasible, with adequate bowel preparation and complete cecal intubation.

* If repeat colonoscopy **cannot be performed or remains incomplete**, perform an **alternative complete colonic assessment**, such as:

  * **CT colonography**, or

  * **Intraoperative colonoscopy**, depending on local expertise and timing.

Rationale

* Synchronous colorectal cancers or advanced adenomas occur in a non-negligible proportion of patients and may change the **extent of surgical resection**.

* Relying on an incomplete examination risks **missing clinically relevant proximal lesions**.

Key principle

Definitive surgical treatment for colorectal cancer should be preceded by a **complete evaluation of the colon**, using colonoscopy or validated alternatives when colonoscopy is inadequate.

If you wish, I can condense this into a **single-sentence exam answer** or align it precisely with **ESGE/ESMO/NCCN wording**.

Case 1.3

Vignette
A 66-year-old patient underwent curative surgery for colorectal cancer one year ago. Recovery was uncomplicated, and no symptoms or abnormal findings have been reported during follow-up.

Question
What would be the most appropriate management approach in this situation?

Most appropriate management: scheduled surveillance colonoscopy as part of follow-up

In a 66-year-old patient **one year after curative surgery for colorectal cancer**, with an **uncomplicated course and no evidence of recurrence**, the appropriate management is to proceed with **routine post-operative surveillance**, with particular emphasis on **colonic evaluation at 1 year**.

Recommended approach

* **Perform surveillance colonoscopy at 1 year after surgery** (or 1 year after perioperative clearing colonoscopy, if performed), to:

  * Detect **metachronous colorectal cancer**

  * Identify and remove **advanced adenomas**

* Continue **standard oncologic surveillance**, including:

  * Periodic clinical assessment

  * CEA monitoring, if indicated

  * Scheduled imaging according to recurrence risk

Key principle

At 1 year after curative colorectal cancer surgery, **surveillance colonoscopy is mandatory**, even in asymptomatic patients, and no additional treatment or diagnostic escalation is indicated in the absence of abnormal findings.

If you want, I can rephrase this as a **very short board-style answer** or adapt it to a **multiple-choice explanation**.

Case 1.4

Vignette
A 72-year-old patient underwent curative colorectal cancer surgery three years ago. A surveillance colonoscopy performed one year after surgery was normal. The patient wonders whether repeating colonoscopy every year would improve long-term outcomes.

Question
What would be the most appropriate management approach in this situation?

Most appropriate management: extend the surveillance interval and avoid annual colonoscopy

In a 72-year-old patient **three years after curative colorectal cancer surgery**, with a **normal surveillance colonoscopy at 1 year**, repeating colonoscopy **every year is not indicated** and does not improve long-term outcomes.

Recommended approach

* **Schedule the next surveillance colonoscopy at 3 years after the normal 1-year examination**.

* If that examination is also normal, continue with **5-yearly colonoscopic surveillance thereafter**.

* Continue **standard clinical, biochemical, and radiological follow-up** according to oncologic guidelines.

Rationale

* Evidence shows that **annual colonoscopy does not reduce recurrence or mortality** in patients with a normal 1-year post-operative examination.

* More frequent colonoscopy increases **procedural risks, costs, and burden** without clear benefit.

Key principle

After a normal 1-year surveillance colonoscopy following curative colorectal cancer surgery, **surveillance intervals should be progressively extended**, and **annual colonoscopy should be avoided** in asymptomatic patients.

If you want, I can compress this into a **single-sentence exam answer** or align it exactly with **ESGE/ESMO/NCCN phrasing**.

Case 1.5

Vignette
A 69-year-old patient underwent curative surgery for colorectal cancer. A surveillance colonoscopy performed one year after surgery did not detect any neoplastic lesions, and no new symptoms have developed since then.

Question
What would be the most appropriate management approach in this situation?

Most appropriate management: continue guideline-based surveillance without intensification

In a 69-year-old patient who has undergone **curative colorectal cancer surgery**, with a **normal surveillance colonoscopy at 1 year** and **no new symptoms**, the appropriate approach is to **continue standard follow-up**, without increasing the frequency of colonoscopic examinations.

Recommended approach

* **Schedule the next surveillance colonoscopy at 3 years** after the 1-year examination.

* If that colonoscopy is also normal, proceed with **subsequent colonoscopies every 5 years**.

* Maintain **routine oncologic surveillance** with:

  * Periodic clinical assessment

  * CEA monitoring, if indicated

  * Imaging according to recurrence risk and guideline recommendations

Key principle

In asymptomatic patients with a **negative 1-year post-operative colonoscopy**, more intensive surveillance does **not** improve outcomes; adherence to **standard surveillance intervals** optimizes benefit while minimizing unnecessary procedures and risks.

If you need, I can adapt this to a **very concise exam-style answer** or harmonize the wording with **specific international guidelines**.

Case 1.6

Vignette
An 82-year-old patient underwent curative colorectal cancer surgery several years ago. Previous surveillance colonoscopies have been unremarkable. The patient has multiple chronic comorbidities that significantly limit overall life expectancy.

Question
What would be the most appropriate management approach in this situation?

Most appropriate management: discontinue routine colonoscopic surveillance and focus on individualized care

In an 82-year-old patient who underwent **curative colorectal cancer surgery several years ago**, with **repeated normal surveillance colonoscopies** and **significant comorbidities limiting life expectancy**, the most appropriate approach is to **stop routine surveillance colonoscopy**.

Recommended approach

* **Discontinue further scheduled colonoscopies**, as the likelihood of benefit is low and the procedural risks are relatively high.

* Shift follow-up to a **symptom-driven, patient-centered strategy**, with:

  * Clinical assessment as needed

  * Investigation only if new, relevant symptoms arise

* Focus on **quality of life, management of comorbidities, and supportive care**.

Rationale

* The preventive benefit of surveillance colonoscopy requires a sufficiently long life expectancy, which is unlikely in this context.

* In older, frail patients, colonoscopy carries increased risks and burden without meaningful impact on survival.

Key principle

Surveillance after colorectal cancer should be **individualized**; in patients with **limited life expectancy and repeated negative examinations**, it is appropriate to **de-escalate or stop colonoscopic surveillance**.

If you want, I can shorten this to a **single-sentence exam answer** or tailor it to **geriatric-oncology guideline language**.

Case 1.7

Vignette
A 68-year-old patient had a low-risk pT1 colorectal cancer completely removed by endoscopic resection with clear margins. No additional high-risk pathological features were reported, and the patient is currently asymptomatic.

Question
What would be the most appropriate management approach in this situation?

Most appropriate management: endoscopic surveillance without additional surgery

In a 68-year-old patient with a **low-risk pT1 colorectal cancer** that has been **completely removed endoscopically with clear (R0) margins** and **no high-risk pathological features**, the appropriate management is **endoscopic surveillance alone**, without surgical resection.

Recommended approach

* **No additional surgery** is indicated.
* Initiate a **structured endoscopic surveillance program**, typically:

  * First surveillance colonoscopy at **6 to 12 months** after endoscopic resection
  * Subsequent surveillance according to findings and standard post-polypectomy guidelines
* Continue **clinical follow-up**, with investigations driven by symptoms or endoscopic findings.

Rationale

* Low-risk pT1 cancers have a **very low risk of lymph node metastasis**, making the risks of surgery outweigh the potential benefits.
* Endoscopic resection with clear margins is considered **curative** in this setting.

Key principle

For low-risk pT1 colorectal cancer treated with **curative endoscopic resection**, **surveillance rather than surgery** is the standard of care.

If you wish, I can adapt this to a **one-line exam answer**, add a **brief definition of “low-risk pT1”**, or align it precisely with **ESGE/ESMO recommendations**.

Guideline 2. Post-polypectomy colonoscopy surveillance

Case 2.1

Vignette
A 58-year-old patient undergoes a high-quality colonoscopy with complete removal of three tubular adenomas, each measuring 6 mm, all showing low grade dysplasia. No additional lesions are detected, and the examination is considered complete and adequate.

Question
What would be the most appropriate follow-up strategy in this situation?

Most appropriate management: return to screening after low-risk adenoma removal

In a 58-year-old patient who has undergone a **high-quality, complete colonoscopy** with **complete removal of three small (6 mm) tubular adenomas with low grade dysplasia**, and **no additional lesions detected**, the appropriate strategy is **no endoscopic surveillance**.

A guideline-concordant approach includes:

1. **Endoscopic follow-up**
   - **No surveillance colonoscopy is indicated**.
   - The patient should be **returned to the routine colorectal cancer screening program**.
2. **Alternative strategy if organized screening is unavailable**
   - A **repeat colonoscopy at 10 years** after the index examination is appropriate.
3. **Patient counseling**
   - Reassure the patient that the findings are consistent with a **low-risk adenoma profile**.
   - Emphasize the importance of adherence to population-based screening and general preventive measures.

Key principle
After complete removal of **1–4 non-advanced adenomas <10 mm with low grade dysplasia**, a high-quality baseline colonoscopy is considered sufficient, and **surveillance colonoscopy does not provide additional benefit** while increasing the risk of overuse.

If you wish, I can reformat this as a **short exam answer**, **multiple-choice explanation**, or **explicit ESGE-aligned wording**.

Case 2.2

Vignette
A 60-year-old patient undergoes colonoscopy with complete removal of two small adenomas with low grade dysplasia. The patient lives in an area where no organized colorectal cancer screening program is available.

Question
How should follow-up be managed after this procedure?

Most appropriate management: long-interval endoscopic follow-up in the absence of organized screening

In a 60-year-old patient who has undergone **complete removal of two small adenomas with low grade dysplasia** at a **high-quality colonoscopy**, the findings are consistent with a **low-risk adenoma profile**.

In this setting, where **no organized colorectal cancer screening program is available**, the appropriate follow-up strategy is:

1. **Endoscopic follow-up**
   - **Repeat colonoscopy after 10 years** from the index examination.
2. **Rationale**
   - Patients with **1–4 non-advanced adenomas <10 mm with low grade dysplasia** do not benefit from earlier surveillance.
   - In the absence of population-based screening, a **10-year colonoscopic interval** substitutes for return to screening.
3. **Patient counseling**
   - Reassure the patient regarding the **very low risk of metachronous advanced neoplasia**.
   - Emphasize general preventive measures and prompt evaluation if new gastrointestinal symptoms develop.

Key principle
For patients with **low-risk adenomas**, follow-up is determined by the **availability of organized screening**: return to screening when available, or **10-year colonoscopy** when it is not.

Case 2.3

Vignette
A 63-year-old patient undergoes colonoscopy with complete removal of a 15-mm adenoma showing high grade dysplasia. The procedure is technically complete and of adequate quality.

Question
What would be the most appropriate surveillance approach for this patient?

Most appropriate management: early surveillance after removal of an advanced adenoma

In a 63-year-old patient who has undergone a **high-quality, complete colonoscopy** with **complete removal of a 15-mm adenoma showing high grade dysplasia**, the findings are consistent with an **advanced adenoma**, which confers an increased risk of metachronous advanced neoplasia.

A guideline-concordant surveillance strategy includes:

1. **Endoscopic surveillance**
   - **Surveillance colonoscopy at 3 years** after the index examination.
2. **Rationale**
   - Adenomas **≥10 mm** and or those with **high grade dysplasia** are classified as advanced lesions.
   - A **3-year interval** balances early detection of metachronous advanced neoplasia with avoidance of unnecessary overuse.
3. **Subsequent surveillance**
   - If the 3-year surveillance colonoscopy does not detect lesions requiring surveillance, further follow-up can be **extended according to subsequent findings**, typically at longer intervals.
4. **Patient counseling**
   - Inform the patient about the **higher baseline risk** compared with low-risk adenomas.
   - Reinforce the importance of adherence to the recommended surveillance interval.

Key principle
After complete removal of an **advanced adenoma**, **3-year colonoscopic surveillance** is indicated, even when the baseline examination is of high quality and complete.

Case 2.4

Vignette
A 67-year-old patient undergoes piecemeal endoscopic resection of a 30-mm nonpedunculated colorectal polyp. The resection is technically successful, with no immediate complications.

Question
How should follow-up be managed after this resection?

Most appropriate management: early repeat colonoscopy after piecemeal resection of a large colorectal polyp

In a 67-year-old patient who has undergone **piecemeal endoscopic resection of a 30-mm nonpedunculated colorectal polyp**, the risk of **residual or early recurrent neoplasia** is significant despite technical success.

A guideline-concordant follow-up strategy includes:

1. **Early repeat colonoscopy**
   - **Repeat colonoscopy at 3 to 6 months** after the index resection to assess for residual or recurrent tissue at the resection site.
2. **First surveillance after confirmation of clearance**
   - If no residual lesion is detected at the early repeat colonoscopy, a **first surveillance colonoscopy at 12 months** is recommended to detect late recurrence.
3. **Subsequent surveillance**
   - If the 12-month surveillance colonoscopy is negative for lesions requiring surveillance, a **second surveillance colonoscopy after 5 years** is appropriate.
4. **Rationale**
   - Piecemeal resection of lesions **≥20 mm** carries a higher recurrence risk than en bloc resection.
   - Early site inspection and structured subsequent surveillance reduce the risk of missed residual disease.
5. **Patient counseling**
   - Explain the need for **closer follow-up** compared with en bloc resection or smaller lesions.
   - Emphasize adherence to the surveillance schedule.

Key principle
After piecemeal resection of a **large (≥20 mm) colorectal polyp**, surveillance should be **intensified and stepwise**, starting with an **early 3 to 6-month repeat colonoscopy**, followed by structured longer-term surveillance if clearance is confirmed.

Case 2.5

Vignette
A patient previously treated with piecemeal endoscopic resection of a large colorectal polyp undergoes an early repeat colonoscopy, which shows no residual lesion.

Question
What further surveillance strategy would be appropriate after this examination?

Most appropriate management: structured surveillance after negative early repeat colonoscopy

In a patient who previously underwent **piecemeal endoscopic resection of a large colorectal polyp** and whose **early repeat colonoscopy shows no residual or recurrent lesion**, follow-up should continue with a **stepwise surveillance strategy**.

A guideline-concordant approach includes:

1. **First surveillance colonoscopy**
    - **Surveillance colonoscopy at 12 months** after the early repeat examination to detect late recurrence.
2. **Second surveillance colonoscopy**
    - If the 12-month surveillance colonoscopy does not identify lesions requiring surveillance, a **second surveillance colonoscopy after 5 years** is appropriate.
3. **Rationale**
    - Even after a negative early repeat examination, **late recurrence can occur** following piecemeal resection of large lesions.
    - A structured, time-based approach ensures detection while avoiding unnecessary overuse.
4. **Patient counseling**
    - Reassure the patient regarding the **favorable early outcome**, while explaining the need for continued surveillance.
    - Emphasize the importance of adherence to the recommended intervals.

Key principle
After piecemeal resection of a large colorectal polyp, a **negative early repeat colonoscopy does not end surveillance**. Ongoing follow-up with a **12-month colonoscopy**, followed by **5-year surveillance if negative**, is required to ensure long-term safety.

Case 2.6

Vignette
A patient undergoes a first surveillance colonoscopy after prior polypectomy. The examination shows no polyps requiring further surveillance.

Question
How should subsequent follow-up be planned in this situation?

Most appropriate management: extended-interval surveillance after a negative first surveillance colonoscopy

In a patient who has undergone **prior polypectomy** and whose **first surveillance colonoscopy does not identify any polyps requiring further surveillance**, the appropriate approach is to **extend the surveillance interval**.

A guideline-concordant follow-up strategy includes:

1. **Subsequent endoscopic surveillance**
   - **Second surveillance colonoscopy after 5 years**.
2. **Rationale**
   - A negative first surveillance colonoscopy indicates a **low risk of metachronous advanced neoplasia**.
   - Extending the interval reduces unnecessary procedures without compromising safety.
3. **Further follow-up**
   - Subsequent surveillance should be **individualized based on findings** at the second surveillance colonoscopy and overall patient risk profile.
4. **Patient counseling**
   - Reassure the patient regarding the **favorable findings**.
   - Emphasize continued adherence to the recommended surveillance schedule.

Key principle
After polypectomy, a **negative first surveillance colonoscopy** allows for **lengthening of surveillance intervals**, with a **5-year interval** being appropriate in the absence of lesions requiring further follow-up.

Case 2.7

Vignette
During a surveillance colonoscopy following previous polypectomy, new adenomas meeting criteria for further surveillance are detected and completely removed.

Question
What would be the most appropriate next step in follow-up?

Most appropriate management: reset surveillance according to newly detected lesions

In a patient undergoing **surveillance colonoscopy after prior polypectomy**, in whom **new adenomas meeting criteria for further surveillance are detected and completely removed**, follow-up should be **redefined based on the characteristics of the newly identified lesions**, not on the previous surveillance history.

A guideline-concordant approach includes:

1. **Reclassification of risk**
   - The patient should be **reclassified according to the number, size, histology, and dysplasia grade** of the newly detected adenomas.
2. **Endoscopic follow-up**
   - The **surveillance interval should be reset**, starting from the current colonoscopy.
   - The timing of the next colonoscopy should follow the **standard surveillance recommendations applicable to the newly detected lesions**:
     - Low-risk adenomas: return to screening or long-interval follow-up, depending on screening availability.
     - Advanced adenomas or multiple adenomas: **shorter surveillance intervals**, typically **3 years**, according to lesion risk.
3. **Rationale**
   - The risk of future neoplasia is driven by the **most recent and highest-risk findings**.
   - Continuing an interval based on previous negative examinations would underestimate current risk.
4. **Patient counseling**
   - Explain that the detection of new adenomas changes the **risk profile and follow-up plan**.
   - Emphasize adherence to the newly recommended surveillance interval.

Key principle
When adenomas requiring surveillance are found at a surveillance colonoscopy, **follow-up must be recalculated from that examination**, applying the **same rules used after index polypectomy**, based on the **worst newly detected lesion**.

Case 2.8

Vignette
An 82-year-old patient with a history of adenoma removal has undergone several surveillance colonoscopies. The patient has multiple chronic comorbidities that significantly limit overall life expectancy.

Question
How should post-polypectomy surveillance be managed at this stage?

Most appropriate management: individualized, life expectancy–based cessation of surveillance

In an 82-year-old patient with a **history of adenoma removal** who has already undergone **multiple surveillance colonoscopies**, the presence of **significant comorbidities limiting life expectancy** shifts the balance of benefit and harm.

A guideline-consistent, patient-centered approach includes:

1. **Reassessment of benefit vs risk**
    - The **expected benefit of continued surveillance is low**, given limited life expectancy and the typically slow progression of colorectal neoplasia.
    - The **procedural risks** of colonoscopy increase with age and comorbidity.
2. **Endoscopic surveillance**
    - **Discontinuation of routine post-polypectomy surveillance** is appropriate.
    - No further scheduled colonoscopies should be planned.
3. **Alternative approach**
    - Adopt a **symptom-driven strategy**, with investigations reserved for **new, clinically significant symptoms**such as overt gastrointestinal bleeding, iron deficiency anemia, or unexplained weight loss.
4. **Shared decision-making**
    - Discuss goals of care, patient preferences, and overall prognosis.
    - Document the rationale for stopping surveillance in the medical record.

Key principle
Post-polypectomy surveillance should be **individualized** and **may be discontinued** when **life expectancy is limited**, as continued colonoscopy is unlikely to provide meaningful clinical benefit and may expose the patient to avoidable harm.

Guideline 3. Endoscopic management of Lynch syndrome and of familial risk of colorectal cancer

Case 3.1

Vignette
An individual with a confirmed diagnosis of Lynch syndrome is followed at a local healthcare facility where colonoscopies are performed intermittently by different endoscopists, without a structured system to monitor adherence to surveillance schedules or procedural quality indicators.

Question
What would be the most appropriate approach to managing follow-up care in this situation?

Most appropriate management: centralized, structured surveillance in an expert setting

In an individual with a **confirmed diagnosis of Lynch syndrome**, appropriate follow-up requires **high-intensity, high-quality surveillance**. Intermittent colonoscopies performed by different endoscopists **without monitoring of timing or quality indicators** are inadequate and potentially unsafe in this high-risk population.

A guideline-concordant approach includes:

1. **Centralization of care**
    - The patient should be referred to a **dedicated center with expertise in hereditary colorectal cancer syndromes**.
    - Surveillance should be coordinated by a **multidisciplinary team** experienced in Lynch syndrome management.
2. **Structured endoscopic surveillance**
    - Colonoscopies should be performed at **regular, predefined intervals** according to Lynch syndrome recommendations, typically **every 1 to 2 years**, starting at the appropriate age.
    - Surveillance should be performed by **experienced endoscopists**, ideally the same team over time, to ensure consistency.
3. **Quality assurance**
    - Colonoscopies must meet **strict quality standards**, including adequate bowel preparation, complete examination, sufficient withdrawal time, and meticulous mucosal inspection.
    - Adherence to surveillance intervals and quality indicators should be **actively monitored and documented**.
4. **Continuity and coordination of care**
    - A **registry-based or recall system** should be in place to ensure adherence to surveillance schedules.
    - Coordination with **genetic counseling**, extracolonic cancer surveillance, and family cascade testing is essential.
5. **Patient counseling**
    - Explain the **high lifetime risk of colorectal cancer** and the rationale for intensive, structured surveillance.

- Emphasize that surveillance effectiveness in Lynch syndrome depends not only on frequency but also on **procedural quality and continuity**.

Key principle
In Lynch syndrome, follow-up should not be opportunistic or fragmented. **Centralized, protocol-driven surveillance in experienced centers**, with active monitoring of timing and quality, is essential to effectively reduce colorectal cancer incidence and mortality.

Case 3.2

Vignette
A 26-year-old asymptomatic individual is known to carry a pathogenic MLH1 mutation and has never undergone colonoscopic evaluation.

Question
What would be the most appropriate approach to colorectal surveillance at this stage?

Most appropriate management: initiation of intensive colonoscopic surveillance for Lynch syndrome

In a 26-year-old asymptomatic individual with a **confirmed pathogenic MLH1 mutation**, the risk of early-onset colorectal cancer is substantially increased, even in the absence of symptoms. Surveillance should therefore be **initiated without delay**.

A guideline-concordant approach includes:

1. **Initiation of colonoscopic surveillance**
   - **Immediate baseline colonoscopy** is indicated.
   - Surveillance should be performed by an **experienced endoscopist** using a high-quality technique.
2. **Surveillance interval**
   - **Colonoscopy every 1 to 2 years** thereafter.
   - Given the higher colorectal cancer risk associated with **MLH1 mutations**, a **1-year interval** is often preferred, particularly after the first examination.
3. **Quality requirements**
   - High-quality bowel preparation and complete examination to the cecum.
   - Careful inspection, adequate withdrawal time, and complete removal of all detected lesions.
4. **Continuity of care**
   - Surveillance should ideally be coordinated within a **specialized hereditary cancer or Lynch syndrome program**, with a recall system to ensure adherence.
5. **Patient counseling**
   - Explain that surveillance is recommended **regardless of symptoms**, as colorectal cancer in Lynch syndrome may develop rapidly.
   - Discuss the importance of long-term adherence to the surveillance program.

Key principle
In carriers of a **pathogenic MLH1 mutation**, **colonoscopy should start in early adulthood** and be repeated at **short intervals**, as early and regular surveillance is the most effective strategy to prevent colorectal cancer–related morbidity and mortality.

Case 3.3

Vignette
A 38-year-old asymptomatic individual with a confirmed diagnosis of Lynch syndrome is enrolled in a surveillance program and has been undergoing regular follow-up.

Question
How should colonoscopic surveillance be scheduled for this individual?

Most appropriate management: continued high-intensity colonoscopic surveillance in Lynch syndrome

In a 38-year-old asymptomatic individual with a **confirmed diagnosis of Lynch syndrome** who is already enrolled in a surveillance program, colonoscopic follow-up should remain **regular, intensive, and structured**, irrespective of the absence of symptoms.

A guideline-concordant approach includes:

1. **Surveillance interval**
   - **Colonoscopy every 1 to 2 years**.
   - In individuals with **MLH1 or MSH2 mutations**, a **1-year interval** is generally preferred due to higher colorectal cancer risk.
   - For **MSH6 or PMS2 mutations**, a **1–2-year interval** may be acceptable, depending on individual risk factors and prior findings.
2. **Quality requirements**
   - Surveillance colonoscopies should be performed by **experienced endoscopists** with expertise in hereditary cancer syndromes.
   - High-quality bowel preparation, complete examination, and meticulous mucosal inspection are essential.
3. **Adjustment based on findings**
   - Detection of adenomas or other neoplastic lesions may warrant **shortening of the interval**, according to lesion characteristics.
   - A normal examination does **not justify extending intervals beyond 2 years**.
4. **Programmatic follow-up**
   - Surveillance should be embedded in a **structured program** with a recall system to ensure adherence over time.
5. **Patient counseling**
   - Reinforce that the goal of frequent surveillance is **prevention and early detection**, not symptom-driven diagnosis.
   - Emphasize the lifelong nature of surveillance in Lynch syndrome.

Key principle
In Lynch syndrome, colonoscopic surveillance is **lifelong and time-driven**, not symptom-driven. Regular **1- to 2-year colonoscopy** remains the cornerstone of colorectal cancer prevention, even during long-term follow-up.

Case 3.4

Vignette
An individual with Lynch syndrome undergoes a surveillance colonoscopy that is incomplete because of poor bowel preparation.

Question
How should follow-up be managed after this examination?

Most appropriate management: prompt repeat colonoscopy to ensure adequate surveillance

In an individual with **Lynch syndrome**, an **incomplete surveillance colonoscopy due to poor bowel preparation** is **not an acceptable surveillance examination** and does not provide adequate cancer prevention.

A guideline-concordant approach includes:

1. **Repeat colonoscopy**
   - The colonoscopy should be **repeated as soon as feasible**, typically **within a short interval**, after optimization of bowel preparation.
   - The examination should be considered a **replacement**, not a continuation, of the failed procedure.
2. **Optimization of bowel preparation**
   - Use an **enhanced bowel preparation regimen**, potentially including split dosing, intensified instructions, or adjunctive measures.
   - Identify and address factors associated with inadequate preparation, such as constipation, comorbidities, or medication use.
3. **Quality requirements**
   - The repeat examination should be performed by an **experienced endoscopist**, ideally within a specialized Lynch syndrome surveillance program.
   - Complete examination to the cecum with careful mucosal inspection is mandatory.
4. **Surveillance scheduling**
   - The **surveillance clock should restart from the date of the adequate colonoscopy**, not from the incomplete examination.
5. **Patient counseling**
   - Explain that poor bowel preparation compromises cancer prevention in Lynch syndrome.
   - Emphasize the importance of adherence to preparation instructions.

Key principle
In Lynch syndrome, an incomplete colonoscopy is **equivalent to no surveillance**. **Prompt repeat colonoscopy with optimized preparation** is required, and future surveillance intervals should be based only on **adequate, complete examinations**.

Case 3.5

Vignette
An individual with Lynch syndrome who is enrolled in a surveillance program develops new gastrointestinal symptoms between scheduled examinations.

Question
What would be the most appropriate next step in management?

Most appropriate management: symptom-driven diagnostic colonoscopy outside the routine surveillance schedule

In an individual with **Lynch syndrome** who develops **new gastrointestinal symptoms** while enrolled in a regular surveillance program, management should shift from purely time-based follow-up to **prompt diagnostic evaluation**.

A guideline-concordant approach includes:

1. **Immediate clinical assessment**
   - Careful evaluation of the type, severity, and duration of symptoms such as rectal bleeding, change in bowel habits, iron-deficiency anemia, unexplained abdominal pain, or weight loss.
2. **Prompt diagnostic colonoscopy**
   - **Colonoscopy should be performed as soon as feasible**, regardless of the timing of the last surveillance examination.
   - The procedure should be conducted with **high-quality standards** by an experienced endoscopist.
3. **Further investigations as indicated**
   - Additional tests, including laboratory studies or cross-sectional imaging, may be warranted depending on symptoms and colonoscopic findings.
4. **Re-definition of surveillance schedule**
   - After diagnostic evaluation and management of any findings, the **routine surveillance schedule should be resumed or adjusted** based on the most recent and highest-risk findings.
5. **Patient counseling**
   - Emphasize that, in Lynch syndrome, **symptoms must never be attributed to interval surveillance alone**.
   - Reinforce the importance of **early reporting of new symptoms**.

Key principle
In Lynch syndrome, surveillance is **preventive**, not diagnostic. The development of **new gastrointestinal symptoms mandates immediate diagnostic colonoscopy**, independent of the planned surveillance interval.

Case 3.6

Vignette
A patient with Lynch syndrome undergoes colonoscopic surveillance using a standard-definition endoscopy system.

Question
What considerations should guide the choice of endoscopic equipment for future surveillance?

Most appropriate management: optimize endoscopic technology to maximize lesion detection

In a patient with **Lynch syndrome**, colonoscopic surveillance aims to detect **flat, subtle, and rapidly progressive lesions**. The use of **standard-definition endoscopy** may be suboptimal in this high-risk setting.

Key considerations for future surveillance include:

1. **Use of high-definition endoscopy**
   - **High-definition colonoscopy** should be the standard for surveillance in Lynch syndrome.
   - Improved image resolution enhances detection of **flat and nonpolypoid lesions**, which are more common in this population.
2. **Advanced imaging techniques**
   - Routine use of **image-enhanced endoscopy** such as virtual chromoendoscopy is recommended to improve mucosal visualization.
   - These techniques facilitate detection of subtle lesions that may be missed with white-light standard-definition systems.
3. **Endoscopist expertise over equipment alone**
   - While advanced equipment is important, **experienced endoscopists** with expertise in hereditary cancer surveillance remain essential.
   - Adequate withdrawal time and meticulous inspection are mandatory.
4. **Programmatic consistency**
   - Surveillance should ideally be performed using **consistent, high-quality equipment** within a structured program to ensure reproducibility over time.
5. **Patient counseling**
   - Explain that optimized technology improves preventive effectiveness without increasing procedural risk.

Key principle
In Lynch syndrome, surveillance quality is critical. **High-definition endoscopy with image enhancement**, performed by experienced endoscopists, should be preferred over standard-definition systems to maximize colorectal cancer prevention.

Case 3.7

Vignette
An asymptomatic individual with Lynch syndrome asks whether the use of dye-based techniques during colonoscopy could improve lesion detection.

Question
How should this option be considered in planning surveillance?

Most appropriate management: selective use of dye-based chromoendoscopy within high-quality surveillance

In an asymptomatic individual with **Lynch syndrome**, maximizing lesion detection is a key objective of colonoscopic surveillance. **Dye-based chromoendoscopy** can enhance mucosal visualization, but its role should be considered in the context of overall surveillance quality.

A guideline-consistent approach includes:

1. **Role of dye-based chromoendoscopy**
    - Dye-based chromoendoscopy may **increase detection of flat and subtle lesions**, which are more frequent in Lynch syndrome.
    - It can be considered as an **adjunct technique**, particularly in patients with prior neoplasia or suboptimal visualization with standard white-light endoscopy.
2. **Comparison with virtual chromoendoscopy**
    - Modern **high-definition virtual chromoendoscopy** provides comparable detection performance in many settings.
    - When high-definition systems with image enhancement are available, routine dye spraying is **not mandatory**.
3. **Practical considerations**
    - Dye-based techniques require **additional time, expertise, and resources**.
    - Their benefit is maximized in **experienced hands** within structured surveillance programs.
4. **Priority of overall quality**
    - Adequate bowel preparation, complete examination, sufficient withdrawal time, and careful inspection are **more important than the choice of dye alone**.
5. **Patient counseling**
    - Explain that dye-based chromoendoscopy is **one of several tools** to optimize detection.
    - Reassure the patient that high-quality surveillance can be achieved **with or without dye**, depending on available technology and expertise.

Key principle
In Lynch syndrome, the cornerstone of surveillance is **high-quality colonoscopy**. **Dye-based chromoendoscopy may be considered selectively**, but it should complement, not replace, optimized technique, advanced imaging, and endoscopist expertise.

Case 3.8

Vignette
An asymptomatic patient with Lynch syndrome asks whether regular upper gastrointestinal endoscopic examinations are necessary as part of routine follow-up.

Question
How should this issue be addressed?

Most appropriate management: individualized upper gastrointestinal surveillance based on risk profile

In an asymptomatic patient with **Lynch syndrome**, the need for **upper gastrointestinal (UGI) endoscopic surveillance**should be addressed in a **risk-stratified and individualized manner**, rather than applied universally.

A guideline-consistent approach includes:

1. **Assessment of individual risk**
    - UGI cancer risk in Lynch syndrome varies by **gene involved** (higher in MLH1 and MSH2), **family history** of gastric or duodenal cancer, **ethnic background**, and **geographic incidence**.
    - The presence of **Helicobacter pylori infection** is an additional modifiable risk factor.
2. **Upper gastrointestinal endoscopy**
    - Routine UGI endoscopy is **not mandatory for all Lynch syndrome carriers**.
    - It may be **considered** in individuals at higher risk, typically starting in adulthood, with **periodic esophagogastroduodenoscopy** at defined intervals.
3. **H. pylori testing and management**
    - **Testing for H. pylori** is recommended in all Lynch syndrome patients.
    - **Eradication therapy** should be offered if infection is detected, regardless of endoscopic surveillance plans.
4. **Symptom-driven evaluation**
    - Upper GI endoscopy should be **promptly performed** if alarm symptoms develop, including dyspepsia with anemia, weight loss, vomiting, or gastrointestinal bleeding.
5. **Patient counseling**
    - Explain that, unlike colorectal cancer, the benefit of routine UGI surveillance is **less clearly established**.
    - Decisions should be made through **shared decision-making**, balancing potential benefits, burdens, and patient preferences.

Key principle
In Lynch syndrome, **colorectal surveillance is mandatory**, whereas **upper gastrointestinal surveillance is optional and risk-adapted**. A personalized approach, combined with **H. pylori detection and eradication**, provides the most balanced strategy for follow-up.

Case 3.9

Vignette
An individual with Lynch syndrome asks whether testing for *Helicobacter pylori* should be performed despite the absence of gastrointestinal symptoms.

Question
What would be the most appropriate advice in this situation?

Most appropriate management: proactive testing and eradication of *Helicobacter pylori*

In an individual with **Lynch syndrome**, testing for *Helicobacter pylori* is appropriate **even in the absence of gastrointestinal symptoms**, as part of a preventive, risk-reduction strategy.

A guideline-consistent approach includes:

1. **H. pylori testing**
   - *H. pylori* testing should be **offered to all individuals with Lynch syndrome**, regardless of symptoms.
   - Noninvasive tests such as the **urea breath test** or **stool antigen test** are appropriate first-line options.
2. **Management of positive results**
   - **Eradication therapy** should be initiated if infection is detected.
   - Confirmation of eradication is recommended after completion of treatment.
3. **Rationale**
   - *H. pylori* is a recognized risk factor for **gastric cancer**, which occurs more frequently in Lynch syndrome than in the general population.
   - Eradication represents a **modifiable risk factor** and a low-burden preventive intervention.
4. **Relation to upper GI surveillance**
   - *H. pylori* testing and eradication are recommended **independently of decisions regarding routine upper GI endoscopy**.
5. **Patient counseling**
   - Explain that testing is recommended for **cancer prevention**, not symptom evaluation.
   - Reassure the patient about the safety and effectiveness of eradication therapy.

Key principle
In Lynch syndrome, *Helicobacter pylori* testing is a **preventive measure** and should be performed **regardless of symptoms**, with eradication offered when infection is identified.

Case 3.10

Vignette
An asymptomatic individual with Lynch syndrome asks whether additional investigations of the small bowel should be routinely included in surveillance.

Question
What would be the most appropriate management approach?

Most appropriate management: avoid routine small bowel surveillance, adopt a risk- and symptom-driven approach

In an asymptomatic individual with **Lynch syndrome**, the role of **routine small bowel investigation** as part of surveillance is limited and should not be applied universally.

A guideline-consistent approach includes:

1. **Routine surveillance**
   - **Routine small bowel investigations are not recommended** in asymptomatic individuals with Lynch syndrome.
   - Available evidence does not support a clear benefit in terms of cancer prevention or survival.
2. **Indications for small bowel evaluation**
   - Small bowel investigations, such as **capsule endoscopy or cross-sectional imaging**, should be **reserved for specific indications**, including:
     - Unexplained iron-deficiency anemia
     - Obscure gastrointestinal bleeding
     - Persistent abdominal symptoms not explained by colonoscopy or upper GI endoscopy
3. **Risk stratification**
   - Routine screening may be considered only in **exceptional high-risk scenarios**, such as a strong family history of small bowel cancer, but this is not standard practice.
4. **Patient counseling**
   - Explain that small bowel cancer in Lynch syndrome is **rare**, even though the relative risk is increased.
   - Emphasize that unnecessary investigations may lead to false positives and avoidable procedures.
5. **Focus on high-yield surveillance**
   - Reinforce that **high-quality colonoscopic surveillance** remains the cornerstone of cancer prevention in Lynch syndrome.

Key principle
In Lynch syndrome, surveillance should focus on **interventions with proven benefit**. **Routine small bowel investigations are not indicated** in asymptomatic individuals and should be reserved for **symptom-driven or selected high-risk situations**.

Case 3.11

Vignette
A 45-year-old individual reports a family history of colorectal cancer affecting two first-degree relatives, one of whom was diagnosed before the age of 50 years.

Question
How should colorectal cancer risk be classified in this case?

Most appropriate management: classification as increased familial colorectal cancer risk

In a 45-year-old individual with a **family history of colorectal cancer involving two first-degree relatives**, including **one diagnosed before the age of 50 years**, the colorectal cancer risk is **significantly higher than average**.

Risk classification includes:

1. **Risk category**
   - This pattern is consistent with **increased familial risk of colorectal cancer**.
   - It does **not automatically establish a diagnosis of Lynch syndrome**, but it exceeds average-risk criteria.
2. **Rationale**
   - Colorectal cancer in **two first-degree relatives**, particularly with **early-onset disease**, confers a substantially elevated lifetime risk.
   - This familial clustering suggests a possible **hereditary or shared environmental component**.
3. **Implications**
   - The individual should be managed according to **familial-risk colorectal cancer pathways**, which include **earlier and more intensive screening** than average-risk populations.
   - **Referral for genetic counseling** may be appropriate, especially if additional features suggest a hereditary syndrome.
4. **Patient counseling**
   - Explain that the risk is **higher than population risk**, but not necessarily due to a defined hereditary syndrome.
   - Discuss the need for **structured screening and follow-up**.

Key principle
A family history of colorectal cancer in **two first-degree relatives, one with early-onset disease**, defines an **increased familial risk category**, warranting enhanced surveillance compared with average-risk individuals.

Case 3.12

Vignette
A first-degree relative of a patient with colorectal cancer belongs to a family that meets criteria for increased familial risk.

Question
What would be the most appropriate colorectal surveillance strategy for this individual?

Most appropriate management: intensified colonoscopic surveillance for increased familial risk

In a **first-degree relative of a patient with colorectal cancer** belonging to a family that meets criteria for **increased familial colorectal cancer risk**, surveillance should be **earlier and more intensive** than in average-risk individuals.

A guideline-concordant strategy includes:

1. **Initiation of surveillance**
   - **Colonoscopy starting at age 40 years, or 10 years earlier than the youngest age at diagnosis in the family**, whichever comes first.
2. **Surveillance interval**
   - **Colonoscopy every 5 years**, provided examinations are of high quality and findings are normal.
   - Shorter intervals may be required if adenomas or advanced lesions are detected.
3. **Rationale**
   - First-degree relatives in families with increased familial risk have a **substantially elevated lifetime risk** of colorectal cancer.
   - Five-yearly colonoscopy balances effective cancer prevention with avoidance of unnecessary overuse.
4. **Quality requirements**
   - High-quality colonoscopy with adequate bowel preparation, complete examination, and careful mucosal inspection is essential.
5. **Patient counseling**
   - Explain that surveillance differs from population screening because of **familial risk**.
   - Reinforce the importance of long-term adherence to the surveillance program.
   - Consider **genetic counseling** if additional features suggest a hereditary syndrome.

Key principle
In individuals with **increased familial colorectal cancer risk**, surveillance should begin **earlier** and be performed at **5-year intervals**, with timing anchored to the **youngest affected family member**.

Case 3.13

Vignette
A 41-year-old individual with an established familial risk of colorectal cancer has not previously undergone colonoscopy.

Question
When should colorectal surveillance be initiated?

Most appropriate management: immediate initiation of colonoscopic surveillance

In a 41-year-old individual with an **established familial risk of colorectal cancer** who has **never undergone colonoscopy**, colorectal surveillance should be **initiated without further delay**.

A guideline-concordant approach includes:

1. **Timing of initiation**
   - **Immediate baseline colonoscopy** is indicated.
   - This applies because surveillance in familial-risk individuals should start at **age 40 years**, or **10 years earlier than the youngest affected relative**, whichever comes first.
2. **Subsequent surveillance**
   - If the baseline colonoscopy is normal and of high quality, **repeat colonoscopy every 5 years** is appropriate.
   - Surveillance intervals should be adjusted based on findings at baseline.
3. **Rationale**
   - Delaying surveillance beyond the recommended starting age increases the risk of **missed advanced neoplasia**.
   - Early initiation allows detection and removal of precursor lesions before malignant transformation.
4. **Patient counseling**
   - Explain the reason for starting surveillance now despite the absence of symptoms.
   - Emphasize the importance of adherence to long-term follow-up.

Key principle
In individuals with **familial colorectal cancer risk**, colonoscopic surveillance should begin **at the recommended starting age**, and a missed start warrants **immediate initiation** rather than further postponement.

Case 3.14

Vignette
An individual with familial risk of colorectal cancer undergoes a high-quality baseline colonoscopy that shows no neoplastic lesions.

Question
How should future surveillance be planned?

Most appropriate management: continued 5-yearly colonoscopic surveillance after a negative baseline examination

In an individual with **familial risk of colorectal cancer** who has undergone a **high-quality baseline colonoscopy with no neoplastic lesions detected**, follow-up should continue according to **familial-risk surveillance pathways**, not average-risk screening.

A guideline-concordant approach includes:

1. **Surveillance interval**
   - **Repeat colonoscopy every 5 years**.
2. **Rationale**
   - A negative baseline colonoscopy reduces short-term risk but does **not normalize lifetime risk** in individuals with familial predisposition.
   - Five-year intervals provide effective prevention while avoiding unnecessary procedures.
3. **Adjustment based on findings**
   - If adenomas or advanced lesions are detected at follow-up, surveillance intervals should be **shortened according to post-polypectomy recommendations**.
4. **Quality requirements**
   - Each surveillance colonoscopy should be of **high quality**, with adequate bowel preparation, complete examination, and meticulous mucosal inspection.
5. **Patient counseling**
   - Reassure the patient about the favorable baseline findings.
   - Emphasize that continued surveillance is required despite a normal first examination.

Key principle
In individuals with **familial colorectal cancer risk**, a negative baseline colonoscopy does **not terminate intensified surveillance**. **Five-yearly colonoscopy** remains appropriate to mitigate long-term risk.

Case 3.15

Vignette
A patient with familial risk of colorectal cancer undergoes colonoscopy with removal of one or more adenomas.

Question
How should follow-up after polyp removal be managed?

Most appropriate management: apply post-polypectomy surveillance rules based on polyp findings

In a patient with **familial risk of colorectal cancer** who undergoes colonoscopy with **removal of one or more adenomas**, follow-up should be determined by the **characteristics of the detected polyps**, not by familial risk alone.

A guideline-concordant approach includes:

1. **Risk reclassification after polypectomy**
    - The patient should be **reclassified according to the number, size, histology, and grade of dysplasia** of the removed adenomas.
2. **Endoscopic surveillance**
    - The **surveillance interval should follow standard post-polypectomy recommendations**, starting from the index colonoscopy at which adenomas were removed.
    - Examples include:
        - Low-risk adenomas: long-interval follow-up or return to screening, depending on local screening availability.
        - Advanced adenomas or multiple adenomas: **shorter surveillance intervals**, typically **3 years**, according to lesion risk.
3. **Interaction with familial risk**
    - Familial risk **does not shorten surveillance intervals beyond those dictated by polyp pathology**.
    - The **highest-risk feature** (polyp characteristics vs family history) should guide follow-up.
4. **Subsequent surveillance**
    - If surveillance colonoscopies are negative, intervals may be **extended according to guideline recommendations**.
5. **Patient counseling**
    - Explain that once adenomas are found, **polyp-related risk supersedes family history** in determining surveillance timing.
    - Emphasize adherence to the recommended follow-up plan.

Key principle
In individuals with familial colorectal cancer risk, the detection of adenomas shifts management to **standard post-polypectomy surveillance pathways**, with follow-up driven by the **most advanced lesion removed**, rather than family history alone.

Guideline 4. Colorectal polypectomy and endoscopic mucosal resection

Case 4.1

Vignette
During colonoscopy, a flat 25 mm nonpedunculated colorectal lesion is identified. The endoscopist evaluates the lesion using white-light endoscopy and virtual chromoendoscopy, assessing pit and vascular patterns together with macroscopic features.

Question
How should optical diagnosis be performed and integrated to estimate the risk of submucosal invasion in this lesion?

Most appropriate management: structured optical diagnosis integrating macroscopic and microvascular assessment

In a patient with a **flat, nonpedunculated 25 mm colorectal lesion**, optical diagnosis should be performed using a **systematic and integrative approach** that combines **macroscopic morphology**, **white-light endoscopy**, and **virtual chromoendoscopy**, with the specific aim of **estimating the risk of submucosal invasion** and guiding treatment strategy.

Recommended approach

1. **Macroscopic assessment**
   - Classify the lesion according to the **Paris classification**, with particular attention to features associated with deep submucosal invasion, such as non-granular laterally spreading tumors, focal depression, surface irregularity, ulceration, fold convergence, or stiffness.
2. **High-definition white-light endoscopy**
   - Evaluate overall surface architecture, color changes, and lesion margins, identifying any signs suggestive of invasive growth.
3. **Virtual chromoendoscopy**
   - Systematically assess **pit and vascular patterns** using validated classification systems (such as Kudo, NICE, or JNET).
   - Regular pit and vessel patterns are consistent with superficial neoplasia, whereas irregular or disrupted patterns suggest an increased risk of deep submucosal invasion.
4. **Integrated interpretation**
   - Optical findings should be interpreted **globally**, integrating macroscopic features with pit and vascular pattern analysis, rather than relying on a single optical criterion.
   - Lesions with optical features suggestive of deep submucosal invasion should be referred for **surgical management**, while lesions consistent with superficial invasion may be treated with **advanced endoscopic resection techniques**, preferably en bloc.

Key principle

Optical diagnosis of large, flat colorectal lesions must be **structured, validated, and integrative**, as accurate estimation of submucosal invasion risk is essential to select the appropriate therapeutic approach and avoid both undertreatment and unnecessary surgery.

Case 4.2

Vignette
A 15 mm laterally spreading colorectal lesion is detected during screening colonoscopy.

Question
How should this polyp be described and reported in the endoscopy report according to guideline recommendations?

Most appropriate management: standardized and complete endoscopic description of the lesion

In the case of a **15 mm laterally spreading colorectal lesion (LST)** detected during screening colonoscopy, the polyp should be **systematically described and reported using a standardized framework**, as recommended by international guidelines, to ensure appropriate risk stratification and management.

Recommended approach

1. **Morphological classification**
    - Describe the lesion as a **laterally spreading tumor (LST)** and specify the subtype:
        - **Granular** (homogeneous or mixed nodular), or
        - **Non-granular** (flat elevated or pseudodepressed).
    - Classify the lesion according to the **Paris classification**.
2. **Size and location**
    - Report the **maximum diameter** (15 mm) and the **exact colonic location**, including segment and distance from the anal verge when appropriate.
3. **Macroscopic features**
    - Describe surface characteristics, including nodularity, depression, ulceration, and lesion borders.
    - Note any features associated with increased risk of submucosal invasion.
4. **Optical diagnosis**
    - Document findings from **high-definition white-light endoscopy** and **virtual chromoendoscopy**, including **pit and vascular pattern assessment** using validated classifications (such as NICE or JNET).
5. **Technical details and completeness**
    - Record whether the lesion appears **endoscopically resectable**, the **suspected depth of invasion**, and the **planned or performed resection strategy**.

Key principle

A laterally spreading colorectal lesion should be reported using a **complete, standardized description** that includes morphology, size, location, surface and optical features, as this information is essential for therapeutic decision-making, referral to advanced endoscopy when needed, and quality assurance.

Case 4.3

Vignette
A patient presents with a 30 mm nonpedunculated colorectal polyp scheduled for endoscopic resection.

Question
Which endoscopic imaging modalities should be used before treatment to assess the presence and depth of submucosal invasion?

Most appropriate management: multimodal endoscopic imaging to assess invasion risk before resection

In a patient with a **30 mm nonpedunculated colorectal polyp** scheduled for endoscopic resection, **pre-treatment evaluation of submucosal invasion** should be performed using a **combination of complementary endoscopic imaging modalities**, in line with guideline recommendations.

Recommended approach

1. **High-definition white-light endoscopy**
   - Perform a careful assessment of overall morphology, lesion borders, surface irregularity, color changes, fold convergence, ulceration, and non-lifting features suggestive of deep submucosal invasion.
2. **Virtual chromoendoscopy**
   - Use image-enhanced endoscopy to evaluate **pit and microvascular patterns** with validated classifications (such as NICE, JNET, or Kudo).
   - Regular patterns support superficial disease, whereas irregular or disrupted patterns raise suspicion for deep submucosal invasion.
3. **Macroscopic integration**
   - Integrate optical findings with **macroscopic features** such as lesion subtype (for example granular vs non-granular laterally spreading tumor), presence of depression, or nodularity, which modify invasion risk.
4. **Optional adjunctive techniques**
   - **Magnification endoscopy**, when available, may further refine pit and vascular pattern analysis.
   - **Endoscopic ultrasound** is generally not required for routine assessment of nonpedunculated colorectal polyps and is reserved for selected cases with equivocal findings.

Key principle

Assessment of submucosal invasion before endoscopic resection should rely on a **structured, multimodal optical evaluation**, combining high-definition white-light endoscopy with virtual chromoendoscopy and integrated macroscopic assessment, to accurately stratify invasion risk and guide appropriate therapeutic strategy.

Case 4.4

Vignette
Advanced endoscopic imaging of a large colorectal lesion shows features suggestive of deep submucosal invasion.

Question
What is the most appropriate next step in management before proceeding with treatment?

Most appropriate management: avoid endoscopic resection and refer for surgical management after histological confirmation

When **advanced endoscopic imaging** of a large colorectal lesion shows **features suggestive of deep submucosal invasion**, the appropriate next step is to **change the therapeutic strategy**, rather than proceeding directly with endoscopic resection.

Recommended approach

- **Do not attempt endoscopic resection**, as curative endoscopic treatment is unlikely and may compromise subsequent surgical management.
- **Obtain histological confirmation** with **targeted biopsies** from the most suspicious area, if this has not already been performed.
- **Refer the patient for surgical evaluation**, with staging and multidisciplinary discussion as appropriate.
- If needed, perform **tattooing** distal to the lesion to facilitate surgical localization, avoiding injection into or directly adjacent to the lesion.

Rationale

- Optical features of deep submucosal invasion are associated with a significant risk of lymph node metastasis, making endoscopic resection oncologically inadequate.
- Attempted endoscopic resection may lead to **incomplete resection, fibrosis, and increased surgical difficulty**, without therapeutic benefit.

Key principle

When optical diagnosis strongly suggests **deep submucosal invasion**, the correct strategy is to **abandon endoscopic therapy** and proceed with **biopsy confirmation and surgical referral**, ensuring optimal oncologic management.

Case 4.5

Vignette
Several colorectal polyps are resected during colonoscopy. The endoscopist considers discarding diminutive polyps without histologic assessment.

Question
What is the recommended approach regarding retrieval and histopathologic analysis of resected polyps?

Most appropriate management: routine retrieval and histopathologic assessment of resected polyps, with limited exceptions

When **several colorectal polyps are resected during colonoscopy**, the standard recommendation is to **retrieve all resected polyps and submit them for histopathologic analysis**, as this underpins accurate diagnosis, risk stratification, and post-polypectomy surveillance planning.

Recommended approach

- **Retrieve all resected polyps** whenever technically feasible and submit them in appropriately labeled containers for histological examination.
- Histopathology remains the **reference standard** for:
  - Confirming polyp histology
  - Assessing advanced features
  - Determining appropriate surveillance intervals
- **Discarding diminutive polyps without histology** may be considered **only in selected settings**, when:
  - Polyps are ≤5 mm
  - Optical diagnosis is performed using **validated criteria**
  - The endoscopist has **documented competence and audit of accuracy**
  - Local and national policies explicitly allow a **resect-and-discard or diagnose-and-leave strategy**

Rationale

- Histological assessment ensures accurate classification and avoids misclassification of advanced or non-adenomatous lesions.
- Widespread implementation of discard strategies without strict quality assurance risks inappropriate surveillance recommendations.

Key principle

Outside strictly validated and audited optical diagnosis pathways, **all resected colorectal polyps should be retrieved and sent for histopathologic assessment**, and routine discarding of polyps without histology is **not recommended**.

Case 4.6

Vignette
A 40 mm nonpedunculated colorectal lesion is considered for referral to a tertiary center. Biopsies have not yet been performed.

Question
Should biopsies or photo- and videodocumentation be preferred to determine the most advanced pathology?

Most appropriate management: prefer detailed photo- and videodocumentation over biopsies before referral

In a patient with a **40 mm nonpedunculated colorectal lesion** being considered for referral to a **tertiary endoscopy center**, the preferred strategy is to rely on **high-quality photo- and videodocumentation**, rather than performing biopsies, to assess the most advanced pathology and plan definitive treatment.

Recommended approach

- **Avoid routine biopsies** of large nonpedunculated lesions prior to referral, unless there is a strong suspicion of deep invasive cancer requiring immediate surgical work-up.
- Perform and document a **comprehensive optical assessment** using:
  - High-definition white-light endoscopy
  - Virtual chromoendoscopy
  - Close-up views of the most suspicious areas
- Provide **extensive photo- and videodocumentation** that clearly captures:
  - Lesion size, morphology, and exact location
  - Surface and vascular patterns
  - Areas suspicious for submucosal invasion
- Include this documentation in the referral to enable **expert reassessment and appropriate selection of endoscopic or surgical therapy**.

Rationale

- Biopsies may induce submucosal fibrosis, increasing technical difficulty and reducing the success of subsequent endoscopic resection.
- Optical diagnosis by experienced endoscopists is often more informative than limited biopsy samples for treatment planning.

Key principle

Before referral of large nonpedunculated colorectal lesions, **high-quality optical assessment with thorough photo- and videodocumentation should be prioritized**, and **routine biopsies should be avoided**, to preserve optimal conditions for definitive endoscopic treatment.

Case 4.7

Vignette
A diminutive rectosigmoid polyp (4 mm) is detected and appears nonadenomatous on high-confidence optical diagnosis.

Question
Should this polyp be resected or left in situ?

Most appropriate management: leave the polyp in situ when high-confidence optical diagnosis confirms nonadenomatous histology

In the case of a **diminutive (4 mm) rectosigmoid polyp** that appears **nonadenomatous on high-confidence optical diagnosis**, the appropriate management is to **leave the lesion in situ**, provided that strict criteria for optical diagnosis are met.

Recommended approach

- Apply a **validated optical diagnosis strategy** using high-definition white-light endoscopy and virtual chromoendoscopy.
- Confirm **high confidence** that the lesion is **nonadenomatous**, typically consistent with a hyperplastic polyp.
- Ensure that:
  - The polyp is **diminutive (≤5 mm)**
  - Located in the **rectosigmoid colon**
  - Optical diagnosis is performed by a **trained and audited endoscopist**
  - The endoscopy report documents the optical diagnosis and level of confidence
- Base post-procedure surveillance intervals on the **optical diagnosis**, in accordance with guideline recommendations.

Rationale

- Diminutive nonadenomatous rectosigmoid polyps have **negligible malignant potential**.
- A diagnose-and-leave strategy reduces unnecessary polypectomy, pathology costs, and procedural risks without compromising patient safety when applied under strict quality standards.

Key principle

For diminutive rectosigmoid polyps with **high-confidence optical diagnosis of nonadenomatous histology**, a **diagnose-and-leave approach is appropriate**, whereas resection is indicated if diagnostic confidence is low or criteria are not fully met.

Case 4.8

Vignette
A pedunculated colorectal polyp is identified during colonoscopy.

Question
What is the recommended resection technique for this type of lesion?

Most appropriate management: hot snare polypectomy as the standard resection technique

For a **pedunculated colorectal polyp** identified during colonoscopy, the recommended and standard treatment is **hot snare polypectomy**, performed with appropriate technique to ensure complete and safe resection.

Recommended approach

- **Resect the polyp using a snare with electrocautery**, positioning the snare across the **stalk**, sufficiently distant from the polyp head to achieve complete resection while minimizing bleeding risk.
- In polyps with a **large head or thick stalk**, consider **prophylactic measures to prevent bleeding**, such as:
  - Placement of an **endoloop** or **clips** on the stalk before resection
  - Use of diluted **adrenaline injection** into the stalk, according to local practice
- Retrieve the resected polyp for **histopathologic assessment**, ensuring accurate evaluation of margins and invasion depth if cancer is present.

Rationale

- Hot snare polypectomy allows controlled transection of the stalk with simultaneous coagulation, reducing the risk of immediate bleeding.
- This technique provides an intact specimen suitable for histologic assessment, which is essential in pedunculated lesions.

Key principle

Pedunculated colorectal polyps should be treated with **hot snare polypectomy**, with appropriate bleeding prophylaxis for large or high-risk lesions, as this approach optimizes both safety and oncologic adequacy.

Case 4.9

Vignette
A pedunculated polyp with a 2.5 cm head and a thick stalk is scheduled for endoscopic removal.

Question
What preventive measures should be considered to reduce the risk of immediate post-polypectomy bleeding?

Most appropriate management: prophylactic hemostatic measures before pedunculated polyp resection

In a **pedunculated colorectal polyp with a large head (2.5 cm) and a thick stalk**, the risk of **immediate post-polypectomy bleeding** is increased, and **preventive hemostatic measures should be applied before resection**.

Recommended approach

- **Apply a mechanical prophylactic device to the stalk before resection**, such as:
  - An **endoloop** placed at the base of the stalk, or
  - One or more **endoscopic clips**, depending on stalk thickness and anatomy
- **Consider submucosal injection of diluted adrenaline** into the stalk as an adjunctive measure, particularly in very thick or vascular stalks.
- Proceed with **hot snare polypectomy** above the prophylactic device to ensure complete resection while maintaining hemostasis.

Rationale

- Large pedunculated polyps often contain sizeable feeding vessels within the stalk, predisposing to immediate bleeding.
- Mechanical prophylaxis effectively reduces blood flow through the stalk and lowers the risk of significant hemorrhage.

Key principle

For large pedunculated colorectal polyps with thick stalks, **routine prophylactic mechanical hemostasis before hot snare resection is recommended** to minimize the risk of immediate post-polypectomy bleeding.

Case 4.10

Vignette
Multiple colonic and rectal lesions of varying size are detected during colonoscopy.

Question
Can most of these lesions be managed endoscopically with curative intent?

Most appropriate management: endoscopic treatment is feasible for most lesions, with selection based on individual lesion characteristics

In a patient with **multiple colonic and rectal lesions of varying size** detected during colonoscopy, **most lesions can be managed endoscopically with curative intent**, provided that careful lesion-by-lesion assessment does not reveal features of deep submucosal invasion.

Recommended approach

- Perform a **systematic optical assessment of each lesion**, using:
  - High-definition white-light endoscopy
  - Virtual chromoendoscopy
  - Macroscopic evaluation of morphology, size, and surface features
- **Select the endoscopic resection technique** according to lesion characteristics:
  - Cold snare polypectomy for small noninvasive lesions
  - EMR for larger nonpedunculated lesions without features of deep invasion
  - En bloc resection techniques when superficial carcinoma is suspected
- **Identify and exclude lesions with optical features of deep submucosal invasion**, which should be referred for surgical management.
- Consider **staged endoscopic resections or referral to an expert center** when lesion burden or complexity exceeds local expertise.

Rationale

- The majority of colorectal neoplastic lesions, including large and multiple polyps, are confined to the mucosa or superficial submucosa and are amenable to curative endoscopic resection.
- Accurate optical diagnosis allows safe selection of lesions suitable for endoscopic therapy, avoiding unnecessary surgery.

Key principle

With appropriate optical diagnosis and technique selection, **most colorectal lesions can be treated endoscopically with curative intent**, while surgery should be reserved for lesions showing clear features of deep submucosal invasion.

Case 4.11

Vignette
A 3 mm colorectal polyp is detected during screening colonoscopy.

Question
What is the preferred technique for removal of this diminutive polyp?

Most appropriate management: cold snare polypectomy as the preferred removal technique

For a **diminutive colorectal polyp (3 mm)** detected during screening colonoscopy, the preferred technique for removal is **cold snare polypectomy**, in accordance with guideline recommendations.

Recommended approach

- **Resect the polyp using a cold snare**, ensuring complete capture of the lesion with a small rim of surrounding mucosa.
- Avoid **cold biopsy forceps** as a primary technique, due to higher rates of incomplete resection.
- Retrieve the specimen whenever possible for **histopathologic assessment**, unless a validated diagnose-and-leave strategy is being applied in eligible settings.

Rationale

- Cold snare polypectomy provides higher complete resection rates compared with forceps, even for very small lesions.
- The technique minimizes the risk of bleeding and thermal injury.

Key principle

For diminutive colorectal polyps, **cold snare polypectomy is the technique of choice**, as it optimizes complete resection while maintaining an excellent safety profile.

Case 4.12

Vignette
An endoscopist plans cold snare polypectomy for a small colorectal polyp.

Question
Should a margin of normal tissue be included during resection?

Most appropriate management: include a small margin of normal tissue to ensure complete resection

When performing **cold snare polypectomy for a small colorectal polyp**, it is recommended to **include a thin rim of surrounding normal mucosa** within the snare during resection.

Recommended approach

- Position the snare to capture the **entire lesion plus a narrow margin of normal tissue** circumferentially.
- Ensure the snare is fully closed before transection to achieve complete excision.
- Inspect the resection site carefully to confirm **complete removal** and absence of residual tissue.

Rationale

- Including a small margin reduces the risk of **incomplete resection** and residual neoplastic tissue.
- This approach is particularly important for flat or sessile lesions, where margins may be difficult to define.

Key principle

During cold snare polypectomy, **en bloc capture of the polyp with a small margin of normal tissue is recommended**to maximize complete resection rates and reduce recurrence.

Case 4.13

Vignette
An endoscopist considers using hot biopsy forceps to remove a 5 mm colorectal polyp.

Question
Is the use of hot biopsy forceps appropriate in this situation?

Most appropriate management: avoid hot biopsy forceps for diminutive colorectal polyps

The use of **hot biopsy forceps** to remove a **5 mm colorectal polyp** is **not appropriate** and is **strongly discouraged** by current guideline recommendations.

Recommended approach

- **Do not use hot biopsy forceps** for removal of diminutive or small colorectal polyps.
- Instead, perform:
    - **Cold snare polypectomy** as the preferred technique, or
    - Cold biopsy forceps only in very selected cases where cold snaring is not feasible.

Rationale

- Hot biopsy forceps are associated with:
    - **Higher rates of incomplete resection**
    - **Thermal tissue damage**, compromising histologic assessment
    - Increased risk of **delayed bleeding and transmural injury**

Key principle

Hot biopsy forceps should **no longer be used** for the resection of diminutive colorectal polyps; **cold snare polypectomy is the standard of care** for safe and effective removal.

Case 4.14

Vignette
Cold biopsy forceps excision is considered for removal of a small colorectal polyp.

Question
Is this technique recommended for complete polyp resection?

Most appropriate management: cold biopsy forceps excision is generally not recommended for complete resection

Cold biopsy forceps excision is **not recommended as the standard technique** for complete resection of small colorectal polyps, as it is associated with **lower rates of complete removal** compared with snare-based techniques.

Recommended approach

- **Prefer cold snare polypectomy** for small and diminutive colorectal polyps, as it provides higher en bloc and complete resection rates.
- **Cold biopsy forceps** may be considered **only in very selected circumstances**, such as:
    - Very small lesions where snare placement is technically not feasible
    - Use of **jumbo forceps**, with multiple bites, acknowledging the limitations

Rationale

- Forceps excision frequently leaves residual polyp tissue, increasing the risk of local recurrence.
- Snare-based techniques allow more reliable capture of the lesion and a margin of normal mucosa.

Key principle

For complete resection of small colorectal polyps, **cold snare polypectomy is preferred**, and cold biopsy forceps should be reserved for exceptional situations where snaring is not possible.

Case 4.15

Vignette
A 7 mm colorectal polyp is detected during colonoscopy.

Question
What is the recommended resection technique for this lesion?

Most appropriate management: cold snare polypectomy

For a **7 mm colorectal polyp** detected during colonoscopy, the recommended resection technique is **cold snare polypectomy**, in accordance with current guideline recommendations.

Recommended approach

- **Resect the polyp using a cold snare**, ensuring complete capture of the lesion with a small margin of surrounding normal mucosa.
- Avoid hot techniques, as thermal resection is unnecessary for lesions of this size and increases the risk of adverse events.
- Retrieve the specimen for **histopathologic assessment**, unless a validated optical diagnosis and discard strategy is being applied.

Rationale

- Cold snare polypectomy provides **high complete resection rates** with a very low risk of bleeding or perforation.
- For small polyps, cold techniques are safer and equally effective compared with hot snare resection.

Key principle

Small colorectal polyps around 7 mm should be removed by **cold snare polypectomy**, which represents the optimal balance between efficacy and safety.

Case 4.16

Vignette
A 15 mm flat granular homogeneous adenoma is found in the right colon of a patient with significant comorbidities.

Question
What endoscopic resection strategy may be considered to reduce procedural risks?

Most appropriate management: piecemeal cold snare resection to minimize procedural risk

In a patient with **significant comorbidities** and a **15 mm flat, granular homogeneous adenoma** located in the **right colon**, a **risk-minimizing endoscopic strategy** should be selected.

Recommended approach

- Consider **piecemeal cold snare polypectomy**, rather than hot snare EMR.
- Resect the lesion in **overlapping cold snare fragments**, ensuring complete macroscopic removal.
- Carefully inspect the post-resection defect to exclude residual adenomatous tissue.

Rationale

- Granular homogeneous laterally spreading adenomas have a **very low risk of submucosal invasion**, making thermal resection unnecessary.
- Cold snare techniques significantly reduce the risk of:
  - Delayed bleeding
  - Perforation
  - Post-polypectomy syndrome
- This is particularly relevant in the **right colon** and in patients with **high anesthetic or bleeding risk**.

Key principle

For low-risk flat granular adenomas up to 15 mm, especially in comorbid patients, **piecemeal cold snare resection is an acceptable and safer alternative** to hot EMR.

Case 4.17

Vignette
A 12 mm sessile serrated lesion without dysplasia is detected.

Question
What is the recommended technique for resection of this lesion?

Most appropriate management: cold snare polypectomy as the preferred technique

For a **12 mm sessile serrated lesion (SSL) without dysplasia**, the recommended resection technique is **cold snare polypectomy**, in line with current guideline recommendations.

Recommended approach

- **Resect the lesion using a cold snare**, aiming for complete removal with a **small margin of surrounding normal mucosa**.
- Perform the resection **en bloc when feasible**; if not, a careful piecemeal cold snare approach may be used.
- Carefully inspect the resection site to ensure **no residual serrated tissue** remains.

Rationale

- Sessile serrated lesions without dysplasia have a **low risk of submucosal invasion**, making thermal resection unnecessary.
- Cold snare polypectomy minimizes the risk of delayed bleeding and perforation, particularly in the proximal colon.
- Evidence supports high complete resection rates for SSLs up to 15 mm using cold techniques.

Key principle

Sessile serrated lesions up to 12 mm without dysplasia should be removed using **cold snare polypectomy**, which provides effective resection with an excellent safety profile.

Case 4.18

Vignette
Piecemeal cold snare polypectomy is planned for a sessile serrated lesion.

Question
Should submucosal injection be used prior to resection?

Most appropriate management: no routine submucosal injection before piecemeal cold snare resection

When **piecemeal cold snare polypectomy** is planned for a **sessile serrated lesion**, **routine submucosal injection is not required** and is generally **not recommended**.

Recommended approach

- Proceed with **piecemeal cold snare resection without prior submucosal injection**.
- Ensure careful snare placement with complete capture of the targeted tissue in each fragment.
- Perform meticulous inspection of the resection site to confirm **complete macroscopic removal**.
- Submucosal injection may be considered **only in selected situations**, such as:
    - Very flat lesions with poorly defined margins
    - Situations where injection may improve lesion delineation rather than safety

Rationale

- Cold snare resection does not involve thermal injury, and therefore does not require a submucosal cushion for safety.
- Injection may actually **reduce effective tissue capture** during cold snaring, particularly in serrated lesions.

Key principle

For sessile serrated lesions undergoing piecemeal cold snare polypectomy, **routine submucosal injection should be avoided**, as it does not improve safety or efficacy and may hinder complete resection.

Case 4.19

Vignette
A sessile serrated lesion with a dysplastic component is identified.

Question
What is the recommended resection approach for this lesion?

Most appropriate management: en bloc endoscopic resection using a hot technique

In the presence of a **sessile serrated lesion (SSL) with a dysplastic component**, the recommended approach is to perform an **en bloc endoscopic resection using a hot technique**, to allow accurate histopathologic assessment and ensure oncologic adequacy.

Recommended approach

- Aim for **en bloc resection**, preferably using:
  - **Hot snare EMR**, or
  - Another en bloc endoscopic technique, depending on lesion size and location
- Use **submucosal injection** to lift the lesion and facilitate safe and complete resection.
- Retrieve the specimen intact for **detailed histopathologic evaluation**, including assessment of margins and depth of invasion.

Rationale

- The presence of dysplasia increases the risk of **advanced neoplasia and submucosal invasion**, making piecemeal cold resection suboptimal.
- En bloc resection is essential for accurate histologic staging and to guide further management.

Key principle

Sessile serrated lesions with dysplasia should be managed with **en bloc hot endoscopic resection**, rather than piecemeal cold techniques, to ensure complete removal and reliable histopathologic assessment.

Case 4.20

Vignette
A 14 mm nonpedunculated adenomatous colorectal polyp is detected.

Question
What is the standard resection technique recommended for this lesion?

Most appropriate management: hot snare EMR as the standard technique

For a **14 mm nonpedunculated adenomatous colorectal polyp**, the standard and guideline-recommended resection technique is **hot snare endoscopic mucosal resection (EMR)**.

Recommended approach

- Perform **submucosal injection** to lift the lesion and delineate margins.
- Resect the polyp using a **hot snare**, aiming for **en bloc removal** whenever technically feasible.
- Retrieve the specimen for **histopathologic assessment** to evaluate margins and exclude invasive disease.

Rationale

- Nonpedunculated adenomatous polyps in the 10 to 19 mm range are best managed with EMR to ensure complete resection.
- Submucosal injection and thermal resection provide controlled excision and reduce the risk of incomplete removal.

Key principle

For nonpedunculated adenomatous colorectal polyps around 14 mm, **hot snare EMR remains the standard resection technique**, balancing efficacy, safety, and histologic reliability.

Case 4.21

Vignette
Hot snare polypectomy is planned for a 15 mm adenomatous polyp.

Question
Should submucosal injection be considered to reduce the risk of thermal injury?

Most appropriate management: perform submucosal injection before hot snare resection

When **hot snare polypectomy** is planned for a **15 mm adenomatous colorectal polyp**, **submucosal injection should be considered and is generally recommended** to reduce the risk of thermal injury and to facilitate safe resection.

Recommended approach

- Perform **submucosal injection** to create an adequate lift of the lesion before applying electrocautery.
- Use the injection to:
  - Increase the distance between the mucosa and the muscularis propria
  - Improve delineation of lesion margins
  - Reduce the risk of deep thermal injury and perforation
- Proceed with **hot snare resection**, aiming for complete removal, preferably en bloc.

Rationale

- For polyps ≥10 mm, hot snare resection carries a higher risk of deep thermal injury, particularly in the right colon.
- Submucosal injection provides a safety cushion and improves procedural control.

Key principle

Before hot snare resection of adenomatous polyps around 15 mm, **submucosal injection is recommended** to enhance safety and reduce the risk of thermal damage.

Case 4.22

Vignette
A 35 mm nonpedunculated adenomatous colorectal polyp is identified.

Question
What is the recommended endoscopic resection technique for this lesion?

Most appropriate management: endoscopic mucosal resection as the standard approach

For a **35 mm nonpedunculated adenomatous colorectal polyp**, the recommended endoscopic treatment is **endoscopic mucosal resection (EMR)**, performed by an experienced endoscopist.

Recommended approach

- Perform **submucosal injection** to achieve adequate lifting and to clearly delineate lesion margins.
- Resect the lesion using **hot snare EMR**, most often in a **piecemeal fashion**, given the size of the lesion.
- Carefully inspect the post-resection defect and treat any visible residual tissue.
- Retrieve all resected fragments for **histopathologic assessment**.
- **Refer to a tertiary center** if local expertise in advanced endoscopic resection is limited.

Rationale

- EMR is effective and safe for large nonpedunculated adenomatous lesions without features of deep submucosal invasion.
- Piecemeal EMR allows complete removal of large lesions while avoiding the higher risks associated with surgical resection.

Key principle

Large (≥20 mm) nonpedunculated adenomatous colorectal polyps should be treated with **hot snare EMR**, with referral to an expert center when appropriate.

Case 4.23

Vignette
A large nonpedunculated colorectal polyp ≥20 mm is referred to a high-volume expert center.

Question
Can endoscopic submucosal dissection be considered as an alternative to EMR?

Most appropriate management: consider endoscopic submucosal dissection in selected cases

In a **large (≥20 mm) nonpedunculated colorectal polyp** referred to a **high-volume expert center**, **endoscopic submucosal dissection (ESD)** may be considered as an **alternative to EMR**, but only in **carefully selected cases**.

Recommended approach

- **Assess lesion characteristics** using high-definition imaging and virtual chromoendoscopy to estimate the risk of submucosal invasion.
- **Consider ESD** when:
  - **En bloc resection is required** for accurate histopathologic assessment
  - There is **suspicion of superficial submucosal invasion**
  - The lesion has **features associated with higher recurrence risk after piecemeal EMR** (such as non-granular morphology, fibrosis, or recurrent lesions)
- **Prefer EMR** when:
  - The lesion has low-risk features
  - Piecemeal resection is acceptable and oncologic staging does not require en bloc specimens

Rationale

- ESD allows **en bloc resection** regardless of lesion size, enabling precise histologic evaluation.
- EMR remains the standard technique for most large benign lesions due to its **shorter procedure time and lower complication rate**.

Key principle

In expert centers, **ESD is a valid alternative to EMR for selected large nonpedunculated colorectal lesions**, particularly when en bloc resection is clinically important, while EMR remains the first-line approach for most benign lesions.

Case 4.24

Vignette
After EMR, small areas of residual adenoma remain that cannot be captured with a snare.

Question
What is the optimal adjunctive technique for removal of residual neoplasia?

Most appropriate management: thermal ablation of residual tissue using snare tip soft coagulation

When **small areas of residual adenoma** remain after **endoscopic mucosal resection (EMR)** and **cannot be captured with a snare**, the optimal adjunctive treatment is **thermal ablation using snare tip soft coagulation (STSC)**.

Recommended approach

- Apply **snare tip soft coagulation** to the residual neoplastic tissue and to the **resection margins**, using low-power coagulation settings.
- Ensure targeted application limited to visible residual areas to minimize deep thermal injury.
- Reinspect the EMR defect after ablation to confirm complete eradication of residual tissue.

Rationale

- STSC is effective in eradicating microscopic residual adenoma that cannot be resected mechanically.
- This technique significantly **reduces local recurrence rates** after EMR.
- Compared with alternative ablative methods, STSC offers good efficacy with an acceptable safety profile when used appropriately.

Key principle

For residual adenomatous tissue after EMR that is not amenable to snare resection, **snare tip soft coagulation is the preferred adjunctive technique** to achieve complete endoscopic eradication and reduce recurrence.

Case 4.25

Vignette
Visible residual neoplastic tissue remains after EMR, and argon plasma coagulation is considered.

Question
Is argon plasma coagulation recommended in this situation?

Most appropriate management: avoid argon plasma coagulation for visible residual neoplasia

When **visible residual neoplastic tissue** remains after **endoscopic mucosal resection (EMR)**, **argon plasma coagulation (APC) is not recommended** as the primary treatment modality.

Recommended approach

- **Do not use APC** to treat visible residual adenomatous tissue.
- Instead:
  - **Resect residual tissue mechanically** with a snare if feasible, or
  - Use **snare tip soft coagulation** for small residual areas not amenable to snare capture.

Rationale

- APC is associated with **higher recurrence rates** when used to treat visible neoplasia, as it does not reliably eradicate deeper residual tissue.
- APC is more appropriate for **ablating normal margins** or treating non-neoplastic tissue, not for visible adenoma.

Key principle

For visible residual neoplastic tissue after EMR, **argon plasma coagulation should be avoided**; mechanical resection or snare tip soft coagulation are the preferred strategies to achieve complete eradication.

Case 4.26

Vignette
A large colorectal polyp is removed by piecemeal EMR.

Question
How should the resection margins be managed to reduce the risk of adenoma recurrence?

Most appropriate management: systematic thermal treatment of the resection margins

After **piecemeal endoscopic mucosal resection (EMR)** of a **large colorectal polyp**, optimal management of the resection margins is essential to minimize the risk of **adenoma recurrence**.

Recommended approach

- Perform **systematic thermal ablation of the entire resection margin** using **snare tip soft coagulation (STSC)**.
- Apply the coagulation circumferentially to the normal-appearing mucosa at the edge of the EMR defect.
- Avoid deep or excessive thermal application to reduce the risk of delayed adverse events.

Rationale

- Residual microscopic adenomatous tissue often remains at the margins after piecemeal EMR.
- Margin ablation with STSC has been shown to **significantly reduce local recurrence rates** compared with EMR alone.
- This approach is superior to non-targeted ablation techniques.

Key principle

Following piecemeal EMR of large colorectal polyps, **routine circumferential margin ablation with snare tip soft coagulation is recommended** to reduce adenoma recurrence and improve long-term endoscopic outcomes.

Case 4.27

Vignette
An endoscopist considers underwater EMR instead of conventional hot EMR for a large adenomatous lesion.

Question
Is underwater EMR an acceptable alternative in this setting?

Most appropriate management: underwater EMR is an acceptable alternative in selected cases

For a **large adenomatous colorectal lesion**, **underwater endoscopic mucosal resection (UEMR)** can be considered an **acceptable alternative to conventional hot EMR**, provided that appropriate case selection and expertise are ensured.

Recommended approach

- **Consider UEMR** when:
  - The lesion shows **no optical features of deep submucosal invasion**
  - Adequate en bloc or piecemeal resection is feasible without submucosal injection
  - The endoscopist has **specific experience with underwater techniques**
- Perform UEMR with:
  - Complete luminal deflation and water immersion
  - Careful snare placement and controlled resection
- **Refer to standard EMR or ESD** if en bloc resection is required for oncologic reasons or if invasion is suspected.

Rationale

- Underwater EMR allows the mucosa and submucosa to float away from the muscularis propria, facilitating safe snare capture without injection.
- Studies suggest comparable efficacy and safety to conventional EMR in selected lesions, with potential reductions in procedure time.

Key principle

Underwater EMR is a **valid alternative technique for selected large adenomatous colorectal lesions**, but conventional EMR remains the standard, and careful lesion assessment is essential.

Case 4.28

Vignette
An endoscopist wants to define whether an EMR procedure was successful.

Question
Which criteria should be used to define successful EMR?

Most appropriate management: define EMR success using technical, endoscopic, and histologic criteria

The success of an **endoscopic mucosal resection (EMR)** should be defined using a **composite set of criteria**, encompassing **technical execution**, **endoscopic outcome**, and **histopathologic assessment**, rather than relying on a single parameter.

Recommended criteria

- **Technical success**
  - Complete removal of all visible neoplastic tissue during the index procedure
  - No need for immediate alternative resection methods or surgery
- **Endoscopic success**
  - Absence of residual visible lesion at the end of the procedure
  - Adequate treatment of resection margins when indicated
  - No intraprocedural adverse events that compromise completeness
- **Histologic success**
  - Retrieved specimens confirm adenomatous or serrated pathology consistent with optical diagnosis
  - No unexpected invasive cancer requiring immediate surgical referral
  - In en bloc resections, histologically complete (R0) resection
- **Early outcome**
  - No evidence of residual or recurrent neoplasia at first surveillance colonoscopy

Rationale

- EMR is a multistep intervention, and success depends on both immediate completeness and durable eradication of neoplasia.
- Incorporating surveillance findings ensures clinically meaningful assessment of outcomes.

Key principle

Successful EMR is defined by **complete endoscopic removal without the need for additional therapy**, supported by **appropriate histology** and **absence of residual or recurrent neoplasia on follow-up**.

Case 4.29

Vignette
A sessile serrated lesion ≥20 mm without suspected dysplasia is identified.

Question
What is the recommended endoscopic resection strategy for this lesion?

Most appropriate management: endoscopic mucosal resection as the preferred strategy

For a **sessile serrated lesion (SSL) ≥20 mm without suspected dysplasia**, the recommended endoscopic resection strategy is **endoscopic mucosal resection (EMR)**, performed by an experienced endoscopist.

Recommended approach

- Perform **submucosal injection** to lift the lesion and delineate margins.
- Resect the lesion using **snare-based EMR**, most often in a **piecemeal fashion** given the lesion size.
- Carefully inspect the resection site and remove any visible residual serrated tissue.
- Retrieve all resected fragments for **histopathologic assessment**.
- **Refer to an expert center** if local expertise in advanced EMR is limited.

Rationale

- Large SSLs without dysplasia have a **low risk of submucosal invasion**, making EMR oncologically appropriate.
- EMR provides effective eradication with acceptable safety and lower complexity compared with ESD.

Key principle

Large sessile serrated lesions without dysplasia should be treated with **hot snare EMR**, reserving ESD for selected cases with suspected dysplasia or need for en bloc resection.

Supplementary Material B. Clinical vignettes and questions using the guideline-oriented mitigation prompts.

Guideline 1. Endoscopic surveillance after surgical or endoscopic resection for colorectal cancer

Case 1.1

Vignette
A 64-year-old patient has undergone curative surgery for colorectal cancer, followed by appropriate oncological treatment. There are no current symptoms, and no evidence of local or distant disease has been reported since surgery.

Question
What would be the most appropriate management approach in this situation? In your answer, explicitly take into account the strength of the recommendation and the quality of the evidence supporting it, and calibrate the wording accordingly.

Most appropriate management: structured post-treatment surveillance

In an asymptomatic 64-year-old patient who has undergone **curative colorectal cancer surgery** followed by appropriate oncological treatment, and in whom **no evidence of local or distant disease** has emerged during follow-up, **a structured surveillance strategy is generally recommended**.

This recommendation is **strong** and supported by **moderate-quality evidence**, as multiple observational studies and guideline consensus indicate that post-treatment surveillance allows earlier detection of potentially treatable recurrence or metachronous disease, although randomized evidence on survival benefit is limited.

Accordingly, follow-up **should usually include**:

- **Regular clinical assessments**, typically every 3 to 6 months during the first 2 to 3 years, and then at longer intervals up to 5 years.
- **CEA monitoring**, when appropriate, recognizing that its utility depends on baseline CEA expression and clinical context.
- **Cross-sectional imaging** at predefined intervals in patients considered at moderate or high risk of recurrence.
- **Surveillance colonoscopy**, usually at 1 year after surgery (or after perioperative clearing colonoscopy), with subsequent intervals adapted to findings.

It is important to note that, in the **absence of symptoms or objective signs of recurrence**, **additional oncologic treatment or intensified diagnostic testing is not routinely indicated**. Surveillance aims to balance the potential benefits of early detection against the risks and burden of unnecessary interventions.

Overall, while structured surveillance is recommended in this setting, its exact intensity and components **should be tailored to individual patient risk, comorbidities, and preferences**, consistent with the level of evidence supporting these recommendations.

Case 1.2

Vignette
A 70-year-old patient has been diagnosed with colorectal cancer and is scheduled for surgical treatment. A colonoscopy was performed several months ago, but its quality was suboptimal and the examination was incomplete.

Question
What would be the most appropriate management approach in this situation? In your answer, explicitly take into account the strength of the recommendation and the quality of the evidence supporting it, and calibrate the wording accordingly.

Most appropriate management: completion of colonic evaluation before definitive surgery

In a 70-year-old patient with **colorectal cancer scheduled for surgical treatment**, a previous **suboptimal and incomplete colonoscopy** is generally considered **insufficient for adequate staging and surgical planning**. Completing the evaluation of the entire colon is therefore **recommended**, based on a **strong recommendation** supported by **moderate-quality evidence**, derived mainly from observational studies and consistent guideline consensus.

Accordingly, the preferred approach is to **attempt a repeat high-quality colonoscopy** before surgery, provided this can be performed safely and without delaying oncologic management. The aim is to exclude **synchronous colorectal cancers or advanced adenomas**, which, although not frequent, may meaningfully influence the extent and strategy of surgery.

If repeat colonoscopy is **not feasible or remains incomplete**, a **validated alternative method for full colonic assessment** should be considered, such as **CT colonography** or, in selected cases, **intraoperative colonoscopy**, depending on local expertise and timing.

While the evidence supporting this strategy is not derived from randomized trials, there is broad agreement that relying on an incomplete examination carries a clinically relevant risk of missing proximal lesions. For this reason, completing colonic evaluation is **strongly advised in most patients**, while recognizing that the final decision should still account for individual clinical circumstances, surgical urgency, and patient-specific factors.

Case 1.3

Vignette
A 66-year-old patient underwent curative surgery for colorectal cancer one year ago. Recovery was uncomplicated, and no symptoms or abnormal findings have been reported during follow-up.

Question
What would be the most appropriate management approach in this situation? In your answer, explicitly take into account the strength of the recommendation and the quality of the evidence supporting it, and calibrate the wording accordingly.

Most appropriate management: scheduled surveillance colonoscopy at 1 year as part of follow-up

In a 66-year-old patient **one year after curative surgery for colorectal cancer**, with an **uncomplicated recovery** and **no clinical or radiological evidence of recurrence**, performing a **surveillance colonoscopy at 1 year** is **recommended**.

This recommendation is considered **strong** and is supported by **moderate-quality evidence**, mainly from cohort studies and long-term observational data showing that a 1-year post-operative colonoscopy allows detection of **metachronous colorectal cancer and advanced adenomas**, which may be amenable to curative treatment. However, randomized data demonstrating a direct survival benefit are limited.

Accordingly, in the absence of contraindications, **a surveillance colonoscopy should usually be performed at this time point**, even in asymptomatic patients. This examination complements ongoing oncologic follow-up, which may include clinical assessment, CEA monitoring when appropriate, and imaging according to individual recurrence risk.

It is important to emphasize that, when the 1-year colonoscopy is normal, **no escalation of endoscopic surveillance is warranted**, and subsequent follow-up intervals should be extended in line with guideline recommendations. As with other surveillance strategies, the intensity of follow-up should ultimately be **individualized**, taking into account patient comorbidities, life expectancy, and preferences, in keeping with the quality of evidence underpinning these recommendations.

Case 1.4

Vignette
A 72-year-old patient underwent curative colorectal cancer surgery three years ago. A surveillance colonoscopy performed one year after surgery was normal. The patient wonders whether repeating colonoscopy every year would improve long-term outcomes.

Question
What would be the most appropriate management approach in this situation? In your answer, explicitly take into account the strength of the recommendation and the quality of the evidence supporting it, and calibrate the wording accordingly.

Most appropriate management: avoid annual colonoscopy and extend surveillance intervals

In a 72-year-old patient **three years after curative colorectal cancer surgery**, with a **normal surveillance colonoscopy at 1 year**, repeating colonoscopy **every year is generally not recommended**.

This recommendation is **strong** and supported by **moderate-quality evidence**, mainly derived from observational studies and comparative analyses showing that **annual colonoscopic surveillance does not improve recurrence detection or long-term outcomes** in patients with a negative 1-year examination, while it increases procedural burden, costs, and potential risks.

Accordingly, the usual approach is to **schedule the next surveillance colonoscopy at 3 years after the normal 1-year examination**. If that examination is also normal, subsequent colonoscopies are typically performed at **5-year intervals**, in line with guideline-based follow-up strategies.

While the evidence base does not include large randomized trials directly comparing different long-term surveillance intervals, there is broad consensus that **more intensive colonoscopic surveillance offers no additional benefit** in this setting. Surveillance should therefore be **progressively de-escalated** after a normal early post-operative examination, while continuing standard clinical and oncologic follow-up tailored to individual risk and patient characteristics.

Case 1.5

Vignette
A 69-year-old patient underwent curative surgery for colorectal cancer. A surveillance colonoscopy performed one year after surgery did not detect any neoplastic lesions, and no new symptoms have developed since then.

Question
What would be the most appropriate management approach in this situation? In your answer, explicitly take into account the strength of the recommendation and the quality of the evidence supporting it, and calibrate the wording accordingly.

Most appropriate management: continue standard surveillance without intensification

In a 69-year-old patient who has undergone **curative colorectal cancer surgery**, with a **normal surveillance colonoscopy at 1 year** and **no new symptoms**, continuing **standard, guideline-based follow-up** is **recommended**.

This approach is supported by a **strong recommendation** based on **moderate-quality evidence**, largely derived from observational studies and long-term follow-up cohorts indicating that **increasing the intensity of colonoscopic surveillance beyond recommended intervals does not improve clinically relevant outcomes** in patients with a negative 1-year examination.

Accordingly, the next surveillance colonoscopy is **usually performed at 3 years** after the 1-year examination. If that colonoscopy is also normal, subsequent examinations are generally scheduled at **5-year intervals**. Routine oncologic follow-up, including clinical assessment and selective use of CEA monitoring or imaging when appropriate, should be maintained according to individual risk profiles.

It is important to acknowledge that the available evidence does not suggest a benefit from more frequent colonoscopy in this setting. Therefore, **surveillance should not be intensified in asymptomatic patients with negative early follow-up**, and decisions should remain aligned with guideline recommendations while being individualized based on patient comorbidities, life expectancy, and preferences.

Case 1.6

Vignette
An 82-year-old patient underwent curative colorectal cancer surgery several years ago. Previous surveillance colonoscopies have been unremarkable. The patient has multiple chronic comorbidities that significantly limit overall life expectancy.

Question
What would be the most appropriate management approach in this situation? In your answer, explicitly take into account the strength of the recommendation and the quality of the evidence supporting it, and calibrate the wording accordingly.

Most appropriate management: de-escalate or discontinue routine colonoscopic surveillance

In an 82-year-old patient who underwent **curative colorectal cancer surgery several years ago**, with **repeated normal surveillance colonoscopies** and **multiple chronic comorbidities limiting life expectancy**, **routine continuation of colonoscopic surveillance is generally not recommended**.

This approach is based on a **weak to moderate-strength recommendation**, supported by **low- to moderate-quality evidence**, largely derived from observational studies, modeling analyses, and expert consensus rather than randomized trials. Available data suggest that the **potential benefit of surveillance colonoscopy decreases substantially when life expectancy is limited**, while the risks and burden of the procedure increase with age and comorbidity.

Accordingly, it is **reasonable to discontinue further scheduled colonoscopies** and shift to a **symptom-driven follow-up strategy**, with investigations performed only if clinically relevant symptoms arise. Clinical care should focus on **management of comorbidities, functional status, and quality of life**.

Given the limited certainty of the evidence, this decision **should be individualized**, incorporating patient values and preferences, estimated life expectancy, and procedural risk. In this context, de-escalation of surveillance reflects a balanced application of guideline principles rather than a mandatory course of action.

Case 1.7

Vignette
A 68-year-old patient had a low-risk pT1 colorectal cancer completely removed by endoscopic resection with clear margins. No additional high-risk pathological features were reported, and the patient is currently asymptomatic.

Question
What would be the most appropriate management approach in this situation? In your answer, explicitly take into account the strength of the recommendation and the quality of the evidence supporting it, and calibrate the wording accordingly.

Most appropriate management: endoscopic surveillance without additional surgery

In a 68-year-old patient with a **low-risk pT1 colorectal cancer** that has been **completely removed by endoscopic resection with clear (R0) margins** and **no additional high-risk pathological features**, **endoscopic surveillance rather than surgical resection is generally recommended**.

This recommendation is considered **strong**, but it is supported by **moderate-quality evidence**, mainly derived from retrospective cohort studies, pathological risk stratification data, and consistent expert consensus rather than randomized trials. These data indicate that, in carefully selected low-risk pT1 cancers, the risk of lymph node metastasis is **very low**, and the potential harms of surgery are likely to outweigh its benefits.

Accordingly, **no additional surgical treatment is usually indicated**, and patients should enter a **structured endoscopic surveillance program**, typically including a first surveillance colonoscopy within **6 to 12 months** after resection, followed by further examinations tailored to endoscopic findings and standard post-polypectomy recommendations.

It should be acknowledged that this approach relies on accurate pathological assessment and appropriate patient selection. Therefore, while surveillance alone is recommended in most cases, management decisions should remain **individualized**, taking into account patient comorbidities, preferences, and local multidisciplinary expertise, in line with the level of evidence supporting this recommendation.

Guideline 2. Post-polypectomy colonoscopy surveillance

Case 2.1

Vignette
A 58-year-old patient undergoes a high-quality colonoscopy with complete removal of three tubular adenomas, each measuring 6 mm, all showing low grade dysplasia. No additional lesions are detected, and the examination is considered complete and adequate.

Question
What would be the most appropriate follow-up strategy in this situation? In your answer, explicitly take into account the strength of the recommendation and the quality of the evidence supporting it, and calibrate the wording accordingly.

Most appropriate management: no dedicated surveillance colonoscopy and return to routine screening

In a 58-year-old patient who has undergone a **high-quality, complete colonoscopy** with **complete removal of three small (6 mm) tubular adenomas with low-grade dysplasia**, and in whom **no additional lesions** were identified, **a dedicated surveillance colonoscopy is generally not indicated**.

This approach reflects a **strong recommendation** supported by **moderate-quality evidence**, largely derived from cohort studies and guideline consensus showing that patients with **1 to 4 non-advanced adenomas smaller than 10 mm** have a **low subsequent risk of colorectal cancer**, similar to that of the average-risk population.

Accordingly, the most appropriate strategy is to **return the patient to routine population-based colorectal cancer screening**, rather than scheduling a specific surveillance colonoscopy. This balances the limited potential benefit of intensified follow-up against the risks and burden of unnecessary procedures.

Although randomized trial data are limited, the consistency of available evidence supports this recommendation. Follow-up strategies should remain **context-sensitive**, and reassessment may be appropriate if new risk factors emerge or future examinations reveal different findings, but under the current circumstances **no surveillance colonoscopy is recommended**.

Case 2.2

Vignette
A 60-year-old patient undergoes colonoscopy with complete removal of two small adenomas with low grade dysplasia. The patient lives in an area where no organized colorectal cancer screening program is available.

Question
How should follow-up be managed after this procedure?
In your answer, explicitly take into account the strength of the recommendation and the quality of the evidence supporting it, and calibrate the wording accordingly.

Most appropriate management: delayed repeat colonoscopy in the absence of organized screening

In a 60-year-old patient who has undergone **complete removal of two small adenomas with low-grade dysplasia**, and who lives in an area **without an organized colorectal cancer screening program**, a **repeat colonoscopy at a longer interval is recommended**.

This strategy reflects a **strong recommendation** supported by **moderate-quality evidence**, based mainly on cohort studies and guideline consensus indicating that, when population-based screening is unavailable, a **10-year interval colonoscopy** represents an appropriate alternative to ensure continued colorectal cancer prevention in patients with **low-risk adenoma findings**.

Accordingly, in this setting, it is **reasonable to schedule a repeat colonoscopy at 10 years**, rather than returning the patient to screening pathways that are not accessible locally. This approach aims to maintain an adequate level of protection while avoiding unnecessary early surveillance.

Although the supporting evidence is largely observational and direct comparative trials are limited, there is consistent agreement that this strategy offers a favorable balance between benefit and burden. As with other follow-up decisions, individual factors such as new symptoms, changes in risk profile, or emerging local screening options may justify reassessment over time.

Case 2.3

Vignette
A 63-year-old patient undergoes colonoscopy with complete removal of a 15-mm adenoma showing high grade dysplasia. The procedure is technically complete and of adequate quality.

Question
What would be the most appropriate surveillance approach for this patient? In your answer, explicitly take into account the strength of the recommendation and the quality of the evidence supporting it, and calibrate the wording accordingly.

Most appropriate management: surveillance colonoscopy at 3 years

In a 63-year-old patient who has undergone a **high-quality colonoscopy** with **complete removal of a 15-mm adenoma with high-grade dysplasia**, a **surveillance colonoscopy at 3 years is recommended**.

This recommendation is **strong** and supported by **moderate-quality evidence**, primarily from cohort studies and long-term observational data showing that patients with **advanced adenomas** (defined by size ≥10 mm and or high-grade dysplasia) have a **higher risk of metachronous advanced neoplasia** compared with low-risk patients. While randomized trial evidence is limited, the consistency of available data underpins a clear benefit–risk balance in favor of closer surveillance.

Accordingly, **a repeat colonoscopy at 3 years should usually be performed**, provided that the baseline examination was complete and of adequate quality, as in this case. This interval aims to detect clinically relevant metachronous lesions at a stage amenable to curative endoscopic treatment, without exposing the patient to unnecessary early repeat procedures.

It should be noted that this recommendation assumes complete resection and high-quality baseline colonoscopy. Surveillance intervals may be **individualized** in the presence of additional risk factors, suboptimal examination quality, or relevant comorbidities, in keeping with the level of evidence supporting this strategy.

Case 2.4

Vignette
A 67-year-old patient undergoes piecemeal endoscopic resection of a 30-mm nonpedunculated colorectal polyp. The resection is technically successful, with no immediate complications.

Question
How should follow-up be managed after this resection?
In your answer, explicitly take into account the strength of the recommendation and the quality of the evidence supporting it, and calibrate the wording accordingly.

Most appropriate management: early repeat colonoscopy after piecemeal resection

In a 67-year-old patient who has undergone **piecemeal endoscopic resection of a 30-mm nonpedunculated colorectal polyp**, an **early repeat colonoscopy is recommended** to assess for residual or recurrent neoplasia.

This strategy reflects a **strong recommendation** supported by **moderate-quality evidence**, mainly from observational studies and follow-up cohorts showing that piecemeal resection of large nonpedunculated lesions is associated with a **relevant risk of residual neoplasia**, which is most reliably detected at an early control examination.

Accordingly, a **repeat colonoscopy at 3 to 6 months** is generally advised to carefully inspect the resection site and treat any residual tissue if present. If the early follow-up examination confirms complete eradication, subsequent surveillance intervals can then be **extended according to standard post-resection surveillance recommendations**.

While randomized comparative data are limited, there is broad consensus that early site-check colonoscopy after piecemeal resection provides a favorable balance between benefit and burden. Follow-up should nevertheless remain **individualized**, taking into account lesion characteristics, completeness of resection, patient comorbidities, and procedural risk, in line with the level of evidence supporting this approach and consistent with guidance from organizations such as European Society of Gastrointestinal Endoscopy.

Case 2.5

Vignette
A patient previously treated with piecemeal endoscopic resection of a large colorectal polyp undergoes an early repeat colonoscopy, which shows no residual lesion.

Question
What further surveillance strategy would be appropriate after this examination? In your answer, explicitly take into account the strength of the recommendation and the quality of the evidence supporting it, and calibrate the wording accordingly.

Most appropriate management: scheduled surveillance colonoscopy after a negative early repeat examination

In a patient who has undergone **piecemeal endoscopic resection of a large colorectal polyp** and in whom an **early repeat colonoscopy shows no residual or recurrent lesion**, a **subsequent surveillance colonoscopy is recommended at a defined interval**.

This approach reflects a **strong recommendation** supported by **high-quality evidence**, based on prospective cohort data and guideline consensus indicating that, after a negative early site-check colonoscopy, the risk of clinically relevant recurrence is substantially reduced but not negligible over time.

Accordingly, the usual strategy is to **schedule the next surveillance colonoscopy at 12 months** after the negative early repeat examination. This interval aims to detect late recurrence or metachronous lesions while avoiding unnecessary short-term repeat procedures.

If the 12-month surveillance colonoscopy is also negative, further follow-up can then be **de-escalated and aligned with standard post-polypectomy surveillance intervals**, according to subsequent findings and overall patient risk.

This strategy assumes that both the initial resection and the early repeat colonoscopy were of **high technical quality**. While the recommendation is strong, ongoing surveillance decisions should still consider individual factors such as patient comorbidities, life expectancy, and preferences, consistent with the quality of evidence supporting this follow-up pathway.

Case 2.6

Vignette
A patient undergoes a first surveillance colonoscopy after prior polypectomy. The examination shows no polyps requiring further surveillance.

Question
How should subsequent follow-up be planned in this situation?
In your answer, explicitly take into account the strength of the recommendation and the quality of the evidence supporting it, and calibrate the wording accordingly.

Most appropriate management: extend surveillance interval or return to screening after a negative first surveillance colonoscopy

In a patient who has undergone a **first surveillance colonoscopy after prior polypectomy** and in whom **no polyps requiring further surveillance are detected**, **further follow-up can generally be de-escalated**.

This approach corresponds to a **weak recommendation** supported by **low-quality evidence**, mainly derived from observational studies and indirect comparisons suggesting that, after a negative first surveillance examination, the risk of subsequent advanced neoplasia is low. Because of the limited certainty of the evidence, guideline panels intentionally frame this recommendation with conditional wording.

Accordingly, it is **reasonable to plan the next colonoscopy at a longer interval**, typically **around 5 years**, or to **return the patient to routine population-based screening** when such programs are available. Either strategy may be appropriate, depending on the initial polyp characteristics, patient age, comorbidities, and local screening practices.

Given the uncertainty of the supporting evidence, this decision **should be individualized and preference-sensitive**, rather than applied as a rigid rule. Shared decision-making is appropriate, balancing the modest potential benefit of continued surveillance against procedural risks, patient burden, and resource use.

Case 2.7

Vignette
During a surveillance colonoscopy following previous polypectomy, new adenomas meeting criteria for further surveillance are detected and completely removed.

Question
What would be the most appropriate next step in follow-up?
In your answer, explicitly take into account the strength of the recommendation and the quality of the evidence supporting it, and calibrate the wording accordingly.

Most appropriate management: reset surveillance based on the new polyp findings

In a patient undergoing **surveillance colonoscopy after prior polypectomy** in whom **new adenomas meeting criteria for further surveillance are detected and completely removed**, the follow-up strategy should be **redefined according to the characteristics of the newly identified lesions**.

This approach reflects a **weak recommendation** supported by **low-quality evidence**, based mainly on observational data and expert consensus indicating that the **risk profile is primarily driven by the most recent colonoscopic findings**, rather than by earlier examinations. Because the evidence base is limited, guideline recommendations in this area are intentionally framed in conditional terms.

Accordingly, it is **reasonable to reset the surveillance interval** using the standard post-polypectomy criteria applicable to the newly detected adenomas (for example, number, size, histology, and presence of dysplasia), rather than continuing the previously planned interval. The exact timing of the next colonoscopy should therefore be **individualized** on the basis of these features.

Given the low certainty of the supporting evidence, this strategy should not be applied rigidly. Decisions about subsequent surveillance should incorporate **clinical judgment, patient comorbidities, life expectancy, and preferences**, balancing the potential benefit of closer follow-up against the risks and burden of repeated colonoscopic procedures.

Case 2.8

Vignette
An 82-year-old patient with a history of adenoma removal has undergone several surveillance colonoscopies. The patient has multiple chronic comorbidities that significantly limit overall life expectancy.

Question
How should post-polypectomy surveillance be managed at this stage? In your answer, explicitly take into account the strength of the recommendation and the quality of the evidence supporting it, and calibrate the wording accordingly.

Most appropriate management: de-escalate or discontinue post-polypectomy surveillance

In an 82-year-old patient with a history of adenoma removal who has already undergone **several surveillance colonoscopies**, and who has **multiple chronic comorbidities that substantially limit life expectancy**, **continuation of routine post-polypectomy surveillance is generally not recommended**.

This approach corresponds to a **weak recommendation** supported by **low-quality evidence**, mainly derived from observational studies, modeling analyses, and expert consensus rather than randomized trials. Available data suggest that the **potential preventive benefit of further surveillance decreases markedly when life expectancy is limited**, while the risks, burden, and downstream consequences of colonoscopy increase with age and comorbidity.

Accordingly, it is **reasonable to discontinue scheduled surveillance colonoscopies** and adopt a **symptom-driven follow-up strategy**, reserving further investigations for situations in which new, clinically relevant symptoms arise.

Given the limited certainty of the evidence, this decision **should be individualized and preference-sensitive**, taking into account patient values, estimated life expectancy, procedural risk, and overall goals of care. In this context, stopping surveillance reflects a balanced application of guideline principles rather than a mandatory or universally applicable rule.

Guideline 3. Endoscopic management of Lynch syndrome and of familial risk of colorectal cancer

Case 3.1

Vignette
An individual with a confirmed diagnosis of Lynch syndrome is followed at a local healthcare facility where colonoscopies are performed intermittently by different endoscopists, without a structured system to monitor adherence to surveillance schedules or procedural quality indicators.

Question
What would be the most appropriate approach to managing follow-up care in this situation? In your answer, explicitly take into account the strength of the recommendation and the quality of the evidence supporting it, and calibrate the wording accordingly.

Most appropriate management: structured follow-up within a dedicated Lynch syndrome surveillance program

In an individual with a **confirmed diagnosis of Lynch syndrome** who is currently followed in a **non-structured local setting**, where colonoscopies are performed intermittently by different endoscopists and **no system is in place to monitor surveillance adherence or procedural quality**, a **more centralized and structured follow-up approach is recommended**.

This recommendation is **strong**, although it is supported by **low-quality evidence**, mainly derived from observational studies, expert consensus, and indirect data showing that **organization of care, consistency of surveillance, and quality monitoring** are critical determinants of effectiveness in Lynch syndrome surveillance. Despite the limited certainty of the evidence, guideline panels emphasize this approach because of the **high lifetime risk of colorectal cancer** in this population and the potential consequences of suboptimal surveillance.

Accordingly, it is **strongly advised** that follow-up care be **coordinated through a dedicated Lynch syndrome program or specialized center**, where:

- Colonoscopic surveillance is performed at **regular, predefined intervals**
- Examinations are conducted by **experienced endoscopists**
- **Quality indicators and adherence to surveillance schedules** are systematically monitored
- Multidisciplinary expertise is available when needed

While the supporting evidence is not based on randomized trials, the balance of potential benefit versus harm clearly favors a structured approach in this high-risk population. Implementation should nevertheless consider **local resources and patient preferences**, consistent with the quality of evidence underlying this recommendation.

Case 3.2

Vignette
A 26-year-old asymptomatic individual is known to carry a pathogenic MLH1 mutation and has never undergone colonoscopic evaluation.

Question
What would be the most appropriate approach to colorectal surveillance at this stage? In your answer, explicitly take into account the strength of the recommendation and the quality of the evidence supporting it, and calibrate the wording accordingly.

Most appropriate management: immediate initiation of colonoscopic surveillance

In a **26-year-old asymptomatic individual with a confirmed pathogenic MLH1 mutation** who has **never undergone colonoscopic evaluation**, **initiation of colonoscopic surveillance is recommended**.

This recommendation is **strong** and supported by **moderate-quality evidence**, based on cohort studies and long-term observational data showing that individuals with **MLH1-associated Lynch syndrome** have a **substantially increased lifetime risk of colorectal cancer**, with cancers occurring at a younger age compared with the general population. Although randomized trial evidence is lacking, the consistency of available data underpins a clear benefit–risk balance in favor of early surveillance.

Accordingly, **colonoscopy should be started at this age and performed at regular intervals**, typically **every 1 to 2 years**, in line with guideline recommendations for MLH1 mutation carriers. The goal is early detection and removal of premalignant lesions and early-stage cancers, thereby reducing colorectal cancer incidence and mortality.

While the recommendation is strong, follow-up care should still be **delivered within a structured surveillance program**and take into account individual factors such as comorbidities, patient preferences, and access to experienced endoscopists, consistent with the quality of evidence supporting this approach.

Case 3.3

Vignette
A 38-year-old asymptomatic individual with a confirmed diagnosis of Lynch syndrome is enrolled in a surveillance program and has been undergoing regular follow-up.

Question
How should colonoscopic surveillance be scheduled for this individual? In your answer, explicitly take into account the strength of the recommendation and the quality of the evidence supporting it, and calibrate the wording accordingly.

Most appropriate management: continue regular, high-quality colonoscopic surveillance

In a **38-year-old asymptomatic individual with a confirmed diagnosis of Lynch syndrome** who is already **enrolled in a structured surveillance program**, **continuation of regular colonoscopic surveillance is recommended**.

This recommendation is **strong** and supported by **moderate-quality evidence**, derived mainly from cohort studies and long-term observational data indicating that **regular colonoscopy reduces colorectal cancer incidence and mortality** in individuals with Lynch syndrome, despite the absence of randomized trials. The elevated and persistent lifetime risk of colorectal cancer in this population justifies a firm recommendation for ongoing surveillance.

Accordingly, **colonoscopy should be continued at regular intervals**, typically **every 1 to 2 years**, using **high-quality examinations performed by experienced endoscopists**, as part of an organized program that ensures adherence to surveillance schedules and monitoring of quality indicators.

While the evidence base is not high-certainty, there is broad consensus that **interrupting or spacing surveillance beyond recommended intervals may increase the risk of interval cancers**. Surveillance should therefore be maintained consistently over time, while remaining **individualized** according to patient age, comorbidities, prior findings, and preferences, in keeping with the level of evidence supporting this strategy.

Case 3.4

Vignette
An individual with Lynch syndrome undergoes a surveillance colonoscopy that is incomplete because of poor bowel preparation.

Question
How should follow-up be managed after this examination?
In your answer, explicitly take into account the strength of the recommendation and the quality of the evidence supporting it, and calibrate the wording accordingly.

Most appropriate management: prompt repeat high-quality colonoscopy after an incomplete examination

In an individual with **Lynch syndrome** whose **surveillance colonoscopy is incomplete due to poor bowel preparation**, a **prompt repeat colonoscopy is recommended** to ensure an adequate evaluation of the colon.

This is a **strong recommendation** supported by **moderate-quality evidence**, largely based on observational data and expert consensus showing that **incomplete examinations are associated with missed advanced neoplasia**, and that this risk is particularly relevant in Lynch syndrome given the **high incidence and rapid progression of colorectal neoplasia**. Although randomized trials are lacking, the potential harm of delayed or incomplete assessment outweighs the burden of repeating the procedure.

Accordingly, the colonoscopy **should be repeated as soon as feasible**, ideally **within a short time frame (for example, within a few months)**, using an **optimized bowel preparation regimen** and ensuring high procedural quality. The goal is to achieve a **complete, high-quality examination** that restores the patient to the intended surveillance schedule.

While the recommendation is strong, practical aspects such as patient tolerance, comorbidities, and adherence to bowel preparation should be addressed to minimize the risk of repeat inadequacy. Once a complete examination is achieved, **regular Lynch syndrome surveillance intervals should be resumed**, consistent with the level of evidence supporting this approach.

Case 3.5

Vignette
An individual with Lynch syndrome who is enrolled in a surveillance program develops new gastrointestinal symptoms between scheduled examinations.

Question
What would be the most appropriate next step in management?
In your answer, explicitly take into account the strength of the recommendation and the quality of the evidence supporting it, and calibrate the wording accordingly.

Most appropriate management: prompt diagnostic evaluation outside the routine surveillance schedule

In an individual with **Lynch syndrome** who is **enrolled in a surveillance program** and develops **new gastrointestinal symptoms between scheduled examinations**, **prompt diagnostic evaluation is recommended**, rather than waiting for the next planned surveillance colonoscopy.

This is a **strong recommendation**, supported by **low- to moderate-quality evidence**, based mainly on observational studies and expert consensus. Although high-certainty trial data are lacking, guidelines emphasize that **new symptoms may signal interval neoplasia or other clinically relevant pathology**, and that delaying evaluation in this high-risk population could result in missed or advanced disease.

Accordingly, it is **appropriate to perform a timely diagnostic work-up**, which will usually include **expedited colonoscopy**, tailored to the nature and severity of symptoms (for example, rectal bleeding, iron-deficiency anemia, unexplained abdominal pain, or change in bowel habits). Additional investigations may be considered as clinically indicated.

While routine surveillance intervals remain important, **symptom-driven assessment takes precedence** in this context. After diagnostic evaluation and management of any findings, the patient should then be **reintegrated into the regular surveillance program**, with follow-up intervals adjusted as needed based on results and overall clinical judgment.

Case 3.6

Vignette
A patient with Lynch syndrome undergoes colonoscopic surveillance using a standard-definition endoscopy system.

Question
What considerations should guide the choice of endoscopic equipment for future surveillance?
In your answer, explicitly take into account the strength of the recommendation and the quality of the evidence supporting it, and calibrate the wording accordingly.

Most appropriate management: optimize colonoscopic surveillance using high-definition endoscopy

In a patient with **Lynch syndrome** who undergoes surveillance colonoscopy using a **standard-definition endoscopy system**, **optimization of endoscopic technique is recommended** to improve lesion detection.

This is a **strong recommendation** supported by **high-quality evidence**, based on comparative studies demonstrating that **high-definition white-light endoscopy** improves visualization and detection of subtle colorectal lesions compared with standard-definition systems. Given the **elevated risk of interval cancers** in Lynch syndrome, maximizing diagnostic performance is considered particularly important.

Accordingly, surveillance colonoscopies **should preferably be performed using high-definition endoscopy systems**, ideally by experienced endoscopists within a structured surveillance program. Upgrading the imaging platform is expected to enhance mucosal assessment without adding procedural risk.

While the recommendation is strong, implementation may depend on **local availability and resources**. If immediate access to high-definition equipment is limited, efforts should be made to **transition future surveillance examinations** to high-definition systems as soon as feasible, consistent with the quality of evidence supporting this approach and recommendations from organizations such as the European Society of Gastrointestinal Endoscopy.

Case 3.7

Vignette
An asymptomatic individual with Lynch syndrome asks whether the use of dye-based techniques during colonoscopy could improve lesion detection.

Question
How should this option be considered in planning surveillance?
In your answer, explicitly take into account the strength of the recommendation and the quality of the evidence supporting it, and calibrate the wording accordingly.

Most appropriate management: consider dye-based chromoendoscopy as an adjunct, without routine use

In an **asymptomatic individual with Lynch syndrome** who asks whether **dye-based techniques** could improve lesion detection during colonoscopy, **dye-based chromoendoscopy may be considered**, but it is **not routinely recommended for all examinations**.

This corresponds to a **weak recommendation** supported by **moderate-quality evidence**. Comparative studies suggest that dye-based chromoendoscopy can modestly increase detection of some lesions compared with standard white-light endoscopy; however, results are inconsistent, benefits are operator- and setting-dependent, and high-quality colonoscopy using modern imaging already achieves good detection in many programs. As a result, guideline panels frame this option cautiously.

Accordingly, it is **reasonable to discuss chromoendoscopy as an adjunctive option** in selected circumstances, such as:

- prior examinations with suboptimal detection,
- particularly challenging mucosal assessment,
- availability of expertise without compromising procedure quality or duration.

At the same time, **routine use for all Lynch syndrome surveillance is not required**, provided that colonoscopies are performed at appropriate intervals, with **high-definition imaging and careful technique**.

Given the conditional nature of the evidence, the decision **should be individualized**, incorporating patient preferences, local expertise, and procedural feasibility, consistent with guidance from organizations such as the European Society of Gastrointestinal Endoscopy.

Case 3.8

Vignette
An asymptomatic patient with Lynch syndrome asks whether regular upper gastrointestinal endoscopic examinations are necessary as part of routine follow-up.

Question
How should this issue be addressed?
In your answer, explicitly take into account the strength of the recommendation and the quality of the evidence supporting it, and calibrate the wording accordingly.

Most appropriate management: do not perform routine upper gastrointestinal endoscopic surveillance

In an **asymptomatic patient with Lynch syndrome**, **routine upper gastrointestinal (UGI) endoscopic surveillance is generally not recommended** as part of standard follow-up.

This is a **strong recommendation** supported by **low-quality evidence**, based mainly on observational studies and expert consensus indicating that, in unselected asymptomatic individuals with Lynch syndrome, the **incidence of clinically relevant upper GI neoplasia is low**, and routine endoscopic surveillance has **not been shown to provide a clear clinical benefit**. Despite the limited certainty of the evidence, guideline panels have issued a strong recommendation because the balance of benefits and harms does not favor routine testing.

Accordingly, **scheduled UGI endoscopy should not be included in routine surveillance** for asymptomatic patients. Upper endoscopy may be **considered only in selected circumstances**, such as:

- the presence of **upper GI symptoms**,
- **specific additional risk factors** (for example, strong family history of gastric cancer),
- or within **research protocols** or tailored strategies in high-incidence regions.

Given the low quality of the supporting evidence, this recommendation should be **applied with clinical judgment**, but in the absence of symptoms or additional risk modifiers, **routine UGI surveillance is not indicated**, in line with guidance from organizations such as the European Society of Gastrointestinal Endoscopy.

Case 3.9

Vignette
An individual with Lynch syndrome asks whether testing for *Helicobacter pylori* should be performed despite the absence of gastrointestinal symptoms.

Question
What would be the most appropriate advice in this situation?
In your answer, explicitly take into account the strength of the recommendation and the quality of the evidence supporting it, and calibrate the wording accordingly.

Most appropriate management: consider non-invasive *Helicobacter pylori* testing, without making it routine

In an **asymptomatic individual with Lynch syndrome** who asks whether **testing for *Helicobacter pylori*** should be performed, **non-invasive testing may be considered**, but it is **not mandatory** in all patients.

This corresponds to a **weak recommendation** supported by **moderate-quality evidence**. Observational data suggest a potential association between *H. pylori* infection and gastric cancer risk, including in Lynch syndrome, but direct evidence demonstrating a clear clinical benefit of universal testing and eradication in asymptomatic carriers is limited. For this reason, guideline panels intentionally frame this recommendation in conditional terms.

Accordingly, it is **reasonable to offer non-invasive *H. pylori* testing** (such as urea breath test or stool antigen test), particularly in:

- regions with **high *H. pylori* prevalence or gastric cancer incidence**, or
- individuals with **additional risk factors** (for example, family history of gastric cancer).

However, **routine testing in all asymptomatic individuals with Lynch syndrome is not required**, and the decision should be **individualized**, taking into account epidemiological context, patient preferences, and local practice patterns.

Given the uncertainty of the evidence, shared decision-making is appropriate, consistent with guidance from organizations such as the European Society of Gastrointestinal Endoscopy, which emphasize a cautious, context-dependent approach rather than universal testing.

Case 3.10

Vignette
An asymptomatic individual with Lynch syndrome asks whether additional investigations of the small bowel should be routinely included in surveillance.

Question
What would be the most appropriate management approach?
In your answer, explicitly take into account the strength of the recommendation and the quality of the evidence supporting it, and calibrate the wording accordingly.

Most appropriate management: do not perform routine small-bowel surveillance

In an **asymptomatic individual with Lynch syndrome**, **routine investigation of the small bowel is generally not recommended** as part of standard surveillance.

This is a **strong recommendation** supported by **moderate-quality evidence**, based on observational studies and expert consensus showing that the **incidence of clinically relevant small-bowel neoplasia is low**, and that routine surveillance has **not demonstrated a clear benefit** in asymptomatic carriers. Despite the absence of randomized trials, guideline panels have issued a strong recommendation because the balance of potential benefit versus burden and downstream harms does not favor routine testing.

Accordingly, **systematic small-bowel investigations** (such as capsule endoscopy, CT or MR enterography) **should not be included in routine follow-up** for asymptomatic individuals. Small-bowel evaluation may be **considered selectively**in the presence of:

- **suggestive symptoms** (for example, unexplained iron-deficiency anemia, occult bleeding, or abdominal pain),
- **specific clinical indications**, or
- **research settings**.

In practice, surveillance should prioritize **high-quality colonoscopy at appropriate intervals**, while reserving small-bowel investigations for **symptom-driven or individualized indications**, consistent with guidance from organizations such as the European Society of Gastrointestinal Endoscopy.

Case 3.11

Vignette
A 45-year-old individual reports a family history of colorectal cancer affecting two first-degree relatives, one of whom was diagnosed before the age of 50 years.

Question
How should colorectal cancer risk be classified in this case?
In your answer, explicitly take into account the strength of the recommendation and the quality of the evidence supporting it, and calibrate the wording accordingly.

Most appropriate management: classify as increased familial risk and initiate intensified colonoscopic surveillance

In a **45-year-old individual with a family history of colorectal cancer affecting two first-degree relatives**, **one diagnosed before age 50**, the clinical scenario is consistent with **increased familial colorectal cancer risk**. An **intensified colonoscopic surveillance strategy is recommended**.

This is a **strong recommendation** supported by **moderate-quality evidence**, based on cohort studies and consistent guideline consensus showing that individuals with this familial pattern have a **substantially higher risk of colorectal cancer** compared with the average-risk population. Although randomized trials are not available, the observed risk gradient and age-related penetrance support earlier and more frequent surveillance.

Accordingly, **colonoscopy should be initiated now** (if not already performed) and **repeated at regular intervals**, typically **every 5 years**, provided examinations are of high quality and findings are negative. This approach aims to detect advanced neoplasia at an early, treatable stage.

While the recommendation is strong, surveillance should still be **delivered within a structured program** and **individualized** based on additional factors such as the exact age at diagnosis of affected relatives, number of relatives involved, patient comorbidities, and preferences. Management should also consider referral for **genetic counseling** if features suggest a hereditary cancer syndrome, in line with guidance from organizations such as the European Society of Gastrointestinal Endoscopy.

Case 3.12

Vignette
A first-degree relative of a patient with colorectal cancer belongs to a family that meets criteria for increased familial risk.

Question
What would be the most appropriate colorectal surveillance strategy for this individual? In your answer, explicitly take into account the strength of the recommendation and the quality of the evidence supporting it, and calibrate the wording accordingly.

Most appropriate management: initiate and maintain intensified colonoscopic surveillance

In a **first-degree relative of a patient with colorectal cancer** who belongs to a family meeting criteria for **increased familial colorectal cancer risk**, **intensified colonoscopic surveillance is recommended**.

This is a **strong recommendation** supported by **moderate-quality evidence**, based on cohort studies and consistent guideline consensus showing that first-degree relatives in such families have a **significantly higher risk of colorectal cancer** compared with the average-risk population. Although randomized trial evidence is lacking, the magnitude and consistency of the observed risk justify a firm recommendation for proactive surveillance.

Accordingly, **colonoscopy should be initiated earlier than in average-risk individuals** and **repeated at regular intervals**, typically **every 5 years**, provided that examinations are complete and of high quality and no lesions requiring shorter intervals are detected. This strategy aims to reduce colorectal cancer incidence and mortality through earlier detection and removal of precancerous lesions.

While the recommendation is strong, follow-up care should remain **structured and individualized**, taking into account the exact family history (number of affected relatives and age at diagnosis), patient age, comorbidities, and preferences. Referral for **genetic counseling** may be appropriate if additional features suggest a hereditary cancer syndrome, in line with guidance from organizations such as the European Society of Gastrointestinal Endoscopy.

Case 3.13

Vignette
A 41-year-old individual with an established familial risk of colorectal cancer has not previously undergone colonoscopy.

Question
When should colorectal surveillance be initiated?
In your answer, explicitly take into account the strength of the recommendation and the quality of the evidence supporting it, and calibrate the wording accordingly.

Most appropriate management: initiate colonoscopic surveillance without further delay

In a **41-year-old individual with an established familial risk of colorectal cancer** who has **not previously undergone colonoscopy**, **initiation of colonoscopic surveillance is recommended**.

This is a **strong recommendation** supported by **moderate-quality evidence**, based on cohort studies and guideline consensus showing that individuals with familial colorectal cancer risk have a **higher incidence of colorectal neoplasia**and benefit from **earlier and proactive surveillance** compared with average-risk populations. Although randomized trials are lacking, the consistency of observational data supports a clear benefit–risk balance in favor of starting surveillance.

Accordingly, **a baseline colonoscopy should be performed now**. If the examination is complete and of high quality and no lesions requiring shorter intervals are detected, **subsequent colonoscopies are typically recommended at 5-year intervals**, in line with guidance for familial-risk groups.

While the recommendation is strong, follow-up care should be **structured and individualized**, taking into account the number of affected relatives, age at diagnosis in the family, patient comorbidities, and preferences. Consideration of **genetic counseling** may also be appropriate if the family history suggests an inherited cancer syndrome, consistent with recommendations from organizations such as the European Society of Gastrointestinal Endoscopy.

Case 3.14

Vignette
An individual with familial risk of colorectal cancer undergoes a high-quality baseline colonoscopy that shows no neoplastic lesions.

Question
How should future surveillance be planned?
In your answer, explicitly take into account the strength of the recommendation and the quality of the evidence supporting it, and calibrate the wording accordingly.

Most appropriate management: continue scheduled colonoscopic surveillance at extended intervals

In an individual with **familial risk of colorectal cancer** who has undergone a **high-quality baseline colonoscopy**showing **no neoplastic lesions**, **continued colonoscopic surveillance at extended intervals is recommended**.

This represents a **strong recommendation** supported by **low- to moderate-quality evidence**, based mainly on cohort studies and expert consensus indicating that, although the baseline examination is negative, individuals with familial risk remain at **higher long-term risk** of colorectal cancer than the average-risk population. While direct randomized evidence is limited, the consistency of observational data supports ongoing surveillance rather than discharge to average-risk screening.

Accordingly, **repeat colonoscopy is generally recommended at 5-year intervals**, provided that the baseline examination was complete and of high quality and no new risk factors emerge. This interval aims to balance the benefit of early detection against the risks and burden of unnecessary overuse.

It should be acknowledged that the supporting evidence is not high certainty. Therefore, surveillance intervals **may be individualized** based on patient age, comorbidities, exact family history (number of affected relatives and age at diagnosis), and patient preferences, in line with guidance from organizations such as the European Society of Gastrointestinal Endoscopy.

Case 3.15

Vignette
A patient with familial risk of colorectal cancer undergoes colonoscopy with removal of one or more adenomas.

Question
How should follow-up after polyp removal be managed?
In your answer, explicitly take into account the strength of the recommendation and the quality of the evidence supporting it, and calibrate the wording accordingly.

Most appropriate management: base follow-up on post-polypectomy findings rather than familial risk alone

In a patient with **familial risk of colorectal cancer** who undergoes colonoscopy with **removal of one or more adenomas**, subsequent follow-up **should be determined by the characteristics of the removed adenomas**, rather than by familial risk per se.

This approach reflects a **strong recommendation** supported by **moderate-quality evidence**, based on cohort studies and guideline consensus showing that **the risk of metachronous neoplasia is primarily driven by the most recent endoscopic findings** (number, size, histology, and presence of dysplasia), and that applying standard post-polypectomy surveillance rules is appropriate even in individuals with familial risk.

Accordingly, **surveillance intervals should follow established post-polypectomy recommendations**, for example:

- **Low-risk adenomas** (few, small, non-advanced): longer intervals or return to screening, depending on local screening availability.
- **Advanced adenomas or multiple adenomas**: shorter surveillance intervals (commonly around **3 years**), as indicated by lesion risk.

Familial risk does **not** in itself mandate shortening the interval beyond what is recommended for the adenoma profile. However, surveillance should be **individualized** in selected cases, considering examination quality, patient age and comorbidities, and preferences.

This strategy aligns with guidance from organizations such as the European Society of Gastrointestinal Endoscopy, which emphasize that **post-polypectomy findings should take precedence** when planning follow-up, even in patients with increased familial colorectal cancer risk.

Guideline 4. Colorectal polypectomy and endoscopic mucosal resection

Case 4.1

Vignette
During colonoscopy, a flat 25 mm nonpedunculated colorectal lesion is identified. The endoscopist evaluates the lesion using white-light endoscopy and virtual chromoendoscopy, assessing pit and vascular patterns together with macroscopic features.

Question
How should optical diagnosis be performed and integrated to estimate the risk of submucosal invasion in this lesion?
In your answer, explicitly take into account the strength of the recommendation and the quality of the evidence supporting it, and calibrate the wording accordingly.

Most appropriate management: integrated optical diagnosis using a structured, multimodal approach

For a **flat 25 mm nonpedunculated colorectal lesion**, **optical diagnosis should be performed using a structured integration of macroscopic morphology, pit pattern, and vascular pattern analysis**, combining **white-light endoscopy** and **virtual chromoendoscopy** to estimate the risk of submucosal invasion.

This approach reflects a **strong recommendation**, supported by **moderate-quality evidence**, derived from prospective observational studies, diagnostic accuracy studies, and expert consensus. These data consistently show that **no single endoscopic feature is sufficiently reliable on its own**, whereas a **combined assessment** improves risk stratification for deep submucosal invasion.

Accordingly, optical diagnosis **should usually include**:

- **Macroscopic assessment** (Paris classification, lesion size, surface morphology, presence of depression, ulceration, or stiffness), which provides an initial estimate of invasion risk.
- **Pit pattern analysis** (for example, Kudo classification) under magnification or enhanced imaging, recognizing that irregular or non-structured patterns are associated with higher invasion risk.
- **Vascular pattern analysis** using virtual chromoendoscopy (such as NBI-based classifications), where disrupted, irregular, or avascular patterns suggest a higher likelihood of deep submucosal invasion.

When these features are **concordant and suggest superficial disease**, endoscopic resection can generally be considered appropriate. Conversely, **features suggestive of deep submucosal invasion** should prompt caution, as they are associated with a higher risk of non-curative endoscopic resection and lymph node metastasis.

It is important to emphasize that, although the recommendation to integrate multiple optical criteria is strong, **the predictive accuracy is not absolute**. Therefore, optical diagnosis should be **used to guide management decisions rather than to replace histopathological assessment**, and uncertainty should be explicitly acknowledged when features are borderline or discordant.

Overall, a **systematic, multimodal optical diagnosis**, performed by trained endoscopists and interpreted within the limits of available evidence, represents the most appropriate strategy to estimate submucosal invasion risk in large nonpedunculated colorectal lesions.

Case 4.2

Vignette
A 15 mm laterally spreading colorectal lesion is detected during screening colonoscopy.

Question
How should this polyp be described and reported in the endoscopy report according to guideline recommendations?
In your answer, explicitly take into account the strength of the recommendation and the quality of the evidence supporting it, and calibrate the wording accordingly.

Most appropriate management: standardized, structured description in the endoscopy report

For a **15 mm laterally spreading colorectal lesion (LST)** detected at screening colonoscopy, the endoscopy report **should use a standardized, structured description** to support correct risk stratification, resection planning, and referral when needed.

This is generally a **strong recommendation** supported by **moderate-quality evidence**, based on diagnostic accuracy studies and broad guideline consensus showing that structured reporting improves interobserver agreement, appropriateness of resection strategy, and continuity of care.

Accordingly, the lesion **should usually be reported** with the following minimum elements:

- **Location**: segment (cecum, ascending, transverse, descending, sigmoid, rectum) and distance from the anal verge for rectal lesions.
- **Size**: estimated in **mm**, ideally with a reference (open snare, forceps).
- **Morphology**:
  - **Paris classification** (e.g., 0-IIa, 0-IIa+Is).
  - **LST subtype**: granular (LST-G: homogeneous or nodular mixed) vs non-granular (LST-NG: flat elevated or pseudodepressed), because subtype informs invasion risk and technique.
- **Surface and suspicious features for submucosal invasion** (describe presence/absence of):
  - depression, ulceration, demarcated nodule, stiffness, fold convergence, non-lifting (if tested), spontaneous bleeding, marked irregularity.
- **Optical diagnosis using enhanced imaging** (state the system used and classification applied):
  - e.g., NICE/JNET (or equivalent) and, where applicable, **pit pattern** assessment, with an explicit statement on **suspected depth** (no suspicion, possible superficial invasion, suspicion of deep invasion).
- **Resection plan and rationale**:
  - anticipated approach (cold snare, hot snare EMR, en bloc EMR, ESD, referral), including why (size, morphology, optical diagnosis, access).
- **Photo documentation**:
  - representative images in white light and enhanced imaging.
- **Tattooing and marking** (if relevant):
  - whether tattoo was placed, **where** (typically distal to lesion), and whether the lesion was left in situ for referral.

Because the evidence is not perfect and optical diagnosis is operator-dependent, the report should also **clearly state any uncertainty** (for example, “features borderline for superficial invasion”), which helps avoid overconfident management decisions while remaining aligned with guideline intent.

Case 4.3

Vignette
A patient presents with a 30 mm nonpedunculated colorectal polyp scheduled for endoscopic resection.

Question
Which endoscopic imaging modalities should be used before treatment to assess the presence and depth of submucosal invasion?
In your answer, explicitly take into account the strength of the recommendation and the quality of the evidence supporting it, and calibrate the wording accordingly.

Most appropriate management: multimodal optical assessment using white-light and image-enhanced endoscopy

In a patient with a **30 mm nonpedunculated colorectal polyp** scheduled for endoscopic resection, **pre-treatment assessment of submucosal invasion should be based on a systematic optical evaluation using high-quality white-light endoscopy combined with image-enhanced modalities**.

This represents a **strong recommendation** supported by **moderate-quality evidence**, derived from prospective observational studies, diagnostic accuracy studies, and expert consensus showing that **no single imaging modality is sufficient on its own**, whereas a **multimodal approach improves the ability to identify features suggestive of deep submucosal invasion**.

Accordingly, the evaluation **should usually include**:

- **High-definition white-light endoscopy** for assessment of overall morphology, size, surface topography, and macroscopic signs of invasion (depression, ulceration, fold convergence, stiffness).
- **Virtual chromoendoscopy** (for example, NBI, BLI, or i-scan) to analyze **vascular and surface patterns** using validated classifications, helping to stratify the likelihood of superficial versus deep invasion.
- **Magnification, when available** to support **pit pattern and microvascular assessment**, recognizing that irregular or non-structured patterns are associated with higher invasion risk.

When these modalities show **concordant features compatible with superficial disease**, endoscopic resection can generally proceed. Conversely, **optical features suggestive of deep submucosal invasion** should prompt caution and reconsideration of the resection strategy, including referral for surgery or en bloc resection only in highly selected cases.

It is important to note that, although the recommendation to use multimodal imaging is strong, **the certainty of evidence is not absolute**. Optical diagnosis **guides but does not replace histopathology**, and borderline or discordant findings should be explicitly acknowledged in the report to avoid overconfident management decisions.

Case 4.4

Vignette
Advanced endoscopic imaging of a large colorectal lesion shows features suggestive of deep submucosal invasion.

Question
What is the most appropriate next step in management before proceeding with treatment? In your answer, explicitly take into account the strength of the recommendation and the quality of the evidence supporting it, and calibrate the wording accordingly.

Most appropriate management: avoid endoscopic resection and refer for multidisciplinary oncologic evaluation

When **advanced endoscopic imaging of a large colorectal lesion shows features suggestive of deep submucosal invasion**, **endoscopic resection should generally not be pursued as definitive treatment**.

This reflects a **strong recommendation**, supported by **moderate-quality evidence**, derived from diagnostic accuracy studies, surgical pathology series, and guideline consensus demonstrating that **deep submucosal invasion is associated with a substantial risk of lymph node metastasis** and a high likelihood of **non-curative endoscopic resection**.

Accordingly, the most appropriate next step is to **refer the patient for multidisciplinary evaluation**, typically involving colorectal surgery, oncology, pathology, and advanced endoscopy, to plan **oncologic surgical resection** with appropriate lymphadenectomy. Additional staging investigations may be arranged as part of this process, according to local protocols.

Although the evidence base is not derived from randomized trials, the consistency of observational data supports a clear benefit–risk balance in favor of **surgical management** in this setting. Attempting endoscopic resection despite convincing features of deep invasion risks incomplete treatment and delays definitive therapy.

In cases where imaging features are **borderline or discordant**, further expert review or repeat high-quality assessment may be reasonable. However, when **clear optical signs of deep submucosal invasion are present**, **referral for surgery rather than endoscopic therapy is the recommended course of action**.

Case 4.5

Vignette
Several colorectal polyps are resected during colonoscopy. The endoscopist considers discarding diminutive polyps without histologic assessment.

Question
What is the recommended approach regarding retrieval and histopathologic analysis of resected polyps?
In your answer, explicitly take into account the strength of the recommendation and the quality of the evidence supporting it, and calibrate the wording accordingly.

Most appropriate management: routine retrieval and histopathologic assessment, with limited, conditional exceptions

After colonoscopic resection of **multiple colorectal polyps**, **routine retrieval and histopathologic analysis of resected polyps is recommended** as the standard approach.

This represents a **strong recommendation** supported by **moderate-quality evidence**, based on observational studies and guideline consensus showing that histopathologic assessment remains essential for **accurate diagnosis, risk stratification, and determination of appropriate surveillance intervals**. In routine practice, discarding resected polyps without histology risks misclassification of lesion type and subsequent follow-up errors.

Accordingly, **all resected polyps should generally be retrieved and sent for histopathologic examination**.

A **limited exception** may be considered for **diminutive (≤5 mm) polyps in the rectosigmoid colon**, where a **“resect-and-discard” or “diagnose-and-leave” strategy** can be applied **only under strict conditions**:

- use of **validated optical diagnosis criteria**,
- **high confidence** endoscopic characterization by trained endoscopists,
- **documented performance thresholds** (accuracy, negative predictive value),
- and **local protocols** ensuring correct assignment of surveillance intervals.

This exception corresponds to a **weak recommendation** supported by **low- to moderate-quality evidence**, as real-world performance outside expert centers is variable and implementation requires robust quality assurance systems.

In the absence of such validated systems and documented expertise, **discarding polyps without histologic confirmation is not advised**. Therefore, for most settings and patients, **systematic retrieval and histopathologic analysis remains the recommended and safest strategy**, consistent with the current level of evidence.

Case 4.6

Vignette
A 40 mm nonpedunculated colorectal lesion is considered for referral to a tertiary center. Biopsies have not yet been performed.

Question
Should biopsies or photo- and videodocumentation be preferred to determine the most advanced pathology?
In your answer, explicitly take into account the strength of the recommendation and the quality of the evidence supporting it, and calibrate the wording accordingly.

Most appropriate management: prioritize high-quality photo- and videodocumentation over routine biopsies

For a **40 mm nonpedunculated colorectal lesion** being considered for **referral to a tertiary center**, **high-quality photo- and videodocumentation should be preferred over routine pre-resection biopsies** to characterize the most advanced pathology.

This represents a **strong recommendation** supported by **moderate-quality evidence**, based on observational studies, procedural series, and guideline consensus indicating that **optical assessment with advanced imaging provides more reliable information on invasion risk** and that **biopsies may compromise subsequent endoscopic resection** by inducing fibrosis and non-lifting.

Accordingly, the recommended approach is to:

- **Obtain comprehensive photo- and video documentation** using **high-definition white-light endoscopy** and **image-enhanced modalities** (virtual chromoendoscopy), capturing:
  - overall morphology and size,
  - surface and vascular patterns,
  - focal areas suspicious for deep submucosal invasion.
- **Document optical features and classifications** used to estimate invasion depth, and clearly state the level of confidence and any uncertainty.
- **Avoid routine biopsies** before referral when endoscopic resection is being considered.

Biopsies **may be considered selectively** when:

- **optical features strongly suggest deep submucosal invasion** and surgical management is likely regardless of endoscopic resection, or
- histologic confirmation is required to guide non-endoscopic management.

However, even in these situations, biopsies should be **targeted and limited**, recognizing that the supporting evidence is not absolute.

Overall, prioritizing detailed photo- and videodocumentation preserves resection options, facilitates expert review at tertiary centers, and aligns with current guideline intent, while acknowledging the **moderate certainty of the evidence**underpinning this recommendation.

Case 4.7

Vignette
A diminutive rectosigmoid polyp (4 mm) is detected and appears nonadenomatous on high-confidence optical diagnosis.

Question
Should this polyp be resected or left in situ?
In your answer, explicitly take into account the strength of the recommendation and the quality of the evidence supporting it, and calibrate the wording accordingly.

Most appropriate management: consider leaving the polyp in situ under strict conditions

For a **diminutive (4 mm) rectosigmoid polyp** that **appears nonadenomatous** on **high-confidence optical diagnosis**, **leaving the polyp in situ may be considered**, rather than routine resection.

This corresponds to a **weak recommendation** supported by **moderate-quality evidence**, derived from diagnostic accuracy studies and guideline consensus indicating that, when optical diagnosis is performed with **high confidence by trained endoscopists using validated criteria**, the risk of clinically relevant pathology in such lesions is very low. However, real-world performance varies, and the certainty of benefit is not absolute.

Accordingly, a **"diagnose-and-leave" strategy can be acceptable only if all of the following conditions are met**:

- **High-confidence optical diagnosis** using validated classifications and image-enhanced endoscopy.
- **Appropriate setting and expertise**, with documented performance metrics meeting recommended thresholds.
- **Clear documentation** of the optical diagnosis and confidence level in the endoscopy report.
- **Local protocols** ensuring correct surveillance assignment despite the absence of histology.

If any of these conditions are **not** fulfilled, **resection remains the preferred approach**, as it provides histologic confirmation and avoids misclassification.

In summary, while leaving a diminutive rectosigmoid polyp in situ is **an option in selected, well-controlled settings**, routine application is **not recommended**, and the decision should be **individualized**, reflecting the **conditional nature of the evidence** and patient- and system-level factors.

Case 4.8

Vignette
A pedunculated colorectal polyp is identified during colonoscopy.

Question
What is the recommended resection technique for this type of lesion? In your answer, explicitly take into account the strength of the recommendation and the quality of the evidence supporting it, and calibrate the wording accordingly.

Most appropriate management: snare polypectomy as the standard resection technique

For a **pedunculated colorectal polyp**, **snare polypectomy is the recommended resection technique**.

This is a **strong recommendation** supported by **moderate- to high-quality evidence**, based on comparative studies, large procedural series, and consistent guideline consensus showing that snare resection achieves **high rates of complete removal** with an **acceptable safety profile** for pedunculated lesions.

Accordingly, **hot snare polypectomy** is **generally preferred**, as it allows controlled transection of the stalk with effective hemostasis. In lesions with **large heads or thick stalks**, **prophylactic measures to reduce bleeding risk** (such as diluted adrenaline injection into the stalk, placement of a detachable loop, or clips) **should be considered**, recognizing that evidence supports bleeding reduction but practice should be individualized.

Cold snare polypectomy may be **considered for small pedunculated polyps with thin stalks**, but the supporting evidence is more limited; therefore, this option should be applied selectively and with caution.

Overall, **snare polypectomy remains the standard approach** for pedunculated colorectal polyps, with technique and adjunctive hemostatic measures tailored to **polyp size, stalk characteristics, bleeding risk, and operator expertise**, consistent with the strength of the recommendation and the quality of available evidence.

Case 4.9

Vignette
A pedunculated polyp with a 2.5 cm head and a thick stalk is scheduled for endoscopic removal.

Question
What preventive measures should be considered to reduce the risk of immediate post-polypectomy bleeding?
In your answer, explicitly take into account the strength of the recommendation and the quality of the evidence supporting it, and calibrate the wording accordingly.

Most appropriate management: apply prophylactic hemostatic measures before snare transection

For a **pedunculated polyp with a large head (2.5 cm) and a thick stalk**, **prophylactic measures to reduce the risk of immediate post-polypectomy bleeding are recommended**.

This represents a **strong recommendation** supported by **moderate-quality evidence**, based on randomized and observational studies showing that **pre-resection hemostatic interventions significantly reduce immediate bleeding** in pedunculated polyps with large heads or thick stalks. While the magnitude of benefit varies across studies, the overall balance favors prophylaxis in this high-risk setting.

Accordingly, **one or more of the following measures should be considered before snare transection**:

- **Mechanical prophylaxis of the stalk**, such as placement of a **detachable loop (endoloop)** or **hemoclips** at the base of the stalk to compress feeding vessels.
- **Injection of diluted adrenaline** into the stalk to induce vasoconstriction and reduce blood flow.

These measures may be used **alone or in combination**, depending on stalk thickness, head size, bleeding risk factors, and operator experience.

Although the recommendation is strong, the evidence does not mandate a single superior technique. Therefore, the choice of prophylactic strategy should be **individualized**, taking into account lesion characteristics, patient-related bleeding risk, and local expertise, while recognizing that **some form of prophylaxis is advisable** in this scenario to minimize immediate bleeding risk.

Case 4.10

Vignette
Multiple colonic and rectal lesions of varying size are detected during colonoscopy.

Question
Can most of these lesions be managed endoscopically with curative intent? In your answer, explicitly take into account the strength of the recommendation and the quality of the evidence supporting it, and calibrate the wording accordingly.

Most appropriate management: endoscopic treatment for most lesions, with selective exclusion based on invasion risk

When **multiple colonic and rectal lesions of varying size** are detected during colonoscopy, **most lesions can generally be managed endoscopically with curative intent**, provided that **careful optical assessment does not suggest deep submucosal invasion**.

This approach reflects a **strong recommendation** supported by **moderate-quality evidence**, based on large observational series, comparative studies, and guideline consensus showing that the vast majority of colorectal neoplastic lesions, including large nonpedunculated polyps, can be **safely and effectively treated endoscopically** when appropriately selected. Randomized data are limited, but real-world outcomes consistently support endoscopic therapy as first-line management.

Accordingly, **endoscopic resection is appropriate for most lesions**, using techniques tailored to lesion characteristics, such as cold snare polypectomy, EMR, or ESD. However, **endoscopic curative intent is not appropriate for all lesions**. Lesions showing **optical features suggestive of deep submucosal invasion** (for example, marked depression, ulceration, non-structured pit pattern, irregular or avascular vascular patterns) should **generally not be treated endoscopically**, because of the higher risk of non-curative resection and lymph node metastasis.

Therefore, the recommended strategy is to:

- Perform **systematic optical diagnosis** for each lesion using high-quality white-light endoscopy and image-enhanced modalities.
- **Treat endoscopically** those lesions assessed as superficial.
- **Refer for multidisciplinary or surgical management** lesions with convincing features of deep invasion.

While endoscopic management is appropriate in most cases, decisions should be **individualized**, acknowledging the limits of optical diagnosis and the moderate certainty of the supporting evidence, and should consider lesion complexity, patient comorbidities, and local expertise.

Case 4.11

Vignette
A 3 mm colorectal polyp is detected during screening colonoscopy.

Question
What is the preferred technique for removal of this diminutive polyp? In your answer, explicitly take into account the strength of the recommendation and the quality of the evidence supporting it, and calibrate the wording accordingly.

Most appropriate management: cold snare polypectomy as the preferred technique

For a **3 mm diminutive colorectal polyp** detected during screening colonoscopy, **cold snare polypectomy is the preferred removal technique**.

This represents a **strong recommendation** supported by **high-quality evidence**, based on randomized trials and large comparative studies demonstrating that cold snare polypectomy achieves **higher complete resection rates** and **lower risk of adverse events** compared with cold biopsy forceps, even for very small lesions.

Accordingly, **cold snare polypectomy should usually be performed**, as it allows en bloc resection with a margin of normal mucosa, improving histologic completeness without the need for electrocautery.

Cold biopsy forceps may be **considered only in selected circumstances**, such as when a snare cannot be safely or technically positioned, but this option is associated with **higher rates of incomplete resection** and is therefore not preferred.

Overall, for diminutive colorectal polyps, **cold snare polypectomy is the standard of care**, with strong supporting evidence and a favorable benefit–risk profile.

Case 4.12

Vignette
An endoscopist plans cold snare polypectomy for a small colorectal polyp.

Question
Should a margin of normal tissue be included during resection?
In your answer, explicitly take into account the strength of the recommendation and the quality of the evidence supporting it, and calibrate the wording accordingly.

Most appropriate management: include a small margin of normal mucosa during cold snare polypectomy

When performing **cold snare polypectomy for a small colorectal polyp**, **including a small rim of normal mucosa around the lesion is recommended**.

This represents a **strong recommendation** supported by **moderate- to high-quality evidence**, based on randomized trials and comparative studies showing that inclusion of a **1–2 mm margin of normal tissue** significantly improves **complete (R0) resection rates** without increasing the risk of adverse events.

Accordingly, during cold snare polypectomy, the snare **should be positioned to capture the polyp together with a narrow margin of surrounding normal mucosa**, ensuring complete excision and reducing the likelihood of residual adenomatous tissue.

While the recommendation is strong, the precise width of the margin may be **adjusted according to polyp size, location, and technical feasibility**. The principle of including a small margin, rather than exact measurements, should guide practice, consistent with the quality of the supporting evidence.

Case 4.13

Vignette
An endoscopist considers using hot biopsy forceps to remove a 5 mm colorectal polyp.

Question
Is the use of hot biopsy forceps appropriate in this situation? In your answer, explicitly take into account the strength of the recommendation and the quality of the evidence supporting it, and calibrate the wording accordingly.

Most appropriate management: avoid hot biopsy forceps for diminutive colorectal polyps

For a **5 mm colorectal polyp**, **the use of hot biopsy forceps is not recommended**.

This reflects a **strong recommendation** supported by **high-quality evidence**, based on randomized trials, comparative studies, and large observational series showing that hot biopsy forceps are associated with **higher rates of incomplete resection**, **thermal tissue damage that compromises histopathologic assessment**, and an **increased risk of adverse events**, including delayed bleeding and deep thermal injury.

Accordingly, **hot biopsy forceps should not be used** for removal of diminutive colorectal polyps. Instead, **cold snare polypectomy** is the preferred technique, as it achieves superior complete resection rates with a more favorable safety profile. **Cold biopsy forceps** may be considered only in exceptional circumstances when snare placement is not feasible, recognizing their limitations.

Given the strength of the evidence and the consistency of guideline recommendations, **hot biopsy forceps should be avoided in routine practice** for diminutive colorectal polyps, as their risks clearly outweigh any potential benefits.

Case 4.14

Vignette
Cold biopsy forceps excision is considered for removal of a small colorectal polyp.

Question
Is this technique recommended for complete polyp resection?
In your answer, explicitly take into account the strength of the recommendation and the quality of the evidence supporting it, and calibrate the wording accordingly.

Most appropriate management: avoid cold biopsy forceps for complete polyp resection

For removal of a **small colorectal polyp**, **cold biopsy forceps excision is generally not recommended** when the goal is **complete resection**.

This reflects a **strong recommendation** supported by **high-quality evidence**, based on randomized trials and comparative studies consistently showing that cold biopsy forceps are associated with **significantly higher rates of incomplete resection** compared with cold snare polypectomy, even for diminutive lesions. Incomplete excision may lead to residual neoplasia and inappropriate surveillance intervals.

Accordingly, **cold snare polypectomy should be preferred** for small and diminutive colorectal polyps, as it allows en bloc removal with a margin of normal mucosa and provides superior histologic completeness without increasing adverse events.

Cold biopsy forceps may be **considered only in exceptional circumstances**, such as when snare placement is technically not feasible, and this limitation should be explicitly recognized. Even in these cases, the endoscopist should be aware that the likelihood of complete resection is lower.

Overall, given the strength of the recommendation and the quality of the supporting evidence, **cold biopsy forceps should not be used as the standard technique for complete polyp resection**.

Case 4.15

Vignette
A 7 mm colorectal polyp is detected during colonoscopy.

Question
What is the recommended resection technique for this lesion?
In your answer, explicitly take into account the strength of the recommendation and the quality of the evidence supporting it, and calibrate the wording accordingly.

Most appropriate management: cold snare polypectomy as the preferred technique

For a **7 mm colorectal polyp** detected during colonoscopy, **cold snare polypectomy is the recommended resection technique**.

This represents a **strong recommendation** supported by **high-quality evidence**, based on randomized trials and large comparative studies demonstrating that cold snare polypectomy achieves **high complete resection rates** with a **lower risk of adverse events** compared with hot techniques or biopsy forceps for small polyps.

Accordingly, **cold snare polypectomy should usually be performed**, allowing en bloc removal with a margin of normal mucosa and avoiding electrocautery-related complications.

Hot snare polypectomy is **not routinely indicated** for polyps of this size in the absence of specific features suggesting higher risk, as it does not offer additional benefit and may increase complication rates. Overall, cold snare polypectomy provides the most favorable balance of efficacy and safety for a 7 mm colorectal polyp, consistent with the strength of the recommendation and the quality of available evidence.

Case 4.16

Vignette
A 15 mm flat granular homogeneous adenoma is found in the right colon of a patient with significant comorbidities.

Question
What endoscopic resection strategy may be considered to reduce procedural risks? In your answer, explicitly take into account the strength of the recommendation and the quality of the evidence supporting it, and calibrate the wording accordingly.

Most appropriate management: consider cold snare piecemeal resection to reduce procedural risk

In a patient with **significant comorbidities** who is found to have a **15 mm flat granular homogeneous adenoma in the right colon**, an endoscopic strategy that **minimizes procedural risk is appropriate**.

In this context, **cold snare piecemeal resection may be considered** as an alternative to conventional hot snare EMR.

This corresponds to a **weak recommendation** supported by **low- to moderate-quality evidence**, derived mainly from prospective cohort studies and non-randomized comparative data suggesting that cold snare techniques for selected lesions up to 15–20 mm can **substantially reduce the risk of deep thermal injury, perforation, and delayed bleeding**, particularly in the thin-walled right colon. However, evidence is still limited and long-term recurrence data are less robust than for hot EMR.

Accordingly, in carefully selected cases such as **granular homogeneous laterally spreading tumors**, where the risk of deep submucosal invasion is low and complete en bloc resection is not mandatory, **cold snare piecemeal resection can be a reasonable option**, especially when reducing procedural risk is a priority.

It should be emphasized that this approach requires:

- **High-quality optical diagnosis** confirming low invasion risk,
- **Careful technique and thorough inspection** of the resection margins,
- **Planned surveillance**, recognizing that recurrence risk may be higher than with en bloc resection.

Given the limited certainty of the evidence, this strategy should be **individualized**, balancing patient comorbidities, lesion characteristics, local expertise, and the modest trade-off between safety and completeness of resection.

Case 4.17

Vignette
A 12 mm sessile serrated lesion without dysplasia is detected.

Question
What is the recommended technique for resection of this lesion? In your answer, explicitly take into account the strength of the recommendation and the quality of the evidence supporting it, and calibrate the wording accordingly.

Most appropriate management: cold snare polypectomy as the preferred technique

For a **12 mm sessile serrated lesion (SSL) without dysplasia**, **cold snare polypectomy is recommended** as the preferred resection technique.

This represents a **strong recommendation** supported by **moderate- to high-quality evidence**, based on randomized and comparative studies showing that cold snare resection of SSLs up to at least 10–15 mm achieves **high complete resection rates** with a **lower risk of adverse events**, particularly delayed bleeding and deep thermal injury, compared with hot techniques. These safety advantages are especially relevant for serrated lesions, which often have indistinct margins and occur in the proximal colon.

Accordingly, **cold snare polypectomy should usually be performed**, ensuring:

- **En bloc resection when feasible**, or careful piecemeal cold snare resection if required by lesion size or shape,
- **Inclusion of a small rim of normal mucosa** to improve completeness,
- **Careful inspection of the resection site** to confirm complete removal.

While the recommendation is strong, technical choices should remain **individualized** based on lesion morphology, location, and operator experience. **Hot snare EMR** is **not routinely indicated** for non-dysplastic SSLs of this size and should generally be reserved for selected situations where cold snare resection is not feasible.

Overall, cold snare polypectomy offers the most favorable balance of efficacy and safety for a 12 mm SSL without dysplasia, consistent with the strength of the recommendation and the quality of the available evidence.

Case 4.18

Vignette
Piecemeal cold snare polypectomy is planned for a sessile serrated lesion.

Question
Should submucosal injection be used prior to resection?
In your answer, explicitly take into account the strength of the recommendation and the quality of the evidence supporting it, and calibrate the wording accordingly.

Most appropriate management: do not routinely use submucosal injection before piecemeal cold snare resection

When **piecemeal cold snare polypectomy** is planned for a **sessile serrated lesion (SSL)**, **routine submucosal injection is generally not recommended**.

This corresponds to a **weak recommendation** supported by **low- to moderate-quality evidence**, based on observational studies and expert consensus indicating that submucosal injection **does not clearly improve completeness or safety** for cold snare resection and may, in some cases, **flatten lesion margins**, making capture with the snare more difficult. Because the evidence is limited and heterogeneous, guideline panels frame this advice cautiously.

Accordingly, **piecemeal cold snare resection is usually performed without injection**, relying on careful snare placement and inclusion of a small rim of normal mucosa to achieve complete removal.

Selective use of submucosal injection may be considered in specific circumstances, such as:

- poor lesion lifting due to fibrosis or scarring,
- difficult visualization or stability of the lesion,
- the need to improve safety margins in anatomically challenging locations.

Given the conditional nature of the evidence, the decision to inject **should be individualized**, taking into account lesion characteristics, technical difficulty, and endoscopist experience. Overall, **routine injection is not required**, but selective use can be reasonable in carefully chosen cases.

Case 4.19

Vignette
A sessile serrated lesion with a dysplastic component is identified.

Question
What is the recommended resection approach for this lesion?
In your answer, explicitly take into account the strength of the recommendation and the quality of the evidence supporting it, and calibrate the wording accordingly.

Most appropriate management: complete endoscopic resection with en bloc intent when feasible

For a **sessile serrated lesion (SSL) with a dysplastic component**, **complete endoscopic resection is recommended**, with a preference for an **en bloc resection strategy whenever technically feasible**.

This represents a **strong recommendation** supported by **moderate-quality evidence**, based on observational studies, pathological series, and guideline consensus showing that the presence of dysplasia in an SSL is associated with a **higher risk of advanced neoplasia and progression**, making **complete histologic assessment and clear margins** particularly important. Although randomized comparative trials are limited, the consistency of available data supports a more rigorous resection approach than for non-dysplastic SSLs.

Accordingly, the recommended strategy is to:

- **Aim for en bloc resection** to allow accurate histopathologic evaluation of the dysplastic component and resection margins.
- Use **hot snare EMR** or **en bloc cold snare resection** (for smaller lesions) depending on lesion size, morphology, location, and endoscopist expertise.
- **Avoid piecemeal cold snare resection** when en bloc removal is achievable, because fragmentation may compromise margin assessment and histologic interpretation.

If en bloc resection is **not technically feasible** because of lesion size or location, **piecemeal EMR may be performed**, recognizing that this approach requires **careful site assessment and planned surveillance** due to a higher risk of residual or recurrent tissue.

Overall, because dysplasia increases the clinical relevance of SSLs, management should prioritize **complete, well-documented resection with optimal histologic assessment**, while tailoring the technique to procedural risk, lesion characteristics, and patient factors, in line with the strength of the recommendation and the quality of supporting evidence.

Case 4.20

Vignette
A 14 mm nonpedunculated adenomatous colorectal polyp is detected.

Question
What is the standard resection technique recommended for this lesion? In your answer, explicitly take into account the strength of the recommendation and the quality of the evidence supporting it, and calibrate the wording accordingly.

Most appropriate management: hot snare EMR as the standard resection technique

For a **14 mm nonpedunculated adenomatous colorectal polyp**, **hot snare endoscopic mucosal resection (EMR) is the standard recommended resection technique**.

This represents a **strong recommendation** supported by **moderate-quality evidence**, based on comparative studies, large prospective series, and guideline consensus indicating that hot snare EMR provides **high rates of complete resection**with acceptable safety for nonpedunculated adenomas in the 10–19 mm range. Randomized data are limited, but the consistency of outcomes across studies supports this approach as standard practice.

Accordingly, **hot snare EMR should usually be performed**, aiming for **en bloc resection when technically feasible**. If en bloc removal is not achievable, **piecemeal EMR** can be used, with careful inspection of margins and appropriate post-resection surveillance.

Alternative approaches may be considered in selected situations:

- **Cold snare resection** may be reasonable in carefully selected low-risk lesions when minimizing thermal injury is a priority, although supporting evidence is less robust for this size category.
- **ESD** is generally not indicated for typical adenomatous lesions of this size unless specific features raise concern for superficial submucosal invasion and en bloc resection is essential.

Overall, **hot snare EMR remains the standard technique** for a 14 mm nonpedunculated adenomatous polyp, with technique choice individualized based on lesion characteristics, patient factors, and operator expertise, consistent with the strength of the recommendation and the quality of available evidence.

Case 4.21

Vignette
Hot snare polypectomy is planned for a 15 mm adenomatous polyp.

Question
Should submucosal injection be considered to reduce the risk of thermal injury? In your answer, explicitly take into account the strength of the recommendation and the quality of the evidence supporting it, and calibrate the wording accordingly.

Most appropriate management: consider submucosal injection to reduce thermal injury risk

When **hot snare polypectomy is planned for a 15 mm adenomatous colorectal polyp**, **submucosal injection should generally be considered** to reduce the risk of **deep thermal injury and perforation**.

This represents a **strong recommendation** supported by **moderate-quality evidence**, based on randomized and observational studies showing that creation of a **submucosal cushion** separates the lesion from the muscularis propria, thereby **reducing thermal damage** and improving safety, particularly for nonpedunculated polyps of this size.

Accordingly, **submucosal injection (EMR technique)** is usually appropriate before hot snare transection, especially for:

- **sessile or flat lesions**,
- polyps located in the **right colon**, where the wall is thinner,
- situations where en bloc resection is planned and controlled snare capture is desired.

While the evidence supports routine consideration of injection in this context, it does **not mandate injection in every case**. For clearly pedunculated lesions with a well-defined stalk, injection may be unnecessary, and technique should be adapted accordingly.

Overall, **use of submucosal injection is recommended in most cases** of hot snare resection for 15 mm adenomatous polyps to enhance procedural safety, with final decisions **individualized** based on lesion morphology, location, and operator experience, in keeping with the strength of the recommendation and the quality of available evidence.

Case 4.22

Vignette
A 35 mm nonpedunculated adenomatous colorectal polyp is identified.

Question
What is the recommended endoscopic resection technique for this lesion? In your answer, explicitly take into account the strength of the recommendation and the quality of the evidence supporting it, and calibrate the wording accordingly.

Most appropriate management: endoscopic mucosal resection as first-line therapy, with technique tailored to lesion characteristics

For a **35 mm nonpedunculated adenomatous colorectal polyp**, **endoscopic resection is recommended as first-line management**, provided that **optical assessment does not suggest deep submucosal invasion**.

This corresponds to a **strong recommendation** supported by **moderate-quality evidence**, based on large observational series, comparative studies, and guideline consensus demonstrating that **endoscopic mucosal resection (EMR)** achieves high rates of curative resection with an acceptable safety profile for large nonpedunculated adenomas. Randomized trials are limited, but the consistency of real-world outcomes supports this approach.

Accordingly, the **standard technique is hot snare EMR**, typically performed **piecemeal** for lesions of this size, with:

- **Submucosal injection** to create a safety cushion and improve resection control,
- **Careful inspection and treatment of resection margins**,
- **Planned surveillance** to detect and treat residual or recurrent adenoma.

Endoscopic submucosal dissection (ESD) may be **considered selectively**, particularly when:

- **en bloc resection is required** for accurate histologic assessment,
- there are **features suspicious for superficial submucosal invasion** but not deep invasion,
- or in specialized centers with appropriate expertise.

However, ESD is associated with **longer procedure times and higher complication rates**, and therefore is **not routinely required** for typical large adenomatous lesions without high-risk features.

In summary, **hot snare EMR is the recommended and standard resection technique** for a 35 mm nonpedunculated adenomatous polyp, with **selective use of ESD in carefully chosen cases**, consistent with the strength of the recommendation and the quality of available evidence.

Case 4.23

Vignette
A large nonpedunculated colorectal polyp ≥20 mm is referred to a high-volume expert center.

Question
Can endoscopic submucosal dissection be considered as an alternative to EMR? In your answer, explicitly take into account the strength of the recommendation and the quality of the evidence supporting it, and calibrate the wording accordingly.

Most appropriate management: consider ESD selectively as an alternative to EMR in expert centers

For a **large (≥20 mm) nonpedunculated colorectal polyp** referred to a **high-volume expert center**, **endoscopic submucosal dissection (ESD) can be considered as an alternative to endoscopic mucosal resection (EMR)** in **selected cases**.

This corresponds to a **weak recommendation** supported by **moderate-quality evidence**, based on comparative observational studies and meta-analyses showing that ESD provides **higher en bloc and R0 resection rates** and **lower local recurrence** than EMR, at the cost of **longer procedure times and higher rates of adverse events**, particularly perforation. Randomized comparative trials are limited, which explains the conditional nature of the recommendation.

Accordingly, **ESD may be appropriate** when one or more of the following apply:

- **En bloc resection is required** for accurate histopathologic assessment (for example, lesions with suspected superficial submucosal invasion).
- The lesion has **features associated with higher recurrence after EMR** (such as non-granular morphology, pseudodepression, or significant fibrosis).
- The procedure is performed in a **specialized center** with substantial ESD expertise and appropriate peri-procedural support.

Conversely, **EMR remains the preferred first-line technique** for most large nonpedunculated adenomatous lesions without features suspicious for submucosal invasion, given its **shorter procedure time, lower complication rate, and broad applicability**.

In summary, while ESD is **not routinely required** for all large colorectal polyps, it represents a **valid alternative to EMR in carefully selected cases** managed in expert centers. The choice between EMR and ESD should be **individualized**, balancing lesion characteristics, patient factors, local expertise, and the moderate certainty of the supporting evidence.

Case 4.24

Vignette
After EMR, small areas of residual adenoma remain that cannot be captured with a snare.

Question
What is the optimal adjunctive technique for removal of residual neoplasia? In your answer, explicitly take into account the strength of the recommendation and the quality of the evidence supporting it, and calibrate the wording accordingly.

Most appropriate management: adjunctive thermal ablation of residual adenoma

When **small areas of residual adenoma remain after EMR** and **cannot be captured with a snare**, **adjunctive thermal ablation is recommended** to eradicate residual neoplastic tissue.

This represents a **strong recommendation** supported by **moderate-quality evidence**, based on randomized trials and comparative studies showing that **thermal ablation of residual tissue significantly reduces local recurrence rates**compared with no adjunctive treatment. Although the evidence base is not derived from large multicenter randomized trials for every modality, results are consistent across studies.

Accordingly, the **preferred technique is thermal ablation**, most commonly using:

- **Snare tip soft coagulation (STSC)** applied to the resection margins and residual islands of tissue.

Alternative thermal modalities (for example, argon plasma coagulation) **may be considered**, but STSC is generally favored because it allows **controlled, focal application** with good efficacy and a favorable safety profile.

It should be emphasized that **thermal ablation should be applied only to clearly residual adenomatous tissue** after careful inspection, and not as a substitute for snare resection when snaring is technically feasible.

Overall, adjunctive thermal ablation represents the **optimal strategy for managing small residual adenoma after EMR**, balancing efficacy and safety in line with the strength of the recommendation and the quality of the supporting evidence.

Case 4.25

Vignette
Visible residual neoplastic tissue remains after EMR, and argon plasma coagulation is considered.

Question
Is argon plasma coagulation recommended in this situation?
In your answer, explicitly take into account the strength of the recommendation and the quality of the evidence supporting it, and calibrate the wording accordingly.

Most appropriate management: avoid APC for visible residual neoplasia; prefer resection or targeted thermal ablation

When **visible residual neoplastic tissue remains after EMR**, **argon plasma coagulation (APC) is generally not recommended** as the primary treatment for that tissue.

This reflects a **strong recommendation** supported by **moderate-quality evidence**, based on comparative studies and guideline consensus showing that APC applied to **visible adenomatous tissue** is associated with **higher recurrence rates**than techniques that physically remove or precisely ablate the residual lesion. APC is a non-contact modality that does not allow histologic confirmation and may incompletely treat residual neoplasia when tissue is clearly visible.

Accordingly, the preferred approach is to:

- **Resect residual tissue with a snare** whenever technically feasible, or
- Use **targeted thermal ablation**, most commonly **snare tip soft coagulation (STSC)**, for **small residual areas that cannot be captured by a snare**.

APC may be considered only in a limited, adjunctive role, such as for:

- very small, flat residual areas **after complete snare resection**, or
- treatment of the **resection margin** when no visible neoplastic tissue remains.

Even in these situations, the supporting evidence is limited, and APC should be applied cautiously.

In summary, when **visible residual neoplasia is present**, APC **should not be used as the primary treatment**. Complete removal by snare or precise thermal ablation is preferred, aligning with the strength of the recommendation and the quality of the available evidence.

Case 4.26

Vignette
A large colorectal polyp is removed by piecemeal EMR.

Question
How should the resection margins be managed to reduce the risk of adenoma recurrence? In your answer, explicitly take into account the strength of the recommendation and the quality of the evidence supporting it, and calibrate the wording accordingly.

Most appropriate management: routine thermal ablation of resection margins after piecemeal EMR

After **piecemeal EMR of a large colorectal polyp**, **systematic management of the resection margins is recommended**to reduce the risk of adenoma recurrence.

This represents a **strong recommendation** supported by **moderate-quality evidence**, based on randomized controlled trials and comparative studies demonstrating that **thermal ablation of the post-EMR margins significantly lowers local recurrence rates** compared with no margin treatment. Although evidence does not derive from large multicenter trials for every technique, results are consistent across studies.

Accordingly, the preferred approach is to **perform routine thermal ablation of the entire resection margin**, most commonly using **snare tip soft coagulation (STSC)**, once all visible neoplastic tissue has been removed. This technique targets microscopic residual adenoma at the margin that is not endoscopically visible.

Key principles include:

- Apply ablation **only after complete macroscopic resection** has been achieved.
- Ensure **systematic circumferential treatment** of the margin.
- Avoid relying on margin ablation as a substitute for resection of visible residual tissue.

Alternative modalities, such as argon plasma coagulation, are **less favored** for margin treatment because of higher recurrence rates and less controlled tissue effect.

Overall, **routine margin ablation following piecemeal EMR** is the optimal strategy to minimize adenoma recurrence, while maintaining a favorable balance between efficacy and safety, in line with the strength of the recommendation and the quality of the supporting evidence.

Case 4.27

Vignette
An endoscopist considers underwater EMR instead of conventional hot EMR for a large adenomatous lesion.

Question
Is underwater EMR an acceptable alternative in this setting?
In your answer, explicitly take into account the strength of the recommendation and the quality of the evidence supporting it, and calibrate the wording accordingly.

Most appropriate management: underwater EMR may be considered as an alternative in selected cases

For a **large adenomatous colorectal lesion**, **underwater EMR (UEMR) can be considered as an alternative to conventional hot EMR**, particularly in selected cases.

This corresponds to a **weak recommendation** supported by **moderate-quality evidence**, based on randomized trials and comparative studies showing that UEMR achieves **similar or higher en bloc resection rates** and **comparable safety** to conventional EMR for certain lesion sizes and morphologies, without the need for submucosal injection. However, evidence is heterogeneous and long-term recurrence data are more limited.

Accordingly, **UEMR may be appropriate** when:

- the lesion is **nonpedunculated and suitable for underwater capture**,
- avoiding submucosal injection is desirable,
- and the procedure is performed by **endoscopists experienced with the technique**.

Conversely, **conventional hot EMR remains the standard approach** for most large adenomatous lesions, given its broad evidence base, reproducibility, and established outcomes. UEMR should therefore **not be considered a universal replacement**, but rather a **selective alternative**.

In summary, while UEMR is an **acceptable option in selected settings**, its use should be **individualized**, balancing lesion characteristics, operator expertise, and the moderate certainty of the supporting evidence.

Case 4.28

Vignette
An endoscopist wants to define whether an EMR procedure was successful.

Question
Which criteria should be used to define successful EMR?
In your answer, explicitly take into account the strength of the recommendation and the quality of the evidence supporting it, and calibrate the wording accordingly.

Most appropriate management: define EMR success using combined technical, endoscopic, and histologic criteria

To determine whether an **endoscopic mucosal resection (EMR)** procedure was **successful**, **a composite set of criteria should be used**, integrating **technical completeness, endoscopic assessment, and histopathologic findings**.

This represents a **strong recommendation** supported by **moderate-quality evidence**, based on prospective cohorts, comparative studies, and guideline consensus showing that **no single criterion is sufficient** to define success and that a combined definition better predicts recurrence and clinical outcomes.

Accordingly, **successful EMR is generally defined by all of the following**:

1. **Complete macroscopic resection at index procedure**
   - No visible residual neoplastic tissue at the end of the procedure after careful inspection using white-light and image-enhanced endoscopy.
2. **Appropriate histopathologic assessment**
   - For en bloc resections: confirmation of **complete excision (R0)** when assessable.
   - For piecemeal resections: histology confirming the **expected lesion type** without features mandating surgery, recognizing that margin status cannot be reliably assessed.
3. **Absence of residual or recurrent neoplasia at first surveillance**
   - A negative site-check colonoscopy (typically at **3–6 months** after piecemeal EMR) is an essential component of defining durable success.
4. **No procedure-related adverse events requiring escalation of care**
   - Particularly perforation or uncontrolled bleeding that compromises the intended curative approach.

While the recommendation to use a composite definition is strong, it is important to acknowledge that **evidence quality is moderate** and that definitions vary slightly across studies. Therefore, reports and audits **should explicitly state which criteria are used**, and uncertainty should be acknowledged, especially after piecemeal EMR.

In summary, **successful EMR should be defined by complete macroscopic removal, appropriate histology, and confirmation of no residual disease at early follow-up**, rather than by the index procedure alone, aligning clinical practice with the strength of recommendations and the available evidence.

Case 4.29

Vignette
A sessile serrated lesion ≥20 mm without suspected dysplasia is identified.

Question
What is the recommended endoscopic resection strategy for this lesion? In your answer, explicitly take into account the strength of the recommendation and the quality of the evidence supporting it, and calibrate the wording accordingly.

Most appropriate management: endoscopic mucosal resection as first-line therapy, with technique tailored to safety

For a **sessile serrated lesion (SSL) ≥20 mm without suspected dysplasia**, **endoscopic resection is recommended as first-line management**, provided that **optical assessment does not suggest submucosal invasion**.

This represents a **strong recommendation** supported by **moderate-quality evidence**, based on prospective cohorts, comparative studies, and guideline consensus showing that large non-dysplastic SSLs have a **low risk of deep invasion**and can be **safely and effectively treated endoscopically**. Randomized data are limited, but outcomes across studies consistently support this approach.

Accordingly, the **preferred strategy is endoscopic mucosal resection (EMR)**, most often performed **piecemeal** for lesions of this size, with:

- **Careful optical diagnosis** confirming absence of dysplasia or invasive features,
- **Complete macroscopic resection**, and
- **Planned early surveillance** to detect and treat residual or recurrent tissue.

Cold snare piecemeal resection may be **considered in selected cases**, particularly to reduce thermal injury and bleeding risk, especially in the right colon, but the supporting evidence is less robust and long-term recurrence data are more limited. **ESD is generally not required** for non-dysplastic SSLs and should be reserved for selected situations where en bloc resection is deemed necessary.

Overall, **piecemeal EMR remains the recommended standard approach** for SSLs ≥20 mm without suspected dysplasia, with technique selection individualized based on lesion characteristics, patient factors, and local expertise, in keeping with the strength of the recommendation and the quality of available evidence.

Supplementary Material C. Supplementary Coding Guide for Calibration Assessment

Operational definition of calibration

Calibration was defined as the alignment between the level of certainty expressed in the LLM response and both the strength of recommendation and the quality of supporting evidence. A response was considered calibrated only if alignment was maintained with both dimensions.

Scoring criteria (binary classification)

- **Score = 1 (calibrated):** the response uses wording consistent with the underlying certainty level (e.g., cautious or conditional language for weak recommendations or low-quality evidence; assertive language for strong recommendations supported by high-quality evidence).
- **Score = 0 (not calibrated):** the response uses overly assertive or prescriptive language despite weak recommendations or low-quality evidence, or otherwise fails to appropriately reflect the level of certainty.

Examples

- *Calibrated:* "This approach may be considered…" (weak recommendation, low-quality evidence)
- *Not calibrated:* "This approach should be performed…" (weak recommendation, low-quality evidence)

These examples are illustrative; full response-level data are provided in the Supplementary dataset.

Supplementary Table 1. Statement-level adherence and calibration of AI-generated responses to ESGE colorectal guidelines.

Guideline	Statement	Recommendation strength	Quality of evidence	Adherence (0/1)	Calibration (0/1)	Rationale
1. Endoscopic surveillance after surgical or endoscopic resection for colorectal cancer (ESGE–ESDO)	1.1 We recommend post-surgery endoscopic surveillance for CRC patients after intent-to-cure surgery and appropriate oncological treatment for both local and distant disease.	Strong	Low	1	1	The answer recommends active surveillance including colonoscopic surveillance, consistent with offering post-surgery endoscopic surveillance. Wording is appropriately directive for a strong recommendation.
	1.2 We recommend a high quality perioperative colonoscopy before surgery for CRC or within 6 months following surgery.	Strong	Low	1	1	Recommends repeating a high-quality complete colonic evaluation pre-op (or validated alternatives if incomplete), aligning with the statement. Wording is suitably strong.
	1.3 We recommend performing surveillance colonoscopy 1 year after CRC surgery.	Strong	Moderate	1	1	Explicitly recommends colonoscopy at 1 year. Strong language is coherent with a strong recommendation and moderate evidence.
	1.4 We do not recommend an intensive endoscopic surveillance strategy, e.g. annual colonoscopy, because of a lack of proven benefit.	Strong	Moderate	1	1	Clearly advises against annual colonoscopy and proposes a non-intensive schedule. Wording matches a strong "do not" recommendation.
	1.5 After the first surveillance colonoscopy following CRC surgery, we suggest the second colonoscopy should be performed 3 years later, and the third 5 years after the second. If additional high risk neoplastic lesions are detected, subsequent surveillance examinations at shorter intervals may be considered.	Weak	Low	1	0	Content aligns (3 years then 5 years), but wording is presented as a firm standard (little conditionality, no "may be considered" tone), which is not well calibrated to **weak/low**.
	1.6 After the initial surveillance colonoscopy, we suggest halting post-surgery endoscopic surveillance at the age of 80 years, or earlier if life-expectancy is thought to be limited by comorbidities.	Weak	Low	1	0	Decision (stop surveillance) is consistent with the statement in this scenario, but wording is very definitive ("discontinue further scheduled colonoscopies") rather than conditional/shared decision framing expected for **weak/low**.

	1.7 In patients with a low risk pT1 CRC treated by endoscopy with an R0 resection, we suggest the same endoscopic surveillance schedule as for any CRC.	Weak	Low	0	0	The answer proposes a different schedule (6 to 12 months and post-polypectomy style follow-up), which is not “the same schedule as for any CRC” per the statement. Also uses “standard of care” wording, not calibrated to **weak/low**

Guideline	Statement	Strength of recommendation	Quality of evidence	Adherence (0/1)	Calibration (0/1)	Rationale
2. Post-polypectomy colonoscopy surveillance (ESGE 2020)	2.1 ESGE recommends that patients with complete removal of 1–4 <10 mm adenomas with low grade dysplasia, irrespective of villous components, or any serrated polyp <10 mm without dysplasia, do not require endoscopic surveillance and should be returned to screening.	Strong	Moderate	1	1	The response clearly states that no surveillance colonoscopy is indicated and that the patient should be returned to screening. The wording is appropriately directive for a strong recommendation with moderate-quality evidence.
	2.2 If organized screening is not available, repetition of colonoscopy 10 years after the index procedure is recommended.	Strong	Moderate	1	1	The response explicitly recommends a 10-year colonoscopy interval as a substitute for organized screening. Language is firm and consistent with a strong recommendation.
	2.3 ESGE recommends surveillance colonoscopy after 3 years for patients with complete removal of at least 1 adenoma ≥10 mm or with high grade dysplasia, or ≥5 adenomas, or any serrated polyp ≥10 mm or with dysplasia.	Strong	Moderate	1	1	The answer recommends a 3-year surveillance interval and frames it as indicated due to increased risk. Wording is well calibrated to a strong recommendation.
	2.4 ESGE recommends a 3–6-month early repeat colonoscopy following piecemeal endoscopic resection of polyps ≥20 mm.	Strong	Moderate	1	1	The response clearly recommends a 3–6 month repeat colonoscopy and justifies intensified follow-up. This is consistent with both content and strength of the recommendation.
	2.5 A first surveillance colonoscopy 12 months after the repeat	Strong	High	1	1	The answer correctly identifies the need for a 12-month surveillance colonoscopy after a negative early repeat exam. Strong, directive

	colonoscopy is recommended to detect late recurrence.					wording is appropriate given the high-quality evidence.
	2.6 If no polyps requiring surveillance are detected at the first surveillance colonoscopy, ESGE suggests to perform a second surveillance colonoscopy after 5 years. After that, if no polyps requiring surveillance are detected, patients can be returned to screening.	Weak	Low	1	0	While the timing (5 years) is correct, the wording presents this strategy as a clearly appropriate default without emphasizing conditionality or uncertainty. This represents **overselling** relative to a weak, low-quality recommendation.
	2.7 ESGE suggests that, if polyps requiring surveillance are detected at first or subsequent surveillance examinations, surveillance colonoscopy may be performed at 3 years.	Weak	Low	1	0	The response correctly resets surveillance based on newly detected lesions but uses fairly prescriptive language ("must be recalculated", "reset") with little acknowledgement of conditionality, resulting in suboptimal calibration for a weak recommendation.
	2.8 ESGE suggests stopping post-polypectomy endoscopic surveillance at the age of 80 years, or earlier if life expectancy is thought to be limited by co-morbidities.	Weak	Low	1	0	The decision to stop surveillance is guideline-concordant, but the wording is definitive ("discontinuation is appropriate", "no further scheduled colonoscopies") rather than conditional or preference-sensitive, leading to poor calibration for weak/low evidence.

Guideline	Statement	Strength of recommendation	Quality of evidence	Adherence (0/1)	Calibration (0/1)	Rationale
3. Endoscopic management of Lynch syndrome and of familial risk of colorectal cancer (ESGE)	3.1 ESGE recommends that individuals with Lynch syndrome should be followed in dedicated units that practice monitoring of compliance and endoscopic performance measures.	Strong	Low	1	1	The response explicitly recommends centralized, structured surveillance in expert centers with monitoring of quality indicators. Directive wording is appropriate for a strong recommendation, despite low-quality evidence.
	3.2 ESGE recommends starting colonoscopy surveillance at the age of 25 years for MLH1 and MSH2 mutation carriers and at the age of 35 years for MSH6 and PMS2 mutation carriers.	Strong	Moderate	1	1	The answer clearly recommends immediate initiation of colonoscopic surveillance in a 26-year-old MLH1 carrier, with firm wording consistent with a strong recommendation.
	3.3 ESGE recommends a high quality surveillance colonoscopy every 2 years in asymptomatic individuals with Lynch syndrome.	Strong	Moderate	1	0	Although recommending intensive surveillance, the response systematically favors a 1-year interval and frames it as generally preferred, overselling beyond the guideline-mandated 2-year interval.
	3.4 ESGE recommends to repeat complete colonoscopy within 3 months in the case of a colonoscopy of suboptimal quality (poor bowel preparation or incomplete procedure).	Strong	Moderate	1	1	The response recommends prompt repeat colonoscopy and correctly resets surveillance timing. Wording is appropriately directive for a strong recommendation.
	3.5 ESGE recommends performance of endoscopy earlier than the planned surveillance procedure if an individual with Lynch syndrome is symptomatic.	Strong	Low	1	1	The answer clearly prioritizes immediate diagnostic colonoscopy outside routine surveillance. Strong wording is appropriate given the recommendation strength.
	3.6 ESGE recommends the routine use of high-definition endoscopy systems in individuals with Lynch syndrome.	Strong	High	1	1	The response strongly recommends high-definition endoscopy and frames it as standard of care, which is coherent with strong/high-quality evidence.
	3.7 ESGE suggests the use of chromoendoscopy may be of benefit in individuals with Lynch syndrome undergoing colonoscopy; however routine use must be balanced against	Weak	Moderate	1	1	The response correctly presents chromoendoscopy as optional and adjunctive, emphasizing balance with feasibility and overall quality. This wording is well calibrated to a weak recommendation.

	costs, training, and practical considerations.					
	3.8 ESGE does not recommend routine gastric surveillance in individuals with Lynch syndrome.	Strong	Low	0	0	The response proposes individualized and risk-adapted upper GI surveillance rather than clearly advising against routine gastric surveillance, deviating from the statement. Wording also dilutes a strong "do not recommend".
	3.9 ESGE suggests (non-invasive) testing for *Helicobacter pylori* in individuals with Lynch syndrome.	Weak	Moderate	1	0	Although testing is recommended, the response uses prescriptive language ("should be offered to all"), overselling a weak recommendation.
	3.10 ESGE does not recommend routine small-bowel surveillance in individuals with Lynch syndrome.	Strong	Moderate	1	1	The response clearly advises against routine small-bowel surveillance and reserves testing for symptoms or selected cases, appropriately matching a strong negative recommendation.
	3.11 ESGE recommends definition of familial risk of colorectal cancer as the presence of at least two first-degree relatives with colorectal cancer or at least one first-degree relative with colorectal cancer before the age of 50 years.	Strong	Moderate	1	1	The answer correctly classifies the scenario as increased familial risk and uses firm wording consistent with a strong definitional recommendation.
	3.12 ESGE recommends colonoscopy surveillance in first-degree relatives of colorectal cancer patients in families that fulfill the definition of familial risk of colorectal cancer.	Strong	Moderate	1	1	The response recommends intensified colonoscopic surveillance with appropriate intervals. Language is directive and consistent with strong recommendation.
	3.13 ESGE recommends starting colonoscopy surveillance at the age of 40 years when there is a familial risk of colorectal cancer.	Strong	Moderate	1	1	Immediate initiation of surveillance at age 41 is recommended with firm wording, well aligned with the statement.
	3.14 ESGE recommends a 5-year surveillance interval for colonoscopy after a normal high quality baseline examination in the setting of familial risk of colorectal cancer.	Strong	Low	1	1	The response recommends continued 5-year surveillance despite a negative baseline exam. Directive wording is acceptable for a strong recommendation, even with low-quality evidence.

	3.15 ESGE recommends that follow-up after polyp excision in individuals with familial risk of colorectal cancer should follow the surveillance guidelines for the general population.	Strong	Moderate	1	1	The response correctly prioritizes polyp characteristics over familial risk and applies standard post-polypectomy surveillance, with appropriate wording.

Guideline	Statement	Strength of recommendation	Quality of evidence	Adherence (0/1)	Calibration (0/1)	Rationale
4. Colorectal polypectomy and endoscopic mucosal resection (ESGE)	4.1 ESGE recommends the performance and reporting of optical diagnosis, including polyp pit and vascular patterns, as recommended by the ESGE curriculum. This should be integrated with the macroscopic features and location to predict the risk of submucosal invasion.	Strong	Moderate	1	1	The answer systematically recommends integrated optical diagnosis using WLE and virtual chromoendoscopy. The directive wording is appropriate for a strong recommendation supported by moderate-quality evidence.
	4.2 ESGE recommends that polyps should be described and reported according to their location, size (in millimeters), and morphology (Paris classification). Nonpolypoid lesions ≥10mm (laterally spreading lesions) should also be classified as: (a) granular homogeneous; (b) granular nodular-mixed; (c) nongranular elevated; (d) nongranular pseudodepressed.	Strong	Moderate	1	1	The response explicitly details all required reporting elements and uses firm language consistent with a strong recommendation.
	4.3 ESGE recommends the use of high definition white-light endoscopy in combination with virtual chromoendoscopy to detect the presence and predict the depth of submucosal invasion in large (≥20mm) nonpedunculated colorectal polyps (LNPCPs) prior to selection of a treatment strategy.	Strong	High	1	1	The answer clearly mandates combined HD-WLE and virtual chromoendoscopy before treatment. Strong, prescriptive wording is appropriate given high-quality evidence.
	4.4 ESGE recommends that polyps with endoscopic imaging characteristics of deep submucosal invasion should be discussed by the multidisciplinary team before treatment.	Strong	Moderate	1	1	The response advises abandoning endoscopic resection and referring for multidisciplinary and surgical evaluation. Language matches a strong recommendation.
	4.5 ESGE recommends retrieval and histopathologic analysis of resected polyps. A resect-and-discard strategy using real-time optical diagnosis with	Strong	High	1	1	The answer prioritizes histology and restricts discard strategies to selected expert settings. Wording is appropriately firm.

	virtual or dye-based chromoendoscopy for diminutive colorectal polyps should be reserved for experts only.					
	4.6 ESGE recommends the use of high quality photo- and/or videodocumentation in preference to biopsies to determine the most advanced pathology in LNPCPs and to inform selection of the optimal treatment strategy or facilitate tertiary referral. Where deep submucosal invasion is suspected, biopsies are indicated.	Strong	High	1	1	The response clearly discourages routine biopsies and prioritizes documentation, with strong directive language appropriate to the recommendation.
	4.7 ESGE recommends resection of all polyps with the exception of diminutive (≤5mm) rectosigmoid polyps that are predicted to be nonadenomatous with high confidence.	Strong	High	1	1	The answer correctly endorses a diagnose-and-leave strategy only in the specified scenario, with firm wording aligned to a strong recommendation.
	4.8 ESGE recommends hot snare polypectomy for pedunculated polyps.	Strong	High	1	1	Hot snare polypectomy is presented as the standard technique. Language is appropriately definitive.
	4.9 ESGE suggests the use of dilute adrenaline injection and/or mechanical hemostasis in pedunculated colorectal polyps with a head size of ≥2 cm or a stalk width of ≥1 cm to prevent immediate post-polypectomy bleeding.	Weak	Low	1	0	Although content is correct, the wording frames prophylactic hemostasis as routinely recommended rather than optional or conditional, overselling a weak, low-evidence recommendation.
	4.10 ESGE recommends that the majority of colonic and rectal lesions can be effectively removed in a curative way by standard polypectomy and/or by EMR.	Strong	High	1	1	The answer clearly supports endoscopic management for most lesions using strong, guideline-consistent language.
	4.11 ESGE recommends cold snare polypectomy for the removal of diminutive polyps (≤5 mm).	Strong	High	1	1	The response unequivocally recommends cold snare polypectomy, appropriately reflecting a strong recommendation.
	4.12 ESGE recommends including a clear margin of normal tissue (1–2mm) surrounding the polyp.	Strong	High	1	1	The answer clearly instructs inclusion of a normal tissue margin, with directive wording matching the statement strength.
	4.13 ESGE recommends against the use of hot biopsy forceps because of its	Strong	High	1	1	The response strongly discourages hot biopsy forceps, fully aligned in content and tone.

	high rates of incomplete resection, inadequate tissue sampling for histopathologic examination, and the unacceptably high risk of adverse events (deep thermal injury and delayed bleeding) in comparison with cold snare excision.					
	4.14 ESGE recommends against the use of cold biopsy forceps excision because of its high rate of incomplete resection.	Strong	Moderate	1	1	Cold biopsy forceps are discouraged as standard practice, with firm wording consistent with a strong recommendation.
	4.15 ESGE recommends cold snare polypectomy for the removal of small polyps (6–9mm).	Strong	High	1	1	The response clearly recommends cold snare polypectomy with appropriate strength of language.
	4.16 ESGE suggests consideration of piecemeal cold snare EMR for carefully selected flat adenomas of 10–19mm (granular homogeneous LNPCPs), particularly in the right colon and especially when co-morbidity levels are high, to reduce the risks of deep mural injury and delayed post-EMR bleeding.	Weak	Low	1	0	While the strategy is correct, the wording presents pCSP as an acceptable default rather than a conditional option, overselling a weak, low-quality recommendation.
	4.17 ESGE recommends piecemeal cold snare polypectomy (pCSP) for sessile serrated lesions (SSLs) without dysplasia of 10–19mm in size.	Strong	High	1	1	The response clearly recommends pCSP and uses directive wording consistent with strong evidence.
	4.18 ESGE suggests submucosal injection may be used prior to pCSP to facilitate tissue transection and better delineate the polyp margins.	Weak	Low	1	1	The answer appropriately frames injection as optional and situational, well calibrated to a weak recommendation.
	4.19 ESGE recommends hot snare polypectomy for the removal of SSLs with dysplasia and en bloc excision of the dysplastic component.	Weak	Low	1	0	Although management is correct, the wording is assertive and categorical, not reflecting the uncertainty inherent in a weak, low-evidence recommendation.
	4.20 ESGE recommends hot snare polypectomy (HSP) as the accepted standard of care for the removal of nonpedunculated adenomatous polyps of 10–19mm in size.	Strong	High	1	1	The response presents hot snare EMR as standard of care, consistent with the recommendation strength.

	4.21 ESGE suggests submucosal injection prior to HSP to reduce the risk of deep thermal mural injury.	Weak	Low	1	0	Submucosal injection is presented as recommended rather than optional, overselling a weak suggestion.
	4.22 ESGE recommends conventional (diathermy-based) endoscopic mucosal resection (EMR) for large (≥20mm) nonpedunculated adenomatous polyps (LNPCPs).	Strong	High	1	1	The answer clearly endorses EMR with strong, guideline-consistent language.
	4.23 Endoscopic submucosal dissection may also be suggested as an alternative for removal of LNPCPs of ≥20mm in selected cases and in high-volume centers.	Weak	Low	1	1	ESD is framed as an option in selected scenarios, with appropriately cautious wording.
	4.24 ESGE recommends that, where complete snare excision cannot be achieved, the optimal method for adjunctive removal of residual adenoma is hot avulsion or cold avulsion with adjuvant snare-tip soft coagulation (CAST). Adjunctive techniques such as CAST or hot avulsion should only be used to remove residual neoplasia that is not amenable to snare resection.	Strong	Moderate	0	0	The response recommends STSC alone, which does not reflect the guideline preference for avulsion techniques. Both content and calibration are incorrect.
	4.25 ESGE recommends against argon plasma coagulation and STSC to treat visible residual neoplasia because of its proven lack of efficacy.	Strong	Moderate	1	1	The answer clearly advises against APC for visible residual tissue, with strong negative wording appropriate to the recommendation.
	4.26 ESGE recommends that, after piecemeal EMR of LNPCPs, the resection margins should be treated by thermal ablation using snare-tip soft coagulation (STSC) to prevent adenoma recurrence.	Strong	High	1	1	Routine margin ablation with STSC is clearly recommended, with directive wording consistent with strong evidence.
	4.27 ESGE suggests that underwater EMR can be considered an alternative to conventional hot EMR for the treatment of adenomatous LNPCPs.	Weak	Moderate	1	1	The response appropriately presents underwater EMR as an alternative in selected cases, well calibrated to a weak recommendation.
	4.28 ESGE recommends that successful EMR should be defined by:	Strong	Moderate	1	1	The composite definition of success mirrors the statement, with appropriately firm wording.

	the lack of endoscopically visible remnant neoplastic tissue at the mucosectomy site; histologic assessment of the specimen; and the absence of recurrence at the first surveillance colonoscopy at 6 months.					
	4.29 ESGE recommends piecemeal cold EMR for SSLs of ≥20mm without suspected dysplasia.	Strong	Moderate	0	0	The answer recommends hot EMR rather than cold EMR, conflicting with the statement; wording also oversells a non-concordant approach.
